# Does Tranexamic Acid Reduce the Blood Loss in Various Surgeries? An Umbrella Review of State-of-the-Art Meta-Analysis

**DOI:** 10.3389/fphar.2022.887386

**Published:** 2022-05-19

**Authors:** Pan Hong, Ruikang Liu, Saroj Rai, JiaJia Liu, Yuhong Ding, Jin Li

**Affiliations:** ^1^ Department of Orthopaedic Surgery, Union Hospital, Tongji Medical College, Huazhong University of Science and Technology, Wuhan, China; ^2^ Department of Endocrinology, Union Hospital, Tongji Medical College, Huazhong University of Science and Technology, Wuhan, China; ^3^ Department of Orthopaedics and Trauma Surgery, Blue Cross Hospital, Kathmandu, Nepal; ^4^ First Clinical School, Tongji Medical College, Huazhong University of Science and Technology, Wuhan, China; ^5^ Basic Medical School, Tongji Medical College, Huazhong University of Science and Technology, Wuhan, China

**Keywords:** umbrella review, meta-analysis, antifibrinolytic agent, tranexamic acid, blood transfusion, hemostasis

## Abstract

**Background:** Tranexamic acid (TXA) has been applied in various types of surgery for hemostasis purposes. The efficacy and safety of TXA are still controversial in different surgeries. Guidelines for clinical application of TXA are needed.

**Materials and method:** We systematically searched multiple medical databases for meta-analyses examining the efficacy and safety of TXA. Types of surgery included joint replacement surgery, other orthopedic surgeries, cardiac surgery, cerebral surgery, etc. Outcomes were blood loss, blood transfusion, adverse events, re-operation rate, operative time and length of hospital stay, hemoglobin (Hb) level, and coagulation function. Assessing the methodological quality of systematic reviews 2 (AMSTAR 2) and Grading of Recommendations, Assessment, Development and Evaluation (GRADE) were used for quality assessment of the included meta-analyses. Overlapping reviews were evaluated by calculating the corrected covered area (CCA).

**Result:** In all, we identified 47 meta-analyses, of which 44 of them were of “high” quality. A total of 319 outcomes were evaluated, in which 58 outcomes were assessed as “high” quality. TXA demonstrates significant hemostatic effects in various surgeries, with lower rates of blood transfusion and re-operation, shorter operative time and length of stay, and higher Hb levels. Besides, TXA does not increase the risk of death and vascular adverse events, but it is a risk factor for seizure (a neurological event) in cardiac surgery.

**Conclusion:** Our study demonstrates that TXA has a general hemostatic effect with very few adverse events, which indicates TXA is the recommended medication to prevent excessive bleeding and reduce the blood transfusion rate. We also recommend different dosages of TXA for different types of adult surgery. However, we could not recommend a unified dosage for different surgeries due to the heterogeneity of the experimental design.

**Systematic Review Registration:** clinicaltrials.gov/, identifier CRD42021240303

## 1 Introduction

Hemorrhage is a leading cause of death in surgery and trauma (Ferraris et al., 2011; Rossaint et al., 2016), Allogeneic blood transfusion, autologous blood donation, and acute normovolemic hemodilution are the first-line treatment for hemorrhage (Murray, 2004; Nakanishi et al., 2019; Pinto et al., 2019). However, without the potential risk of serious immune reactions and viral infections, antifibrinolytic agents seem to be a safe and affordable alternative (Etchason et al., 1995). Tranexamic acid (TXA), a synthetic lysine analogue, has been frequently used for hemostasis (Henry et al., 2011; Hunt, 2015). TXA inhibits the activation of plasminogen by blocking the lysine-binding sites of plasminogen, leading to increased clot stabilization and thus reducing blood loss (Hunt, 2015).

Investigation of TXA has been a popular topic recently where numerous meta-analyses have discussed the hemostatic efficacy and safety of TXA. Hunt et al. estimated a reduction of 120,000 deaths worldwide each year if TXA was given to all patients with severe traumatic bleeding (Hunt, 2015). TXA was also demonstrated to be a safe and effective choice in total knee arthroplasty (TKA), total hip arthroplasty (THA), cardiac surgery, and other types of surgeries (Wu et al., 2018; Zhang Y. et al., 2019; Sukeik et al., 2020). Moreover, TXA has also been reported in treating postpartum hemorrhage (Novikova et al., 2015).

So far, numerous RCTs and meta-analyses have discussed TXA in various surgery types and reported significant effects. However, the methodological and statistical qualities of these studies are heterogeneous. Besides, the risks of embolism and other complications are unclear. In order to provide a comprehensive review of existing evidence, we performed an umbrella review of meta-analyses to demonstrate the efficacy and safety of TXA in various surgeries and provide a guideline for clinical application.

## 2 Methods

### 2.1 Search Strategy

Our review followed the guidelines for Meta-analyses of Observational Studies in Epidemiology and the Preferred Reporting Items for Systematic Reviews and Meta-analyses (PRISMA), and the protocol was registered in PROSPERO (registration No CRD42021240303) (Stroup et al., 2000; Moher et al., 2009). Two independent researchers (PH and JJL) searched Medline, Embase, Web of Science, and Cochrane databases updated to 15 September for meta-analyses. The detailed search strategy is shown in Supplementary Appendix S1. We processed another search at the end of the study on 16 February 2022. We would only replace the included study when the newly published study had a different conclusion. Two researchers (RL and YD) independently screened the titles and abstracts, and articles satisfying the inclusion criteria were accessed for full-text review. They independently reviewed full-text articles for eligibility. When data were incomplete, the corresponding author was contacted by email and invited to provide additional information. Reference lists of eligible reviews and meta-analyses were searched for additional citations.

### 2.2 Inclusion Criteria

We only included meta-analyses of RCTs or meta-analyses of observational studies discussing TXA. Post hoc analyses and systematic reviews without meta-analysis were excluded. Articles were included when the exposure group was divided based on the dosage or administration method, and patients who just received the placebo or saline were included in the controlled group. We included studies with comparisons of different administration methods and dosages (high versus low dose and any versus none). Bayesian meta- and network meta-analysis were excluded unless we could extract the complete statistical data separately from a subgroup analysis. Included studies must be in accordance with the Declaration of Helsinki and approved by the respective ethics committees.

### 2.3 Data Extraction

Two researchers (SR and JJL) independently extracted data from eligible articles. Our data extraction methodology was recommended and revised by the Joanna Briggs Institute (Supplementary Appendix S2) (Aromataris et al., 2014). Outcomes were classified as blood loss (total blood loss, intraoperative blood loss, and postoperative blood loss), risk of adverse effects (risk of massive hemorrhage, risk of transfusion requirement, risk of postoperative edema and ecchymosis, risk of rebleeding, and risk of significant adverse effects), and other outcomes (surgical field score, satisfaction with the surgical field, operative time, intraoperative blood pressure, hemoglobin drop, hematocrit drop, and length of hospital stay). We only compared the preoperative dose, which was more important than the maintenance dose. Considering that more variables would bring heterogeneity and that the maintenance dose was also correlated with the preoperative dose, we did not compare the maintenance dose. If an article presented separate meta-analyses for more than one health outcome, we included each of these separately. Standard mean difference (SMD) or weighted mean difference (WMD) was used for continuous variable statistics, and relative risks (RR), odds ratios (OR), or risk difference (RD) was used for discontinuous variable statistics.

### 2.4 Quality Assessment

We used “assessing the methodological quality of systematic reviews (AMSTAR) 2” to assess the methodological quality of the included meta-analyses (Supplementary Appendix S3) (Shea et al., 2017). We also used the Grading of Recommendations, Assessment, Development and Evaluation (GRADE) tool to evaluate the quality of evidence for each included outcome **(** Supplementary Appendix S4 **)** (Guyatt et al., 2011). The GRADE tool classifies evidence for outcomes from systematic reviews and meta-analyses into “high,” “moderate,” “low,” and “very low.” The study design of the included studies decides the baseline quality of evidence, but other factors can decrease or increase the quality level (for the detailed appraisal rule, see Supplementary Appendix S4). Two researchers (RL and JJL) rated the methodological quality of the reviews.

### 2.5 Statistical Analysis

The heterogeneity in the results of meta-analyses was assessed by chi-square and I^2^ tests, followed by appropriate analysis models (fixed-effect or random-effect). Chi-squared *p* < 0.05 and I^2^ >50% indicated high heterogeneity, and random-effects models were used in these outcomes, whereas chi-squared *p* > 0.05 and I^2^<50% were considered acceptable heterogeneity, and a fixed-effects model was used instead. Most of the meta-analyses included in our review used software (Stata and RevMan) to process their statistical analyses. Publication bias was evaluated by funnel plots and further confirmed by Egger’s and Begg’s test, and *p* < 0.05 was considered as the statistically significant risk of bias (Sterne et al., 2000). Small-study effects that lead to potential reporting or publication bias could also be avoided by Egger’s test. However, if the data of Egger’s test were not reported, then the meta-analyses, including no more than five studies, were considered high publication bias. Sensitivity analysis was performed in the meta-analysis by excluding each study one at a time to check whether the effectiveness of the outcome was determined by individual studies. Overlapping reviews happened frequently when two or more studies evaluated the same exposure and outcome. To determine the potential overlapping, a graphical cross-tabulation was made to calculate the corrected covered area (CCA), which could quantify the degree of overlap (Pieper et al., 2014). Detailed rules and standards of calculation are explained in Supplementary Appendix S5.

Besides, Shojania et al. suggested that more than 50% of systematic reviews were outdated after 5.5 years (Shojania et al., 2007). Hence, if the gap between the year of publication of potential overlapping studies is >5 years or if meta-analysis only included studies published more than 15 years ago, the previous one would be excluded directly. Update of eligible reviews was recommended by Garner et al. (2016) and satisfied the following criterion: the review achieved a minimum rating of the *Moderate* AMSTAR score. We identified newly published studies that met the inclusion criteria and matched the keywords of previous studies; the outcomes from the newly published one would change the conclusion or credibility of the review.

## 3 Result

### 3.1 Literature Search and Characteristics of Included Studies

**FIGURE 1:**
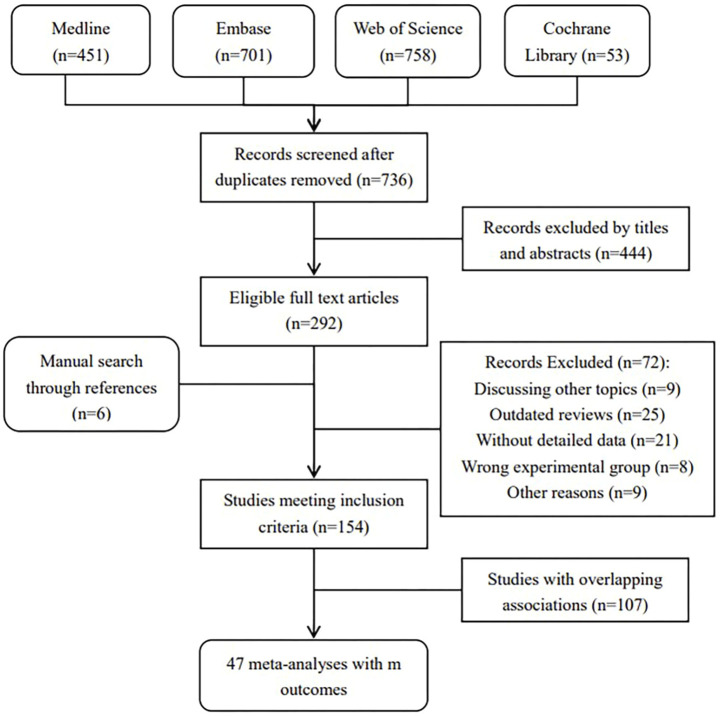
Flowchat of selection of included studies.

Figure 1 (flowchart of literature search) shows the procedure for the literature search and selection of eligible studies. Overall, 736 unique records (excluded duplicates) were searched across 4 databases, and 292 full-text articles were screened in full text. After the preliminary screening, 154 meta-analyses met the inclusion criteria. Supplementary Appendix S5 describes citation matrices of CCA for reviews with overlapping associations. In all, 107 meta-analyses reported 25 overlapping topics. After removing overlapping and outdated reviews, 47 studies were included in our umbrella review with 319 unique outcomes. Supplementary Appendices S6,S7 show the characteristics of included studies and excluded studies with their reasons for exclusion. Supplementary Appendices S8,S9 show specific data of the detailed outcomes in *Result* and *Discussion*.

The included meta-analyses were divided into eight categories as per the types of surgery: joint replacement surgery, other orthopedic surgery, cerebral surgery, cardiac surgery, nasal surgery, obstetrics and gynecological surgery, other types of surgery, and complex antifibrinolytic agents. The 47 included studies were published from 2012 to 2022, and 42 of them were published within 6 years (2016 and later). A total of 45 of 47 studies were rated as *High* quality in AMSTAR, and the remaining two were rated as *Moderate* quality. All the studies of *High* quality were meta-analyses of RCTs. AMSTAR scores for individual studies included in the umbrella review are shown in Supplementary Appendix S10. Among 319 outcomes, there were 101 outcomes assessed as at least of *Moderate* quality by GRADE, and 58 of them were rated as *High* quality. GRADE classification of the quality of evidence is listed in Supplementary Appendix S11.

### 3.2 Blood Loss

Blood loss, being the core outcome of our study, had complicated data for subgroup analysis. We divided outcomes into those of the WMD group and the SMD group. The WMD group included subgroups of total blood loss, intraoperative blood loss, and postoperative blood loss (see Table 1).

**TABLE 1:** High and moderate outcomes of blood loss (ml) with weighted mean difference (WMD).

**Author year**	**Surgery**	**Dosage**	**Characteristic**	**Outcome**	**Subgroup**	**RCTs**	**Patients**		**95% CI Estimate (WMD)**	**GRADE**
Chen et al. (2021)	Spine surgery	5–30 mg/kg	IV	PTBL	Continuous	8	674	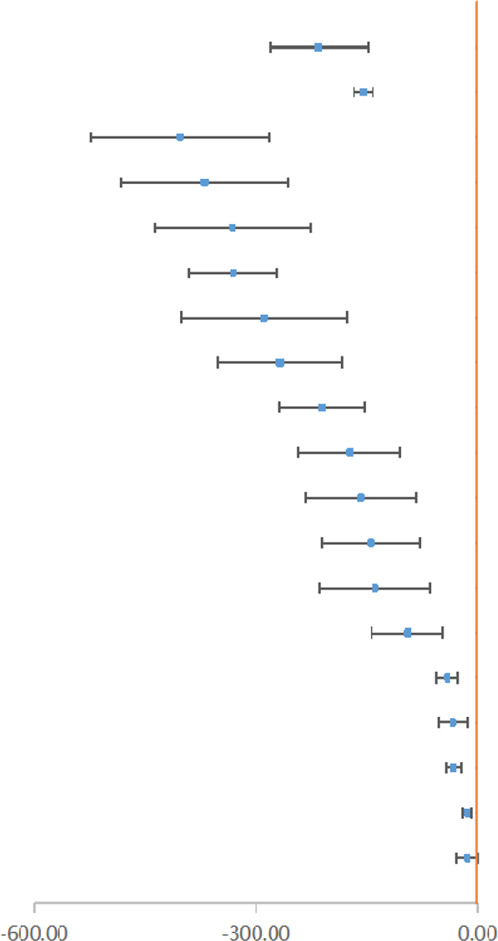	-214.68 [-281.10, -148.27]	High
Zhao et al. (2019)	OS	10–20 mg/kg	IV	IBL	Total dosage	10	655		-153.97 [-166.52, -141.41]	High
Chen et al. (2021)	Spine surgery	5–30 mg/kg	IV	PTBL	Noncontinuous	2	128		-402.52 [-522.74, -282.29]	Moderate
Huang et al. (2015)	THA	Inconsistent	IV/topical	TBL	—	11	687		-369.17 [-481.73, -256.61]	Moderate
Huang et al. (2014)	THA	Inconsistent	IV/topical/oral	TBL	—	9	488		-331.00 [-436.35, -225.65]	Moderate
Yao et al. (2019)	PAO	Inconsistent	IV	TBL	—	3	333		-330.49 [-390.16, -270.83]	Moderate
Zhao et al. (2019)	OS	10–20 mg/kg	IV	IBL	20 mg/kg	3	133		-288.90 [-400.86, -176.94]	Moderate
Chen et al. (2021)	Spine surgery	5–30 mg/kg	IV	PTBL	Any administration	10	802		-266.85 [-351.18, -182.52]	Moderate
Chen et al. (2021)	Spine surgery	5–30 mg/kg	IV	IBL	—	7	587		-210.38 [-267.31, -153.45]	Moderate
Lu et al. (2019)	IFS	1–3 g	IV	PBL	—	4	339		-172.83 [-241.43, -104.23]	Moderate
Huang et al. (2014)	THA	Inconsistent	IV/topical/oral	PBL	—	8	416		-157.85 [-232.36, -83.34]	Moderate
Lu et al. (2019)	IFS	1–3 g	IV	HBL	—	3	267		-144.20 [-210.74, -77.66]	Moderate
Zhou et al. (2019)	IFS	Inconsistent	IV	HBL	—	2	177		-139.05 [-213.67, -64.43]	Moderate
Huang et al. (2014)	Spine surgery	Inconsistent	IV/topical/oral	PBL	—	3	251		-95.12 [-142.65, -47.60]	Moderate
Xia et al. (2020)	Vaginal delivery	1 g	IV	PBL	—	2	301		-41.24 [-55.50, -26.98]	Moderate
Lu et al. (2019)	IFS	1–3 g	IV	IBL	—	4	339		-33.46 [-52.40, -14.52]	Moderate
Chan et al. (2013)	Tonsillectomy	10 mg/kg	IV/topical	TBL	—	2	180		-32.72 [-42.66, -22.78]	Moderate
Zhou et al. (2019)	IFS	Inconsistent	IV	PBL	—	3	314		-14.38 [-20.83, -7.93]	Moderate
Xia et al. (2020)	Vaginal delivery	1 g	IV	IBL	—	3	4,140		-14.30 [-28.39, -0.22]	Moderate

OS, orthognathic surgery; PAO, periacetabular osteotomy; IFS, intertrochanteric fracture surgery; PTBL, perioperative total blood loss; IBL, intraoperative blood loss; PBL, postoperative blood loss; TBL, total blood loss; HBL, hidden blood loss.

Nine types of surgeries provided WMD outcomes with a relatively higher quality (at least *Moderate* GRADE), and two of them were *High* GRADE. IV TXA in spine surgery (WMD -214.68ml, 95% CI, -281.10 to -148.27) and orthognathic surgery (WMD -153.97ml, 95% CI, -166.52 to -141.41) could reduce perioperative bleeding and intraoperative bleeding, respectively (Zhao et al., 2019; Chen et al., 2021). Besides, Huang et al. (2015) reported that antifibrinolytic drugs could significantly reduce blood loss in THA (WMD -389.14, 95% CI, -483.95 to -295.23). In conclusion, TXA could effectively reduce blood loss and play a more significant role in major orthopedic surgery, including THA, TKA, and spine surgery.

As for subgroups, antifibrinolytic drugs could lead to a reduction of both intraoperative and postoperative blood loss (see Supplementary Appendix S12). A few studies that discussed SMD blood loss are shown in Supplementary Appendix S12d. IV TXA could significantly reduce intraoperative blood loss in endoscopic sinus surgery (SMD -0.66, 95% CI, –0.86 to -0.46) (Kim et al., 2019). Du and Feng concluded that IV or topical TXA could reduce intraoperative blood loss in spinal fusion surgery (SMD -0.32, 95% CI, -0.58 to -0.06) (Du and Feng, 2018).

### 3.3 Transfusion Rate and Adverse Events

Blood transfusion is an emergency treatment and could reflect the critical situation of the patient. In all, 14 surgeries provided outcomes with a relatively higher quality, and 16 outcomes were rated as *High* (see Tables 2, 3). According to 42 RCTs, Huang et al. (2014) concluded that TXA would reduce the transfusion rate in major orthopedic surgery by 49% (RR 0.51, 95% CI, 0.46–0.56). Besides, data from 31 RCTs demonstrated that IV or topical TXA in cardiac surgery could reduce the transfusion rate by 29% (RR 0.71, 95% CI, 0.65–0.78) (Guo et al., 2019). We also summarized incidences of adverse events containing seizure (a neurologic event), death, and vascular adverse events (see Table 4). Vascular adverse events included deep vein thrombosis (DVT), stroke, myocardial infarction (MI), and pulmonary embolism (PE). Five outcomes were rated as *High*. Zhang S. et al. (2019) reported that IV TXA could effectively reduce the rate of all adverse events in calcaneal fracture surgery (RR 0.26, 95% CI, 0.15–0.42). Li et al. indicated that IV antifibrinolytic drugs decreased the risk of DVT in spine surgery by 64%. Besides, TXA decreased the mortality of upper gastrointestinal bleeding by 41% (Twum-Barimah et al., 2020). In contrast, Guo et al. (2019) displayed that high-dose TXA increased the risk of seizure by nearly five times in cardiac surgery, and Zhang S. et al. (2019) also demonstrated an increased incidence of seizure by more than six times. Therefore, we concluded that TXA could reduce the transfusion rate and that TXA is also a protective factor from vascular adverse events and death, but it is a risk factor for seizures.

**TABLE 2:** Outcomes of the transfusion rate with high GRADE.

**Author year**	**Surgery**	**Agent**	**Characteristic**	**Subgroup**	**RCTs**	**Patients**		**95% CI Estimate**
Zhao et al. (2019)	OS	10–20 mg/kg	IV	10 mg/kg	3	290	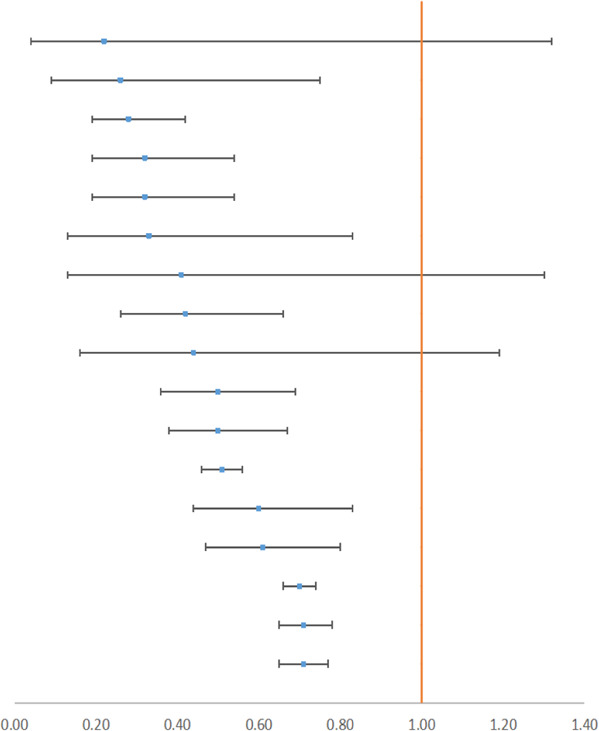	OR 0.22 [0.04,1.32]
Yao et al. (2019)	PAO and HTO	Inconsistent	IV/topical	—	6	665		RR 0.26 [0.09,0.75]
Chen et al. (2014)	TKA	0.5–3 g	topical	Any dosage	6	647		RR 0.28 [0.19,0.42]
Zhang et al. (2019a)	CABG	10–20 mg/kg	IV	Off-pump	3	206		RR 0.32 [0.19,0.54]
Zhao et al. (2019)	OS	10–20 mg/kg	IV	Any dosage	7	463		OR 0.33 [0.13, 0.83]
Zhao et al. (2019)	OS	10–20 mg/kg	IV	20 mg/kg	3	133		OR 0.41 [0.13,1.30]
Huang et al. (2015)	THA	Inconsistent	IV/topical	—	14	816		RR 0.42 [0.26,0.66]
Du and Feng. (2018)	Spinal fusion	10–30 mg/kg	IV/topical	—	3	231		RR 0.44 [0.16,1.19]
Zhou et al. (2019)	IFS	10–15 mg/kg	IV	—	8	836		OR 0.50 [0.36,0.69]
Guo et al. (2019)	Cardiac surgery	Inconsistent	IV/topical	Low dosage	7	265		RR 0.50 [0.38,0.67]
Huang et al. (2014)	MOS	Inconsistent	IV/topical/oral	—	42	2,649		RR 0.51 [0.46,0.56]
Guo et al. (2019)	Cardiac surgery	Inconsistent	IV/topical	Off-pump	7	660		RR 0.60 [0.44,0.83]
Longo et al. (2018)	Prostate surgery	10–15 mg/kg	IV/oral/local spray	Any dosage	7	718		RR 0.61 [0.47,0.80]
Guo et al. (2019)	Cardiac surgery	Inconsistent	IV	—	28	8,053		RR 0.70 [0.66,0.74]
Guo et al. (2019)	Cardiac surgery	Inconsistent	total	—	31	8,925		RR 0.71 [0.65,0.78]
Guo et al. (2019)	Cardiac surgery	Inconsistent	IV/topical	High dosage	12	2043		RR 0.71 [0.65,0.77]

OS, orthognathic surgery; PAO, periacetabular osteotomy; HTO, high tibial osteotomy; CABG, coronary artery bypass grafting; MOS, major orthopedic surgery (TKA, THA, and spine surgery); IFS, intertrochanteric fracture surgery.

**TABLE 3:** Outcomes of the transfusion rate with moderate GRADE.

Author year	Surgery	Agent	Characteristic	Subgroup	RCTs	Patients		95% CI Estimate
Chen et al. (2014)	TKA	0.5–3 g	Topical	Total dose<1.5 g	2	168	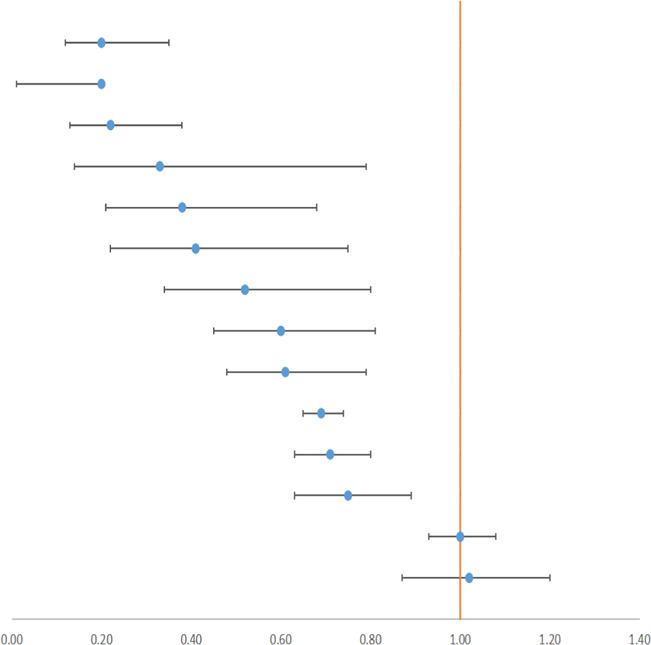	RR 0.20 [0.12,0.35]
Yao et al. (2019) ^a^	HTO	Inconsistent	IV/topical	—	3	332		RR 0.20 [0.01,4.10]
Chen et al. (2014)	TKA	0.5–3 g	Topical	Clamped drain<2 h	3	250		RR 0.22 [0.13,0.38]
Chen et al. (2014)	TKA	0.5–3 g	Topical	Clamped drain ≥2 h	2	128		RR 0.33 [0.14,0.79]
Wu et al. (2017)	TKA	10 mg/kg	IV	—	5	398		RR 0.38 [0.21,0.68]
Chen et al. (2014)	TKA	0.5–3 g	Topical	Total dose ≥1.5 g	4	344		RR 0.41 [0.22,0.75]
Montroy et al. (2017)	Cancer	Inconsistent	IV/topical	—	7	955		RR 0.52 [0.34,0.80]
Longo et al. (2018)	Prostate surgery	10–15 mg/kg	IV/oral/local spray	—	3	462		RR 0.60 [0.45,0.81]
Chen et al. (2021)	Spine surgery	5–30 mg/kg	IV	—	5	471		RR 0.61 [0.48,0.79]
Guo et al. (2019)	Cardiac surgery	Inconsistent	IV/topical	Low dosage	5	496		RR 0.69 [0.65,0.74]
Guo et al. (2019)	Cardiac surgery	Inconsistent	IV/topical	On-pump	23	3,299		RR 0.71 [0.63,0.80]
Li et al. (2020)	Spine surgery	4–100 mg/kg	IV	—	12	815		RR 0.75 [0.63,0.89]
Twum-Barimah et al. (2020)	UGIB	Inconsistent	IV	—	8	1763		RR 1.00 [0.93,1.08]
Guo et al. (2019)	Cardiac surgery	Inconsistent	topical	—	4	797		RR 1.02 [0.87,1.20]

HTO, high tibial osteotomy; UGIB, upper gastrointestinal bleeding; EACA , epsilon-aminocaproic acid; AP, aprotinin.

a Considering the overall presentation, we only show half the outcome of the study by Yao et al. (2019).

b We included these complex data because we failed to extract the solo data of TXA from the study.

**TABLE 4:** Outcomes of adverse events with high or moderate GRADE.

**Author year**	**Surgery**	**Agent**	**Characteristic**	**Outcome**	**Subgroup**	**RCTs**	**Patients**		**95% CI Estimate**	**GRADE**
Zhang et al. (2019b)	Calcaneal fracture	10–20 mg/kg	IV	Any event	—	6	389	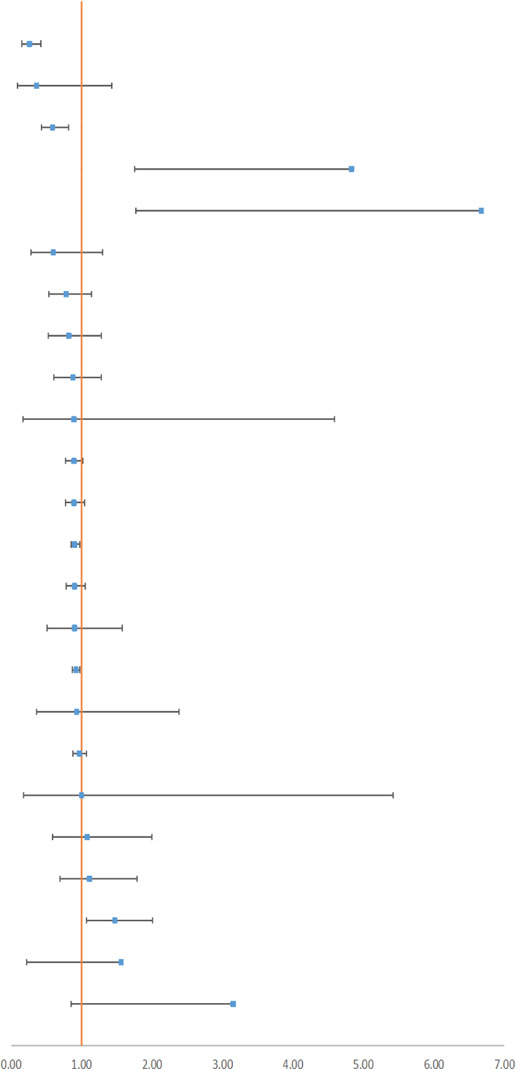	RR 0.26 [0.15,0.42]	High
Li et al. (2020)	Spine surgery	4–100 mg/kg	IV	DVT	—	17	1191		RR 0.36 [0.09,1.43]	High
Twum-Barimah et al. (2020)	UGIB	Inconsistent	IV/topical/oral	Mortality	—	10	2013		RR 0.59 [0.43,0.82]	High
Guo et al. (2019) ^a^	Cardiac surgery	Inconsistent	IV/topical	Seizure	High dosage	5	5,807		RR 4.83 [1.75,13.33]	High
Zhang (2019) ^a^	CABG	10–20 mg/kg	IV	Seizure	—	4	4,911		RR 6.67 [1.77,25.20]	High
Montroy (2017)	Cancer	Inconsistent	IV/topical	DVT	—	9	1075		OR 0.60 [0.28, 1.30]	Moderate
Guo et al. (2019)	Cardiac surgery	Inconsistent	IV/topical	Mortality	—	29	8,907		RR 0.78 [0.54,1.14]	Moderate
Zhang et al. (2019b)	CABG	10–20 mg/kg	IV	Mortality	On-/off-pump	17	6,259		RR 0.82 [0.53,1.28]	Moderate
Guo et al. (2019)	Cardiac surgery	Inconsistent	IV/topical	Stroke	—	32	9,257		RR 0.88 [0.61,1.28]	Moderate
Twum-Barimah et al. (2020)	UGIB	Inconsistent	IV	Vascular event	—	6	1041		RR 0.89 [0.17,4.59]	Moderate
Hu et al. (2019)	Cerebral hemorrhage	Inconsistent	IV	Mortality	All-follow-up	20	10,253		OR 0.89 [0.77, 1.02]	Moderate
Guo et al. (2019)	Cardiac surgery	Inconsistent	Topical/IV	MI	—	32	8,688		RR 0.89 [0.77,1.04]	Moderate
Ker (2015)	Acute trauma	Inconsistent	IV	Mortality	—	2	20,367		RR 0.90 [0.85,0.97]	Moderate
Zhang et al. (2019a)	CABG	10–20 mg/kg	IV	MI	On-/off-pump	23	6,714		RR 0.90 [0.78,1.05]	Moderate
Zhang et al. (2019b)	CABG	10–20 mg/kg	IV	MI	On-pump	13	1286		RR 0.90 [0.51,1.58]	Moderate
July and Pranata. (2020)	Brain trauma	1g	IV	Mortality	—	5	30,262		RR 0.92 [0.87,0.97]	Moderate
Zhang et al. (2019a)	CABG	10–20 mg/kg	IV	Mortality	On-pump	12	1302		RR 0.93 [0.36,2.38]	Moderate
Khair et al. (2019)	Cardiac surgery	Inconsistent	IV	Any event	—	44	9,896		RR 0.97 [0.88,1.07]	Moderate
Wu et al. (2017)	TKA	IV 10 mg/kg	IV	DVT	—	6	394		RR 1.00 [0.18,5.42]	Moderate
Guo et al. (2019)	Cardiac surgery	Inconsistent	IV/topical	PE	—	18	6,587		RR 1.08 [0.59,2.00]	Moderate
Huang et al. (2014)	MOS	Inconsistent	IV/topical/oral	DVT	—	44	2,689		RR 1.11 [0.69,1.79]	Moderate
Hu et al. (2019)	Cerebral hemorrhage	Inconsistent	IV	Vascular event	—	3	2,904		OR 1.47 [1.07,2.01]	Moderate
Zhang et al. (2019b) ^a^	CABG	Inconsistent	IV	MI	Off-pump	9	798		RR 1.56 [0.22,11.23]	Moderate
Tsai et al. (2020) ^a^	Hemoptysis	500 mg-1 g	IV	Any event	—	2	70		OR 3.15 [0.85,11.63]	Moderate

UGIB, upper gastrointestinal bleeding; CABG, coronary artery bypass grafting; MOS, major orthopedic surgery (TKA, THA, and spine surgery); DVT, deep venous thrombosis; MI, myocardial infarction; PE, pulmonary embolism.

a Considering the overall presentation, we only show half the outcome of the studies by Guo et al. (2019), Zhang et al. (2019a), and Tsai et al. (2020).

b We included these complex data because we failed to extract the solo data of TXA from the study.

### 3.4 Other Outcomes

We also evaluated outcomes of reoperation rate, operative time, length of hospital stay, and hemoglobin (Hb). Re-operation was the additional surgical intervention due to poor postoperative recovery. The reduction in the postoperative reoperation rate could avoid unnecessary pain and the economic burden on patients. As shown in Supplementary Appendix S13a, Zhang Y. et al. (2019) reported that IV TXA led to a 54% decrease in the reoperation rate in coronary artery bypass grafting (CABG) (RR 0.46, 95% CI, 0.31, 0.68). Similarly, Guo et al. (2019) showed that IV or topical TXA averts 38% of reoperation in cardiac surgery.

Besides, the operative time and length of hospital stay were also significantly shortened with TXA treatment (see Supplementary Appendix S13b,c). Zhao et al. demonstrated the decreasing operation time of orthognathic surgery with IV TXA (WMD -16.18, 95% CI, -19.60 to -12.75) (Zhao et al., 2019). Tsai et al. also reported that IV TXA could shorten the length of hospital stay for hemoptysis patients by 1.62 days (Tsai et al., 2020). These outcomes demonstrated the hemostatic effect of TXA, with faster postoperative recovery.

Furthermore, postoperative Hb outcomes have been displayed in Supplementary Appendix S14a,b. Chen et al. determined that the IV TXA group gained a higher Hb level after spine surgery (SMD 0.20, 95% CI, 0.02–0.38) (Chen et al., 2021). Yao et al. (2019) and Guo et al. (2018) also suggested that patients receiving TXA showed fewer Hb decreases. However, in terms of the coagulation function, there is no significant difference in partial thromboplastin time and prothrombin time (see Supplementary Appendix S14c).

### 3.5 Recommended Application

We summarized the recommended application of TXA in different types of adult surgery after the overall data analysis. IV-combined topical TXA (1–1.5 g IV + 1–3 g topical) was recommended for joint replacement surgery, and 10–15 mg/kg IV TXA was recommended in spinal surgery. 10 mg/kg IV TXA was recommended for cardiac surgery, and 2 g IV TXA was recommended in cerebral hemorrhage. Other detailed data are presented in Table 5.

**TABLE 5:** Recommended dosage of TXA in different types of adult surgery.

Types of surgery	Dosage	Administration	References
Major orthopedic surgery (joint replacement surgery)	1–1.5 g IV +		
	1–3 g topical	IV combined topical	Sun et al. (2019) ^[40]^
Cardiac surgery	10 mg/kg	IV	Zhang et al. (2019a) ^[29]^
Cerebral hemorrhage	1g	IV	July and Pranata (2020) ^[45]^
Spinal surgery	10–15 mg/kg	IV	Chen et al. (2021) ^[22]^
Intertrochanteric fracture	4 g	IV	Zhou et al. (2019) ^[43]^
Craniosynostosis surgery	10 or 50 mg/kg	IV	Lu et al. (2019) ^[46]^
Nasal surgery	1 g	Oral	De Vasconcellos et al. (2018) ^[49]^
Cesarean section	10 or 15 mg/kg	IV	Wang et al. (2019) ^[52]^
Tonsillectomy^a^	10 mg/kg	IV	Chan et al. (2013) ^[55]^
Orthognathic surgery	10 mg/kg	IV	Zhao et al. (2019) ^[23]^
Minor oral surgery	4.8%^b^	Gargle	De Vasconcellos et al. (2017) ^[56]^
Hemoptysis	500 mg	Nebulization	Tsai et al. (2020) ^[32]^
Prostatectomy	500 mg or 1 g	IV	Longo et al. (2018) ^[58]^

a The corresponding meta-analysis includes three RCTs and four case–control studies.

b 10 ml TXA, gargle for 2 min.

## 4 Discussion

### 4.1 Principle Findings

We analyzed data from 47 meta-analyses and demonstrated that TXA effectively reduced blood loss and the transfusion rate in various surgeries. TXA resulted in a higher Hb level, shorter operative time and length of hospital stay, and a decreased rate of reoperation. Besides, TXA did not increase the risk of death and vascular adverse events, but it was a risk factor for seizure in cardiac surgery. Therefore, we believe that TXA is a safe choice for hemostasis in major surgeries, in particular with a high risk of allogeneic blood transfusion. We also recommended the dosage of TXA in different types of adult surgery.

### 4.2 Supplemental Outcome Measures of Dosage and Administration

#### 4.2.1 Major Orthopedic Surgery

A high dose of IV TXA and combined IV and topical TXA are recommended in joint replacement surgery. In terms of dosage, Chen et al. suggested that a high dose of IV TXA (30 mg/kg) was more effective than a low dose (15–20 mg/kg) in reducing blood loss without increasing the risk of DVT (Chen et al., 2014). However, the heterogeneity may come from selection biases: Sukeik et al. (2020), Chen et al., and Wu et al. (2017) mentioned that patients with a cardiovascular disease history and renal insufficiency were excluded, but Guo et al., Kirsch et al. (2017), and Li et al. (2020) did not. As for administration routes, detailed outcomes are displayed in Supplementary Appendix S15. Li’s research suggested topical usage: topical TXA reduced 33.38 ml more than IV TXA did on total blood loss (WMD 33.38, 95% CI, 19.24–47.51). However, Li et al. (2020) demonstrated insignificant differences in the blood transfusion rate and adverse effects. In addition, similar outcomes were found in oral and IV TXA for all aspects. However, it should be noted that combined IV and topical TXA would be better than a single route. Sun’s research showed that the combined group decreased 198.07 ml total blood loss (WMD -198.07, 95% CI, -307.67 to -88.46) and the blood transfusion rate was 60% less than that of the single route (RR 0.40, 95% CI, 0.24–0.68). Zhang H. et al. (2017) and Zhang H. et al. (2017) also reported similar outcomes preferring combined IV and topical TXA (see Supplementary Appendix S15b) (Zhang H. et al., 2017; Zhang XQ. et al., 2017).

#### 4.2.2 Other Types of Orthopedic Surgery

Other types of orthopedic surgery were also included. As for spinal surgery, a starting dose of 10–15 mg/kg and a maintenance dose of 1.0–2.0 mg/(kg·h) were optimal in Chen’s study (Chen et al., 2021). Du and Feng (2018) demonstrated that intraoperative blood loss and postoperative blood loss were significantly reduced in adolescents with idiopathic scoliosis, but this could not be confirmed in lumbar pedicle subtraction osteotomy surgery. Besides, Zhou et al. (2019) concluded that 4 g TXA leads to the least amount of blood loss in femoral intertrochanteric fracture (WMD -570.8, 95% CI, -1071.04 to -70.56), which demonstrated that a larger dose corresponds to better effectiveness when under the premise of safety. Moreover, Zhang S. et al. (2019) reported that TXA could significantly reduce the incidence of postoperative incision complications in calcaneal fracture (RR 0.26, 95% CI, 0.15–0.42), which is common but inevitable.

#### 4.2.3 Cerebral Surgery

Three meta-analyses of cerebral surgery were included. Hu et al. (2019) found that early TXA treatment (within 3 h) after cerebral hemorrhage was effective in reducing the incidence of deaths and hematoma (as well as the volume of hematoma) caused by cerebral trauma. However, delayed TXA treatment (beyond 3 h) would not benefit the patients. Therefore, they suggested that patients with acute cerebral hemorrhage should use TXA as soon as possible (<3 h). Similarly, July and Pranata (2020) also agreed that patients with acute brain injury <3 h or a mild to moderate Glasgow Coma Scale score would need early TXA treatment. In addition, the optimal dose of TXA remained controversial (50 mg/kg or 10 mg/kg) in craniosynostosis surgery, but Lu et al. (2019) demonstrated that both doses could significantly reduce blood transfusion.

#### 4.2.4 Cardiac Surgery

In terms of cardiac surgery, we included three studies and summarized the following suggestions: low-dose IV TXA was the best choice, and it was unnecessary to exclude patients with renal impairment, coagulation dysfunction, or a history of thromboembolism. All these included studies indicated that high-dose TXA was significantly associated with the incidence of seizure (Joseph et al., 2018; Zhang S. et al., 2019; Khair et al., 2019). Guo et al. (2019) demonstrated that low-dose TXA (10 mg/kg) was sufficient to reduce blood transfusion requirements without increasing the risk of seizure, although high-dose TXA displayed less blood loss. In addition, Guo et al. (2019) found that topical TXA (whether combined with IV TXA or not) was not enough to reduce bleeding or the blood transfusion rate, and therefore, IV TXA was recommended in cardiac surgery.

#### 4.2.5 Nasal Surgery

According to the four included articles, different routes of administration had a similar hemostatic effect, and both oral and IV TXA were reliable in short-term surgical results and for thromboembolic complications (de Vasconcellos et al., 2018; Kim et al., 2019; Kang and Hwang, 2020). Due to the narrow scope of surgery in the nasal cavity, slight bleeding could distort the vision of the endoscope. TXA reduced intraoperative blood loss and improved the quality of the surgical field in endoscopic sinus surgery, thereby increasing the surgeon’s satisfaction and the success rate of the operation (Kim et al., 2019; Kang and Hwang, 2020). However, de Vasconcellos et al. (2018) reported that giving oral TXA (1 g) (WMD -61.70, 95% CI, –83.02 to –40.39) 2 h before rhinoplasty surgery reduced more intraoperative bleeding than IV TXA (10 mg/kg) (WMD –23.88, 95% CI, –45.19 to –2.58).

#### 4.2.6 Obstetrics and Gynecological Surgery

TXA has been used generally in cesarean section, vaginal delivery, and heavy menstrual bleeding (>80 ml blood loss per menstrual cycle). Wang’s study demonstrated that different doses (10 or 15 mg/kg) of TXA had similar effects in reducing blood loss and the transfusion rate in cesarean section (Wang et al., 2019). Wang also found that TXA significantly reduced the application of uterine tension agents (oxytocin and methylergometrine). In addition, according to Bryant-Smith’s study, TXA showed better efficacy than nonsteroidal anti-inflammatory drugs and progestogens (Bryant-Smith et al., 2018).

Besides, a study mainly involving orthopedics, obstetrics and gynecology, and maxillofacial and oral surgery showed the most common single preoperative dose is 15 mg/kg (Heyns et al., 2021).

#### 4.2.7 Other Types of Surgery

We included a few operations that were difficult to be classified. Chan’s study indicated that 10 mg/kg IV TXA significantly reduced the average blood loss (−32.72 ml) and average bleeding time (−3.6 h) of tonsillectomy patients (Chan et al., 2013). Tonsils are rich in plasminogen, which may explain why anti-plasminogen TXA works well (Chan et al., 2013). In orthognathic surgery, Zhao et al. (2019) recommended 10 mg/kg IV TXA as the most effective dose for intraoperative bleeding reduction. Besides, de Vasconcellos et al. (2017) reported that flushing the surgical site with TXA (4.8%) and then gargling within 1 week after minor oral surgery could reduce the risk of bleeding in anticoagulant patients (RR 0.13, 95% CI, 0.03–0.45). However, no difference was found between TXA and the standard care group (gelatin sponge and sutures or dry gauze compression) (Engelen et al., 2018). As for prostatectomy, about 8% of patients have a blood loss of more than 2000 ml. According to Longo et al., 500 mg or 1 g TXA significantly reduced blood loss (SMD −1.93, 95% CI, −2.81 to −1.05) and the blood transfusion rate (RR 0.61, 95% CI, 0.47–0.80) (Longo et al., 2018). In addition, patients with hemoptysis were recommended for TXA (500 mg) treatment by Tsai et al. (2020). Nebulization of TXA was also helpful, which directly acts on the bleeding site and provides a beneficial hemostatic effect.

### 4.3 Strength and Weakness

#### 4.3.1 Strength of TXA

As a lysine analogue, TXA inhibits the activation of plasminogen by blocking the lysine binding site, which reduces the hydrolysis of fibrinogen and stabilizes the blood clot (Hunt, 2015). In addition to hemostasis, TXA has certain effects in other aspects. In rhinoplasty surgery, TXA reduces eyelid edema in the first week after surgery by decreasing interleukin-6 and acute-phase proteins (de Vasconcellos et al., 2018). Besides, TXA has an anti-inflammatory effect, where early use of TXA (<60min) can reduce endothelial cell apoptosis and necrosis (Diebel et al., 2017). TXA seems to reduce the incidence of incision complications, which could be seen in calcaneal fracture surgery (Zhang Y. et al., 2019).

TXA provides surgeons with new ideas for hemostasis in surgical operations and can also save blood transfusion. Attributed to its hemostatic and blood-saving effects, TXA is regarded as an excellent alternative therapy. Global demand and availability of blood products were studied by Roberts et al., and they found that 119 countries (61%) did not have enough blood supply to meet their needs. The gap between demand and supply was significant in many developing countries. They showed that the WHO target of 10–20 donations per 1,000 people was underestimated for many countries (Roberts et al., 2019). In addition, TXA is cheap enough to reduce the financial burden on patients. Kirsch et al. (2017) reported that $8,000 was saved per 100 patients undertaking TKA or THA treated with TXA. Besides, Lu et al. (2019) concluded that the cost of blood transfusion in open craniosynostosis surgery was estimated to be more than 100 pounds higher if TXA was not used. Moreover, with a half-life of 3 h, the strongest hemostatic effect appears in the first 24 h so that quick effect achieves the purpose of less medication and cost (Kirsch et al., 2017; Lu et al., 2019; Yao et al., 2019).

#### 4.3.2 Adverse Effect of TXA

According to the included studies, TXA is proven as a protective factor against vascular adverse events. Furthermore, although pregnant women are in a state of hypercoagulability, no increased risk of DVT is revealed in vaginal delivery and cesarean section (Wang et al., 2019; Xia et al., 2020). In cardiac surgery, Khair et al. (2019) demonstrated that TXA could also be applied in patients with renal impairment, coagulation dysfunction, or a history of thromboembolism. Besides, a recent meta-analysis of 216 studies showed that regardless of dosage, IV TXA was not associated with an increased risk of any thromboembolic events (Taeuber et al., 2021).

Potential adverse events have limited the clinical application of TXA: high-dose TXA is a risk factor for seizure in cardiac surgery, which was shown in three studies (Zhang Y. et al., 2019; Guo et al., 2019; Khair et al., 2019). In particular, Guo’s subgroup analysis demonstrated a high risk of seizure in the high-dose TXA group (RR 4.83, 95% CI, 1.75 to 13.33) (Guo et al., 2019). Zufferey declared that low-dose TXA increased the risk of seizures by a factor of 1.2; meanwhile, the risk factor of high-dose TXA is 2 (Zufferey et al., 2021). A metaregression by Murao showed an increased risk of seizures with an increased dose of TXA (*p* = 0.011) (Murao et al., 2021). Another study also showed that the incidence of postoperative seizures in cardiac surgery increased from 0.5–1.0% to 6.4–7.3% after the application of high doses of TXA (Lecker et al., 2021). Stephan et al. had clearly demonstrated that TXA enhanced neuronal excitation by antagonizing inhibitory GABAergic neurotransmission *via* postsynaptic mechanisms (Kratzer et al., 2014). TXA, as a competitive antagonist, is structurally similar to glycine, which prevents the activation of glycine receptors and leads to the development of seizure (Lecker et al., 2012). Besides, isoflurane and the less effective propofol had been found useful in treating TXA-induced seizures, which guarantee the safety of high-dose TXA (Lecker et al., 2012).

#### 4.3.3 Umbrella Review

This is the first umbrella review on the efficacy and safety of the application of TXA in surgeries. An umbrella review is a targeted compilation of evidence for various clinical-related questions on all health outcomes associated with a particular exposure. It is an ideal way of presenting a wide picture of the evidence related to an interesting question, and a higher methodological quality results in a more reliable conclusion. Precisely, in order to screen qualified results from these research studies and produce guidelines for clinical application of antifibrinolytic agents, we summarized the evidence produced to date and took a judicial, critical approach to the quality of these systematic reviews and meta-analyses. After the evaluation of AMSTAR, most of the included studies were of *High* quality. In addition, the studies we included are relatively updated, with 40 studies published after 2016.

However, limitations also exist: TXA may also be applied to other types of surgery, but they were not included in our umbrella review without related meta-analysis. Besides, the studies not written in English or Chinese are excluded, which provides potential bias. Furthermore, for the same outcome (such as blood loss), each meta-analysis has a different effect size, which may be a potential source of heterogeneity. However, we have tried our best to perform separate comparative studies without compromising the conclusion.

## 5 Conclusion

Our study demonstrates that TXA has a general hemostatic effect with very few adverse events, which indicates TXA is the recommended medication to prevent excessive bleeding and reduce the blood transfusion rate. We recommended different dosages of TXA for different types of adult surgery. However, we could not recommend a unified dosage for different surgeries due to the heterogeneity of the experimental design.

## Abbreviations

RCTs, randomized controlled trials; TXA, tranexamic acid; Hb, Hemoglobin; TKA, total knee arthroplasty; THA, total hip arthroplasty; PRISMA, Preferred Reporting Items for Systematic Reviews and Meta-Analyses; WMD, weighted mean difference; SMD, standard mean difference; CI, confidence interval; OR, odds ratio; RR, relative risk; RD, risk difference; AMSTAR, assessing the methodological quality of systematic reviews; GRADE, Grading of Recommendations, Assessment, Development and Evaluation; CCA, corrected covered area; DVT, deep vein thrombosis; MI, myocardial infarction; PE, pulmonary embolism; CABG, coronary artery bypass grafting.
