# Supplementary material for: Does Tranexamic Acid Reduce the Blood Loss in Various Surgeries? An Umbrella Review of State-of-the-Art Meta-Analysis
Source: Front Pharmacol. 2022 May 19;13:887386. doi: 10.3389/fphar.2022.887386 (PMC9160460; doi:10.3389/fphar.2022.887386)
Supplement: Supplementary file 1 [file DataSheet1.docx]

**Appendix**

Appendix 1. Medline Search Strategy

Appendix 2. JBI Data Extraction Form for Review for Systematic Reviews and Research Syntheses

Appendix 3. AMSTAR 2

Appendix 4. GRADE

Appendix 5. Citation matrices for reviews with overlapping associations

Appendix 6. Characteristics of studies included in systematic review

Appendix 7. List of excluded studies and reasons for their exclusion

Appendix 8. Tabular presentation of outcomes in included Meta-analysis.

Appendix 9. Tabular presentation of discussion in included Meta-analysis.

Appendix 10. AMSTAR 2 quality appraisal scores of included meta-analysis

Appendix 11. Details of GRADE

Appendix 12. Outcomes of blood loss

Appendix 13. Operative time and length of stay

Appendix 14. Hb and coagulation function

Appendix 15. Outcomes of comparison between different routes of TXA in major orthopedic surgery

## Appendix 1. Medline Search Strategy

| Concept | Strategy | Result^*^ |
| --- | --- | --- |
| 1 | exp tranexamic acid/ | 4128 |
| 2 | (Tranexamic acid* or TXA*). ti,ab. | 6279 |
| 3 | 1 OR 2 | 7079 |
| 4 | (Systematic review or meta-analysis). pt. | 237796 |
| 5 | 3 AND 4 | 410 |
| 6 | exp animals/ not humans.sh | 4885127 |
| 7 | 5 NOT 6 | 410 |

^*^an updated search on Feb 27^th^ ,2022

(Tranexamic acid[MeSH Terms] OR Tranexamic acid[Title/Abstract] OR TXA[Title/Abstract]) AND (systematic review[Publication Type] OR meta analysis[Publication Type]) NOT ((animals[MeSH Terms]) NOT (humans[MeSH Terms]))

## Appendix 2. JBI Data Extraction Form for Review for Systematic Reviews and Research Syntheses^*^

| **Study Details** | |
| --- | --- |
| Author/year |  |
| Objectives |  |
| Participants (characteristics/ total number) |  |
| Setting/context |  |
| Description of Interventions/  phenomena of interest |  |
| **Search Details** | |
| Sources searched |  |
| Range (years) of included studies |  |
| Number of studies included |  |
| Types of studies included |  |
| Country of origin of included studies |  |
| **Appraisal** | |
| Appraisal instruments used |  |
| Appraisal rating |  |
| **Analysis** | |
| Method of analysis |  |
| Outcome assessed |  |
| Results/Findings |  |
| Significance/direction |  |
| Heterogeneity |  |
| Comments |  |

^*^Reference by: E. Aromataris, R.S. Fernandez, C. Godfrey, C. Holly, H. Khalil, P. Tungpunkom, Methodology for JBI umbrella reviews, https://ro.uow.edu.au/smhpapers/3344, 2014 (assessed 15 Aug 2021).

## Appendix 3. AMSTAR 2

For Partial Yes (all the following):

▢ searched at least 2 databases

(relevant to research question)

▢ provides key word and/or search strategy

▢ justified publication restrictions (e.g. language)

For Yes, should also have (all the following):

▢ search the reference lists/bibliographies of included studies

▢ searched trial/study registries

▢ included/consulted content experts in the field

▢ where relevant, searched for grey literature

▢ conducted search within 24 mouths of completion of the review

For Yes:

As for partial yes, plus the protocol should be registered and should also have specified:

▢ a meta-analysis/synthesis plan, if appropriate, and

▢ a plan for investigating causes of heterogeneity

▢ justification for any deviations from the protocol

▢ Yes

▢ No

For Yes, the review should satisfy ONE of the following:

▢ Explanation for including only RCTs

▢ OR Explanation for including only NRSI

▢ OR Explanation for including both RCTs and NRSI

▢ Yes

▢ Partial Yes

▢ No

▢ Yes

▢ Partial Yes

▢ No

For Partial Yes:

The authors state that they had a written protocol or guide that included ALL the following:

▢ review question(s)

▢ a search strategy

▢ inclusion/exclusion

▢ a risk of bias assessment

For Yes: Optional(recommended)

▢ Population ▢ Timeframe for follow-up ▢ Yes

▢ Intervention ▢ No

▢ Comparator group

▢ Outcome

1. Did the research questions and inclusion criteria for the review include the components of PICO?

2. Did the report of the review contain an explicit statement that review methods were established prior to the conduct of the review and did the report justify any significant deviations from the protocol?

3. Did the review authors explain their selection of the study designs for inclusion in the review?

4. Did the review authors use a comprehensive literature search strategy?

For Yes, should also have ALL the following:

▢ described populations in detail

▢ described intervention in detail (including doses where relevant)

▢ described comparators in detail (including doses where relevant)

▢ described study’s setting

▢ timeframe for follow-up

5. Did the review authors perform study selection in duplicate?

6. Did the review authors perform data extraction in duplicate?

7. Did the review authors provide a list of excluded studies and justify the exclusions?

8. Did the review authors describe the included studies in adequate detail?

9. Did the review authors provide describe the included studies in adequate detail?

10.

▢ Yes

▢ Partial Yes

▢ No

▢ Includes only NRSI

▢ Yes

▢ Partial Yes

▢ No

▢ Yes

▢ Partial Yes

▢ No

For Yes, must also have assessed RoB from:

▢ allocation sequence that was not truly random, *and*

▢ selection of the reported result from among multiple measurements or analyses of a specified outcome

**RCTs**

For Partial Yes, must assessed RoB from:

▢ unconcealed allocation, *and*

▢ lack of blinding of patients and assessors when assessing outcomes (unnecessary for objective outcomes such as all-cause mortality)

For Partial Yes (ALL the following)

▢ described populations

▢ described interventions

▢ described comparators

▢ described outcomes

▢ described research designs

For Partial Yes: For Yes, must also have:

▢ provided a list of all potentially ▢ justified the exclusion from the review

relevant studies that were read in of each potentially relevant study

full-text but excluded from the review

▢ Yes

▢ No

For Yes, either ONE of the following:

▢ at least two reviewers achieved consensus on which data to extract from

included studies

▢ OR two reviewers extracted data from a sample of eligible studies and achieved

good agreement (at least 80 percent), with the remainder extracted by one reviewer

▢ Yes

▢ No

For Yes, either ONE of the following:

▢ at least two reviewers independently agreed on selection of eligible studies

and achieved consensus in which studies to include

▢ OR two reviewers selected a sample of eligible studies and achieved good

agreement (at least 80 percent), with the remainder selected by one reviewer

For Yes, must also have assessed RoB:

▢ methods used to ascertain exposures and outcomes, *and*

▢ selection of the reported result from among multiple measurements or analyses of a specified outcome

For Yes

▢ the authors justified combining the data in a meta-analysis

▢ AND they used an appropriate weighted technique to combine study re

results, adjusting for heterogeneity if present

▢ AND they statistically combined effect estimates from NRSI that were adjusted for confounding, rather than combining raw data, or justified combining raw data when adjusted effect estimates were not available

▢ AND they reported separate summary estimates for RCTs and NRSI separately when both were included in the review

|  |
| --- |

▢ described comparators

**NRSI**

▢ Yes

▢ No

▢ No meta-analysis conducted

For Yes:

▢ The authors justified combining the data in a meta-analysis

▢ AND they used an appropriate weighted technique to combine study results and adjusted for heterogeneity if present

▢ AND investigated the causes of any heterogeneity

**RCTs**

▢ Yes

▢ No

▢ No meta-analysis conducted

For Yes:

▢ included only low risk of bias RCTs

▢ OR, if the pooled estimate was based on RCTs and/or NRSI at variable RoB,

the authors performed analyses to investigate possible impact of RoB on

summary estimates of effect

10. Did the review authors report on the sources of funding for the studies?

11. If meta-analysis was performed did the review authors use appropriate methods for statistical combination of results?

12. If meta-analysis was performed, did the review authors assess the potential impact of RoB in individual studies on the results of the meta-analysis or other evidence synthesis?

9. Did the review authors provide describe the included studies in adequate detail?

10.

▢ Yes

▢ No

▢ No meta-analysis conducted

▢ Yes

▢ No

▢ Yes

▢ Partial Yes

▢ No

▢ Includes only RCTs

For Yes

▢ must have reported on the sources of funding for individual studies included in the review. Note: Reporting that the reviewers looked for this information but it was not reported by study authors also qualifies

|  |
| --- |

reported on the sources of funding for individual studies included in the review. Note: Reporting that the reviewers looked for this information but it was not reported by study authors also qualifies

|  |
| --- |

| reported on the sources of funding for individual studies included in the review. Note: Reporting that the reviewers looked for this information but it was not reported by study authors also qualifies |
| --- |

**NRSI**

For Partial Yes, must have assessed RoB:

▢ from confounding, *and*

▢ from selection bias

Note:

1.AMSTAR 2 is a critical appraisal tool for systematic reviews that include randomized or non-randomized studies of healthcare interventions, or both. The revised instrument AMSTAR 2 has 16 items in total (compared with 11 in the original AMSTAR); seven of these items (Q2, 4, 7, 9, 11, 13, 15) are considered critical that shortcomings in any of the critical domains could affect the overall validity. Revised AMSTAR has simpler response categories than the original AMSTAR, and includes a more comprehensive user guide, and has an overall rating based on weaknesses in critical domains.

2.Evaluating rules: A= Q2, 4, 7, 9, 11, 13, 15(critical) while B= Q1, 3, 5, 6, 8, 10, 12, 14, 16(not critical relatively). A study of High quality achieves zero or one No of B; A study of Moderate quality achieves more than two No of B; A study of Low quality achieves one No of A; A study of critically low achieves more than two No of A.

13. Did the review authors account for RoB in individual studies when interpreting/ discussing the results of the review?

14. Did the review authors provide a satisfactory explanation for, and discussion of, any heterogeneity observed in the results of the review?

10. Did the review authors report on the sources of funding for the studies?

15. If they performed quantitative synthesis did the review authors carry out an adequate investigation of publication bias (small study bias) and discuss its likely impact on the results of the review?

16. Did the review authors report any potential sources of conflict of interest, including any funding they received for conducting the review?

9. Did the review authors provide describe the included studies in adequate detail?

10.

▢ Yes

▢ No

For Yes:

▢ the authors reported no competing interests OR

▢ the authors described their funding sources and how they managed potential conflicts of interest

▢ Yes

▢ No

▢ No meta-analysis conducted

▢ Yes

▢ No

For Yes:

▢ performed graphical or statistical tests for publication bias and discussed the likelihood and magnitude of impact of publication bias



For Yes

▢ There was no significant heterogeneity in the results

▢ OR if heterogeneity was present the authors performed an investigation of sources of any heterogeneity in the results and discussed the impact of this on the results of the review

|  |
| --- |

| reported on the sources of funding for individual studies included in the review. Note: Reporting that the reviewers looked for this information but it was not reported by study authors also qualifies |
| --- |

▢ Yes

▢ No

For Yes:

▢ included only low risk of bias RCTs

▢ OR, if RCTs with moderate or high RoB, or NRSI were included the review provided a discussion of the likely impact of RoB on the results

## Appendix 4. GRADE

| Risk of  Bias | Inconsistency | Indirectness | Imprecision | Publication bias | Plausible Confounding | Magnitude of effect | Dose-response gradient |
| --- | --- | --- | --- | --- | --- | --- | --- |
|  |  |  |  |  |  |  |  |
|  |  |  |  |  |  |  |  |

GRADE = Grading of Recommendations, Assessment, Development and Evaluation

Each following question had 3 answers: “No serious risk”, “Serious risk” and “Very serious risk”.

Risk of Bias: “Serious risk” when sensitive analysis resulted in significant difference.

Inconsistency: “No serious risk” when I^2^≤50%; “Serious risk” when 50<I^2^≤75%; “Very serious risk” when I^2^>75%

Indirectness: Comprehensive consideration in combination with information.

Imprecision: “Serious risk” when P>0.05

Publication bias: “No serious risk” when Egger’s test P>0.05; “Serious risk” when 0.01<P≤0.05; “Very serious risk” when P≤0.01.

Plausible Confounding had 2 answers: “No” and “Yes”. It would be assessed by comprehensive consideration in combination with information.

Magnitude of effect had 3 answers: “No”, “Yes” and “Extremely” related to odds ratio (OR). “No” when 0.5<OR<2; “Yes” when 0.2<OR≤0.5 or 2≤OR<5; “Extremely” when OR≤0.2 or OR≥5.

Dose-response gradient had 2 answers: “No” and “Yes”. “Yes” when P value of dose-response related outcome ≤0.05.

There were 4 levels of quality: “High”, “Moderate”, “Low” and “Very low”. Evidence of RCTs were initially assessed as “High”. “Serious risk” would reduce 1 level of quality and “Very serious risk” would reduce 2 levels of quality. While “Yes” could promote 1 level of quality and “Extremely” could promote 2 level of quality.

## Appendix 5. Citation matrices for reviews with overlapping associations

Corrected covered area (CCA) was calculated as (N−r)/(rc−r), where N was the number of total publications included in evidence synthesis (including overlapping studies), r was the number of real publications (excluding overlapping studies), and c was the number of potential overlapping meta-analyses. Overlapping was categorized as high when CCA >10% and incorporation of results from reviews with overlapping associations would be progressed following the criteria below:

·When the overlapping reviews contained Cochrane and non-Cochrane review, Cochrane review was our priority selection.

·When both overlapping reviews were non-Cochrane reviews, the meta-analysis with higher AMSTAR score was selected in preference.

·When the overlap happened between studies with the same AMSTAR score, year of publication, number of participants and details of data were considered comprehensively to evaluate the proper meta-analysis.

A. Obstetrics and gynecology

| Systematic review | Wang 2019 | Franchini 2018 | Li 2017 | Topsoee 2016 | Simonazzi 2016 | Alam 2015 | Wang 2015 | Heesen 2014 | Peitsidis 2011 |
| --- | --- | --- | --- | --- | --- | --- | --- | --- | --- |
| Characteristics | caesarean section | caesarean section | caesarean section | caesarean section | caesarean section | caesarean section | caesarean section | caesarean section | caesarean section |
| Including study |  | | | | | | | | |
| Sujataet 2016 | √ | √ | √ |  |  |  |  |  |  |
| Ray 2016 | √ |  |  |  |  |  |  |  |  |
| Lakshmi 2016 | √ | √ |  |  |  |  |  |  |  |
| Maged 2015 | √ | √ | √ |  |  |  |  |  |  |
| Ahmed 2015 | √ | √ | √ | √ | √ | √ |  |  |  |
| Taj 2014 | √ |  | √ | √ |  |  |  |  |  |
| Singh 2014 | √ | √ | √ |  |  | √ |  |  |  |
| Ghosh 2014 | √ | √ | √ |  |  |  |  |  |  |
| Yehia 2014 |  | √ | √ |  |  | √ |  |  |  |
| Gobbur 2014 | √ | √ | √ |  |  | √ |  |  |  |
| Ramani 2014 | √ | √ | √ |  |  | √ |  |  |  |
| Xu 2013 | √ | √ | √ | √ | √ | √ | √ | √ |  |
| Halder 2013 |  |  | √ |  |  |  | √ |  |  |
| Shahid 2013 | √ | √ | √ | √ | √ | √ | √ | √ |  |
| Sentürk 2013 | √ | √ | √ |  | √ | √ | √ | √ |  |
| Goswami 2013 | √ | √ | √ | √ | √ | √ | √ | √ |  |
| Bhavana 2013 | √ |  |  | √ |  |  |  |  |  |
| Abdel-Aleem 2013 | √ | √ | √ | √ | √ | √ | √ |  |  |
| Poonia 2012 |  |  | √ |  |  |  |  |  |  |
| Rashmi 2012 |  |  | √ |  |  | √ |  |  |  |
| Tarabrin 2012 | √ |  |  | √ |  |  |  |  |  |
| Movafegh 2011 | √ | √ | √ | √ | √ | √ | √ | √ |  |
| Sharma 2011 |  |  |  | √ |  |  |  |  |  |
| Gungordu 2011 | √ | √ | √ | √ | √ | √ | √ | √ |  |
| Sekhavat 2009 | √ | √ | √ | √ |  | √ | √ |  | √ |
| Mayur 2007 |  |  | √ | √ |  | √ | √ |  | √ |
| Gai 2004 | √ | √ | √ | √ | √ | √ | √ |  | √ |
| Total | 21 | 18 | 22 | 14 | 9 | 16 | 11 | 6 | 3 |
| Grand total (N) | 120 | | | | | | | | |
| Real total (r) | 27 | | | | | | | | |
| No. of meta (c) | 9 | | | | | | | | |
| CCA | 43.1% | | | | | | | | |

| Systematic review | Xia 2020 | Li 2017 |
| --- | --- | --- |
| Characteristics | vaginal delivery | vaginal delivery |
| Including study |  | |
| Sentilhes 2018 | √ |  |
| Mirghafourvand 2015 | √ | √ |
| Gungorduk 2013 | √ | √ |
| Yang 2001 | √ | √ |
| Grand total (N) | 7 | |
| Real total (r) | 4 | |
| No. of meta (c) | 2 | |
| CCA | 75.0% | |

CCA =Corrected covered area. Calculation = CCA (%)=N-r/rc-r:

Where N=number of included publications (sum of checked boxes), r = number of rows (primary studies), c =number of columns (number of systematic reviews)

B. Rhinology

| Systematic review | Kang 2019 | Kim 2019 | Ping 2019 | Pundir 2013 |  | Kang 2019 | Kim 2019 |
| --- | --- | --- | --- | --- | --- | --- | --- |
| Characteristics | sinus surgery | sinus surgery | sinus surgery | sinus surgery |  | Topical | IV |
| Including study |  | | | |  |  | |
| Dongare 2018 |  | √ |  |  |  |  | √ |
| Baradaranfar 2017 | √ |  | √ |  |  | √ |  |
| Shale 2015 |  | √ |  |  |  |  | √ |
| Nuhi 2015 |  | √ | √ |  |  |  | √ |
| Shehata 2014 | √ |  |  |  |  | √ |  |
| Jahanshahi 2014 | √ |  | √ |  |  | √ |  |
| Langille 2013 |  | √ | √ | √ |  |  | √ |
| Alimian 2011 |  | √ | √ | √ |  |  | √ |
| Chhapola 2011 |  | √ |  | √ |  |  | √ |
| Moice 2010 |  | √ |  |  |  |  | √ |
| Athanasiadis 2007 |  |  | √ | √ |  |  |  |
| Jabalameli 2006 | √ |  | √ | √ |  | √ |  |
| Total | 4 | 7 | 7 | 5 |  | 4 | 7 |
| Grand total (N) | 23 | | | |  | 11 | |
| Real total (r) | 12 | | | |  | 11 | |
| No. of meta (c) | 4 | | | |  | 2 | |
| CCA | 30.6% | | | |  | 0% | |

| Systematic review | McGuire 2019 | Juliana 2018 |
| --- | --- | --- |
| Characteristics | Rhinoplasty | Rhinoplasty |
| Including study |  | |
| Mehdizadeh 2018 | √ | √ |
| Ghavimi 2017 | √ | √ |
| Eftekharian and Rajabzadeh 2016 | √ | √ |
| Sakallioğlu 2015 | √ | √ |
| Beikaei 2015 | √ | √ |
| Total | 5 | 5 |
| Grand total (N) | 10 | |
| Real total (r) | 5 | |
| No. of meta (c) | 2 | |
| CCA | 100.0% | |

IV=Intravenous.

CCA =Corrected covered area. Calculation = CCA (%)=N-r/rc-r:

Where N=number of included publications (sum of checked boxes), r = number of rows (primary studies), c =number of columns (number of systematic reviews)

C. Cardiology

| Systematic review | Zhang 2019 | Ma 2011 | Dai 2017 |
| --- | --- | --- | --- |
| Characteristics | CABG | CABG | CABG |
| Including study |  | | |
| Myles 2017 | √ |  | √ |
| Yanartas 2015 | √ |  |  |
| Hosseini 2014 |  |  | √ |
| Ghavidel 2014 | √ |  |  |
| Aoki 2013 |  |  | √ |
| Shi 2013 | √ |  |  |
| Esfandiari2013 | √ |  |  |
| Wang 2012 | √ |  | √ |
| Nejad 2012 | √ |  |  |
| Aoki 2012 |  |  | √ |
| Greiff 2012 | √ |  |  |
| Chakravarthy 2012 | √ |  | √ |
| Ahn 2012 | √ |  | √ |
| Hashemi 2011 | √ |  |  |
| Taghaddomi 2009 | √ | √ | √ |
| Mehr-Aein 2007 | √ | √ | √ |
| Maddali 2007 | √ |  |  |
| Murphy 2006 |  | √ | √ |
| Wei 2006 | √ | √ | √ |
| Santos 2006 | √ |  |  |
| Vanek 2005 | √ | √ | √ |
| Karski 2005 | √ |  |  |
| Casati 2004 | √ | √ | √ |
| Andreasen 2004 | √ |  |  |
| Pleym 2003 | √ |  |  |
| Jares 2003 | √ | √ | √ |
| Zabeeda 2002 | √ |  |  |
| Casati 2001 | √ | √ | √ |
| Hardy 1998 | √ |  |  |
| Landymore 1997 | √ |  |  |
| Brown 1997 | √ |  |  |
| Speekenbrink 1995 | √ |  |  |
| Total | 28 | 8 | 15 |
| Grand total (N) | 51 | | |
| Real total (r) | 31 | | |
| No. of meta (c) | 3 | | |
| CCA | 32.2% | | |

CABG=Coronary Artery Bypass Grafting Surgery

| Systematic review | K 2013 | Lin 2016 | Habbab 2019 | Guo 2019 | Khaie 2019 | Takagi 2017 | Faraoni 2012 |
| --- | --- | --- | --- | --- | --- | --- | --- |
| Characteristics | cardiac surgery | cardiac surgery | cardiac surgery | cardiac surgery | cardiac surgery | cardiac surgery | cardiac surgery |
| Including study |  | | | | | | |
| Mirmohammadsageghi 2018 |  |  |  | √ |  |  |  |
| Zhang Y 2018 |  |  |  | √ |  |  |  |
| Chaudhary 2018 |  |  |  | √ |  |  |  |
| Patel 2017 |  |  |  |  | √ |  |  |
| Myles 2017 |  |  |  | √ | √ | √ |  |
| Van A 2016 |  |  |  |  | √ |  |  |
| Kimenai 2016 |  |  |  | √ |  |  |  |
| Shah 2015 |  |  |  | √ |  |  |  |
| Muthialu 2015 |  |  |  |  | √ |  |  |
| Ali Shah 2015 |  |  | √ | √ |  |  |  |
| Hosseini 2014 |  |  | √ | √ |  |  |  |
| Alizadeh 2014 |  |  |  | √ | √ |  |  |
| Faraoni 2014 |  |  |  |  | √ | √ |  |
| Sharma 2014 |  | √ |  |  |  | √ |  |
| Falana 2014 |  |  |  |  |  | √ |  |
| Imtias 2014 |  |  |  |  | √ |  |  |
| Sigaut 2014 |  |  |  |  | √ |  |  |
| Du Y 2014 |  |  |  |  | √ |  |  |
| Gofton 2014 |  | √ |  |  |  |  |  |
| Esfandiari 2013 |  |  |  | √ | √ |  |  |
| Nouraei 2013 |  |  |  | √ |  |  |  |
| Chen T 2013 |  |  |  |  | √ |  |  |
| Makhija 2013 |  |  |  |  |  | √ |  |
| Koster 2013 |  | √ |  |  |  | √ |  |
| Shi 2013a |  |  |  | √ | √ |  |  |
| Shi 2013b |  |  |  |  | √ |  |  |
| Ahn 2012 |  |  |  | √ | √ |  |  |
| Bokesch 2012 |  |  |  |  | √ |  |  |
| Ghaffari 2012 |  |  |  |  | √ |  |  |
| Aoki 2012 |  |  | √ | √ |  |  |  |
| Greiff 2012 | √ |  |  | √ | √ |  |  |
| Mansouri 2012 |  |  |  |  | √ |  |  |
| Hassani 2012 |  |  |  | √ |  |  |  |
| Wang G 2012 |  |  |  | √ | √ |  |  |
| Kalavrouzitis 2012 |  | √ |  |  |  | √ |  |
| Montes 2012 |  | √ |  |  |  |  |  |
| Baric D 2011 |  |  |  | √ |  |  |  |
| Hashemi 2011 |  |  |  | √ | √ |  |  |
| Shimizu 2011 |  |  |  |  | √ |  | √ |
| Jimenez 2011 |  |  |  |  |  | √ |  |
| Keyl 2011 |  | √ |  |  |  | √ |  |
| Manji 2011 |  | √ |  |  |  | √ |  |
| Martin 2011 |  |  |  |  |  | √ |  |
| Murkin 2010 |  | √ |  |  |  |  |  |
| Bell 2010 |  |  |  |  |  | √ |  |
| Berman 2010 |  |  |  |  |  | √ |  |
| Sander 2010 |  |  |  |  |  | √ |  |
| Later 2009 |  |  |  | √ | √ |  |  |
| Taghaddomi 2009 |  |  |  | √ | √ |  |  |
| Fawzy 2009 |  |  | √ | √ |  |  |  |
| Dietrich 2008 |  |  |  |  | √ |  |  |
| Fergusson 2008 |  |  |  |  | √ |  |  |
| Martin 2008 |  | √ |  |  |  | √ |  |
| Baric 2007 |  |  | √ | √ |  |  |  |
| Maddali 2007 | √ |  |  | √ | √ |  |  |
| Mehr-Aein 2007 | √ |  |  | √ | √ |  |  |
| Jimenez 2007 | √ |  |  |  |  |  |  |
| Abul-Azm 2006 |  |  | √ | √ |  |  |  |
| Kuitunen 2006 | √ |  |  |  | √ |  |  |
| Santos 2006 |  |  |  |  |  | √ | √ |
| Moret 2006a | √ |  |  |  |  |  |  |
| Moret 2006b | √ |  |  |  |  |  |  |
| Murphy 2006 | √ |  |  | √ | √ |  |  |
| Wei M 2006 |  |  |  |  | √ |  |  |
| Bulutcu 2005 | √ |  |  |  | √ |  | √ |
| Diprose 2005 | √ |  |  | √ | √ |  |  |
| Karski 2005 | √ |  |  |  | √ |  |  |
| Kuitunen 2005 | √ |  |  |  | √ |  |  |
| Vanek 2005 |  |  |  |  | √ |  |  |
| Andreasen 2004 |  |  |  | √ | √ |  |  |
| Casati 2004a | √ |  |  | √ | √ |  |  |
| Casati 2004b | √ |  |  |  |  |  |  |
| Chauhan 2004a | √ |  |  |  | √ |  | √ |
| Chauhan 2004b | √ |  |  |  |  |  | √ |
| Chauhan 2004c | √ |  |  |  |  |  | √ |
| Chauhan 2004d | √ |  |  |  |  |  |  |
| Chauhan 2004e | √ |  |  |  |  |  |  |
| Hekmat 2004 |  |  |  |  | √ |  |  |
| Chauhan 2003 | √ |  |  |  |  |  |  |
| Pleym 2003 |  |  |  | √ | √ |  |  |
| Jares 2003 |  |  |  | √ | √ |  |  |
| Zabeeda 2002 |  |  |  | √ | √ |  |  |
| Leelahanon 2002 | √ |  |  |  |  |  |  |
| Özal 2002 | √ |  |  |  |  |  |  |
| Armelllin 2001 |  |  |  | √ | √ |  |  |
| Uozaki 2001 |  |  |  | √ |  |  |  |
| Casati V 2001a |  |  |  | √ | √ |  |  |
| Casati V 2001b |  |  |  |  | √ |  |  |
| Kamada 2001 |  |  |  | √ |  |  |  |
| Kojima 2001 |  |  |  |  | √ |  |  |
| Bonis 2000 |  |  |  | √ |  |  |  |
| Casati V 2000 |  |  |  |  | √ |  |  |
| Levin 2000 |  |  |  |  |  |  | √ |
| Nuttall 2000 |  |  |  |  | √ |  |  |
| Wong 2000 |  |  |  |  | √ |  |  |
| De Bonis 2000 |  |  | √ |  |  |  |  |
| Bernet 1999 |  |  |  |  | √ |  |  |
| Casati V 1999 |  |  |  |  | √ |  |  |
| Misfeld 1998 | √ |  |  | √ | √ |  |  |
| Mongan 1998 |  |  |  |  | √ |  |  |
| Hardy 1998 |  |  |  | √ |  |  |  |
| Katoh 1997a | √ |  |  |  |  |  |  |
| Katoh 1997b | √ |  |  |  |  |  |  |
| Brown R 1997 |  |  |  | √ | √ |  |  |
| Dryden 1997 |  |  |  |  | √ |  |  |
| Landymore 1997 |  |  |  | √ | √ |  |  |
| Pinosky 1997 | √ |  |  | √ | √ |  |  |
| Reid 1997 |  |  |  |  | √ |  |  |
| Katsaros 1996 | √ |  |  | √ | √ |  |  |
| Shore-L 1996 |  |  |  | √ | √ |  |  |
| Zonis 1996 |  |  |  |  | √ |  | √ |
| Menichetti 1996 | √ |  |  |  |  |  |  |
| Coffey 1995 | √ |  |  |  | √ |  |  |
| Corbeau 1995 | √ |  |  |  | √ |  |  |
| Horrow 1995a | √ |  |  |  | √ |  |  |
| Horrow 1995b | √ |  |  |  | √ |  |  |
| Horrow 1995c | √ |  |  |  | √ |  |  |
| Horrow 1995d | √ |  |  |  |  |  |  |
| Horrow 1995e | √ |  |  |  |  |  |  |
| Karski 1995a | √ |  |  |  |  |  |  |
| Karski 1995b | √ |  |  |  |  |  |  |
| Pugh 1995 |  |  |  | √ |  |  |  |
| Boughenous 1995 |  |  |  |  | √ |  |  |
| Rousou 1995 |  |  |  | √ |  |  |  |
| De Peppo1995 | √ |  |  | √ | √ |  |  |
| Speekenbrink 1995 |  |  |  | √ |  |  |  |
| Blauhut 1994 | √ |  |  |  |  |  |  |
| Isetta 1993 | √ |  |  |  |  |  |  |
| Horrow 1990 | √ |  |  |  |  |  |  |
| Horrow 1991a | √ |  |  |  |  |  |  |
| Horrow 1991b | √ |  |  |  |  |  |  |
| Total | 54 | 9 | 7 | 49 | 70 | 16 | 8 |
| Grand total (N) | 211 | | | | | | |
| Real total (r) | 130 | | | | | | |
| No. of meta (c) | 7 | | | | | | |
| CCA | 10.30% | | | | | | |

| Systematic review | Guo 2019 | Khaie 2019 |
| --- | --- | --- |
| Characteristics | cardiac surgery | cardiac surgery |
| Including study |  | |
| Mirmohammadsageghi 2018 | √ |  |
| Zhang Y 2018 | √ |  |
| Chaudhary 2018 | √ |  |
| Patel 2017 |  | √ |
| Myles 2017 | √ | √ |
| Van A 2016 |  | √ |
| Kimenai 2016 | √ |  |
| Shah 2015 | √ |  |
| Muthialu 2015 |  | √ |
| Ali Shah 2015 | √ |  |
| Hosseini 2014 | √ |  |
| Alizadeh 2014 | √ | √ |
| Faraoni 2014 |  | √ |
| Imtias 2014 |  | √ |
| Sigaut 2014 |  | √ |
| Du Y 2014 |  | √ |
| Esfandiari 2013 | √ | √ |
| Nouraei 2013 | √ |  |
| Chen T 2013 |  | √ |
| Shi 2013a | √ | √ |
| Shi 2013b |  | √ |
| Ahn 2012 | √ | √ |
| Bokesch 2012 |  | √ |
| Ghaffari 2012 |  | √ |
| Aoki 2012 | √ |  |
| Greiff 2012 | √ | √ |
| Mansouri 2012 |  | √ |
| Hassani 2012 | √ |  |
| Wang G 2012 | √ | √ |
| Baric D 2011 | √ |  |
| Hashemi 2011 | √ | √ |
| Shimizu 2011 |  | √ |
| Later 2009 | √ | √ |
| Taghaddomi 2009 | √ | √ |
| Fawzy 2009 | √ |  |
| Dietrich 2008 |  | √ |
| Fergusson 2008 |  | √ |
| Baric 2007 | √ |  |
| Maddali 2007 | √ | √ |
| Mehr-Aein 2007 | √ | √ |
| Abul-Azm 2006 | √ |  |
| Kuitunen 2006 |  | √ |
| Murphy 2006 | √ | √ |
| Wei M 2006 |  | √ |
| Bulutcu 2005 |  | √ |
| Diprose 2005 | √ | √ |
| Karski 2005 |  | √ |
| Kuitunen 2005 |  | √ |
| Vanek 2005 |  | √ |
| Andreasen 2004 | √ | √ |
| Casati 2004a | √ | √ |
| Chauhan 2004a |  | √ |
| Hekmat 2004 |  | √ |
| Pleym 2003 | √ | √ |
| Jares 2003 | √ | √ |
| Zabeeda 2002 | √ | √ |
| Armelllin 2001 | √ | √ |
| Uozaki 2001 | √ |  |
| Casati V 2001a | √ | √ |
| Casati V 2001b |  | √ |
| Kamada 2001 | √ |  |
| Kojima 2001 |  | √ |
| Bonis 2000 | √ |  |
| Casati V 2000 |  | √ |
| Nuttall 2000 |  | √ |
| Wong 2000 |  | √ |
| Bernet 1999 |  | √ |
| Casati V 1999 |  | √ |
| Misfeld 1998 | √ | √ |
| Mongan 1998 |  | √ |
| Hardy 1998 | √ |  |
| Brown R 1997 | √ | √ |
| Dryden 1997 |  | √ |
| Landymore 1997 | √ | √ |
| Pinosky 1997 | √ | √ |
| Reid 1997 |  | √ |
| Katsaros 1996 | √ | √ |
| Shore-L 1996 | √ | √ |
| Zonis 1996 |  | √ |
| Coffey 1995 |  | √ |
| Corbeau 1995 |  | √ |
| Horrow 1995a |  | √ |
| Horrow 1995b |  | √ |
| Horrow 1995c |  | √ |
| Pugh 1995 | √ |  |
| Boughenous 1995 |  | √ |
| Rousou 1995 | √ |  |
| De Peppo1995 | √ | √ |
| Speekenbrink 1995 | √ |  |
| Total | 49 | 68 |
| Grand total (N) | 117 | |
| Real total (r) | 88 | |
| No. of meta (c) | 2 | |
| CCA | 32.90% | |

CCA =Corrected covered area. Calculation = CCA (%)=N-r/rc-r:

Where N=number of included publications (sum of checked boxes), r = number of rows (primary studies), c =number of columns (number of systematic reviews)

D. Cerebrology

| Systematic review | Chen 2019 | Weng 2018 | Zehtabchi 2016 | July 2020 | ElMenyar 2018 | Gao 2020 | Alhelaly 2019 | Yokobori 2020 |
| --- | --- | --- | --- | --- | --- | --- | --- | --- |
| Characteristics | traumatic brain injury | traumatic brain injury | traumatic brain injury | traumatic brain injury | traumatic brain injury | traumatic brain injury | traumatic brain injury | traumatic brain injury |
| Including study |  | | | | | | | |
| CRASH-3 2019 |  |  |  | √ |  |  |  | √ |
| Ebrahimi 2019 |  |  |  |  |  |  |  | √ |
| Fakharian 2018 | √ | √ |  | √ |  | √ | √ | √ |
| Chakroun-Walha 2018 | √ | √ |  | √ |  |  |  | √ |
| Jokar 2017 | √ | √ |  | √ |  | √ | √ | √ |
| May-17 |  |  |  |  | √ |  |  |  |
| Zehtabchi 2014 |  |  |  |  | √ |  |  |  |
| Yutthakasemsunt 2013 | √ | √ | √ | √ |  | √ | √ | √ |
| CRASH-2 2013 |  |  |  | √ |  | √ |  |  |
| Roberts 2013 | √ |  |  |  |  |  |  |  |
| Perel 2012 |  | √ |  |  |  |  |  |  |
| Crash-2C 2011 | √ |  | √ |  |  |  | √ | √ |
| NCT 1990 |  |  |  | √ |  |  |  |  |
| Total | 6 | 5 | 2 | 7 | 2 | 4 | 4 | 7 |
| Grand total (N) | 37 | | | | | | | |
| Real total (r) | 13 | | | | | | | |
| No. of meta (c) | 8 | | | | | | | |
| CCA | 26.30% | | | | | | | |

| Systematic review | Hu 2019 | Huang 2018 | Gao 2020 |
| --- | --- | --- | --- |
| Characteristics | cerebral hemorrhage | cerebral hemorrhage | cerebral hemorrhage |
| Including study |  | | |
| Sprigg 2018 | √ |  |  |
| TICH2 2018 |  |  | √ |
| Fakharian 2018 |  | √ |  |
| Joker 2017 | √ | √ |  |
| Arumugam 2015 | √ |  | √ |
| Sprigg 2014 | √ | √ |  |
| TICH1 2014 |  |  | √ |
| Yutthakasemsunt 2013 | √ | √ |  |
| Perel 2012 | √ |  |  |
| Crash-2 2011 |  | √ |  |
| Hillman 2002 | √ |  |  |
| Roos 2000 | √ | √ |  |
| Tsementzis 1990 | √ |  |  |
| Vermeulen 1984 | √ | √ |  |
| Foodstad 1981 | √ |  |  |
| Kaste 1979 | √ |  |  |
| Maurice-W 1978 | √ |  |  |
| Gibbs 1971 | √ |  |  |
| Total | 14 | 7 | 3 |
| Grand total (N) | 24 | | |
| Real total (r) | 17 | | |
| No. of meta (c) | 3 | | |
| CCA | 20.5% | | |

CCA =Corrected covered area. Calculation = CCA (%)=N-r/rc-r:

Where N=number of included publications (sum of checked boxes), r = number of rows (primary studies), c =number of columns (number of systematic reviews)

E. Total knee arthroplasty

| Systematic review | Guo 2018 | Zhang 2017 |
| --- | --- | --- |
| Characteristics | total knee arthroplasty | total knee arthroplasty |
| Administration | oral | oral |
| Including study |  | |
| Lee 2017 | √ | √ |
| Yuan 2017 | √ | √ |
| Perreault 2017 |  | √ |
| Alipour 2013 | √ | √ |
| Bradshaw 2012 | √ | √ |
| Zohar 2004 | √ | √ |
| Total | 5 | 6 |
| Grand total (N) | 11 | |
| Real total (r) | 6 | |
| No. of meta (c) | 2 | |
| CCA | 83.30% | |

| Systematic review | Tian 2017 | Wu 2017 | Chen TP 2017 | Yu 2015 | Chen X 2015 | Tan 2013 | Fu 2013 | Yang 2012 | Cid 2005 | Chen 2015 |
| --- | --- | --- | --- | --- | --- | --- | --- | --- | --- | --- |
| Characteristics | total knee arthroplasty | total knee arthroplasty | total knee arthroplasty | total knee arthroplasty | total knee arthroplasty | total knee arthroplasty | total knee arthroplasty | total knee arthroplasty | total knee arthroplasty | total knee arthroplasty |
| Administration | intravenous | intravenous | intravenous | intravenous | intravenous | intravenous | intravenous | intravenous | intravenous | intravenous |
| Including study |  | | | | | | | | | |
| Ortega-Andreu 2016 | √ |  |  |  |  |  |  |  |  |  |
| Chen 2016 |  | √ |  |  |  |  |  |  |  |  |
| Shinde 2015 |  | √ |  |  |  |  |  |  |  |  |
| Digas 2015 |  |  | √ |  |  |  |  |  |  |  |
| Hou 2015 |  |  | √ |  |  |  |  |  |  |  |
| Han 2015 |  |  | √ |  |  |  |  |  |  |  |
| Shen 2015 |  |  |  | √ |  |  |  |  |  |  |
| Jiang 2015 |  |  | √ |  |  |  |  |  |  |  |
| Aguilera 2015 |  |  | √ |  |  |  |  |  |  |  |
| Samujh 2014 | √ |  |  |  |  |  |  |  |  |  |
| Kin 2014 |  | √ |  |  |  |  |  |  |  |  |
| Sarzaeem 2014 |  |  | √ |  |  |  |  |  |  |  |
| Soni 2014 |  |  | √ |  |  |  |  |  |  |  |
| Tang 2014 |  |  | √ |  |  |  |  |  |  |  |
| Han 2014 |  |  | √ |  |  |  |  |  |  |  |
| Zhai 2014 |  |  |  |  | √ |  |  |  |  | √ |
| Patel 2014 |  |  | √ |  |  |  |  |  |  |  |
| Hafeez 2014 |  | √ |  |  |  |  |  |  |  |  |
| Zhang and Xu 2013 |  |  |  |  | √ |  |  |  |  | √ |
| Zhang 2013 |  |  |  |  | √ |  |  |  |  | √ |
| Smit 2013 | √ |  |  |  |  |  |  |  |  |  |
| Seo 2013 |  |  | √ | √ |  |  |  |  |  |  |
| Maniar 2012 |  |  | √ | √ |  | √ |  |  |  |  |
| Lee 2012 |  |  |  | √ |  |  | √ |  |  |  |
| McConnell 2012 |  |  |  | √ |  |  | √ |  |  |  |
| Fu 2012 |  |  |  |  | √ |  |  |  |  | √ |
| Aguilera 2012 | √ |  |  |  |  |  |  |  |  |  |
| Chareancholvanich  2012 |  |  |  | √ |  |  | √ |  |  |  |
| Charoencholvanich 2011 |  |  |  | √ |  | √ | √ |  |  |  |
| Lin 2011 |  |  |  | √ |  |  |  |  |  |  |
| MacGillivray 2011 |  | √ |  | √ |  |  |  | √ |  |  |
| Gautam 2011 |  |  |  | √ |  |  | √ |  |  |  |
| Dhillon 2011 |  |  |  | √ |  |  |  |  |  |  |
| Wong 2010 |  |  |  |  |  |  |  | √ |  |  |
| Kakar 2009 |  | √ |  | √ |  | √ | √ | √ |  |  |
| Alvarez 2008 |  |  |  | √ |  | √ | √ | √ |  |  |
| Zhang 2007 |  |  |  | √ |  | √ | √ | √ |  |  |
| Molloy 2007 |  |  |  | √ |  |  | √ |  |  |  |
| Orpen 2006 |  |  |  | √ |  | √ | √ | √ |  |  |
| Camarasa 2006 |  |  |  | √ | √ | √ | √ | √ |  | √ |
| Johansson 2005 |  |  |  |  | √ |  |  |  |  | √ |
| Zohar 2004 |  |  |  | √ |  | √ |  |  |  |  |
| Good 2003 |  |  |  | √ | √ | √ | √ | √ | √ | √ |
| Veien 2002 |  |  |  | √ |  | √ | √ | √ | √ |  |
| Ellis 2001 |  |  |  | √ |  | √ | √ | √ | √ |  |
| Engel 2001 |  |  |  | √ |  | √ | √ |  | √ |  |
| Tanaka 2001 |  |  |  | √ |  | √ | √ | √ | √ |  |
| Ido 2000 |  |  |  | √ |  | √ | √ |  |  |  |
| Jansen 1999 |  |  |  | √ |  | √ | √ | √ | √ |  |
| Hiippala 1997 |  |  |  | √ |  | √ | √ | √ | √ |  |
| Benoni 1996 |  |  |  | √ |  | √ | √ | √ | √ |  |
| Hiippala 1995 |  |  |  | √ |  | √ | √ | √ | √ |  |
| Total | 5 | 6 | 12 | 28 | 7 | 19 | 22 | 15 | 9 | 7 |
| Grand total (N) | 130 | | | | | | | | | |
| Real total (r) | 51 | | | | | | | | | |
| No. of meta (c) | 10 | | | | | | | | | |
| CCA | 17.21% | | | | | | | | | |

| Systematic review | Wu 2017 | Tan 2013 |
| --- | --- | --- |
| Characteristics | total knee arthroplasty | total knee arthroplasty |
| Administration | intravenous | intravenous |
| Including study |  | |
| Ortega-Andreu 2016 |  |  |
| Chen 2016 | √ |  |
| Shinde 2015 | √ |  |
| Kin 2014 | √ |  |
| Hafeez 2014 | √ |  |
| Maniar 2012 |  |  |
| Charoencholvanich 2011 |  | √ |
| MacGillivray 2011 | √ | √ |
| Kakar 2009 | √ |  |
| Alvarez 2008 |  | √ |
| Zhang 2007 |  | √ |
| Orpen 2006 |  | √ |
| Camarasa 2006 |  | √ |
| Zohar 2004 |  | √ |
| Good 2003 |  | √ |
| Veien 2002 |  | √ |
| Ellis 2001 |  | √ |
| Engel 2001 |  | √ |
| Tanaka 2001 |  | √ |
| Ido 2000 |  | √ |
| Jansen 1999 |  | √ |
| Hiippala 1997 |  | √ |
| Benoni 1996 |  | √ |
| Hiippala 1995 |  | √ |
| Total | 6 | √ |
| Grand total (N) | 25 | |
| Real total (r) | 23 | |
| No. of meta (c) | 2 | |
| CCA | 8.69% | |

| Systematic review | Moskal 2017 | Zhang 2014 | Chen Z 2014 | Panteli 2013 |
| --- | --- | --- | --- | --- |
| Characteristics | total knee arthroplasty | total knee arthroplasty | total knee arthroplasty | total knee arthroplasty |
| Administration | topical | topical | topical | topical |
| Including Study |  | | | |
| Yang 2015 | √ |  |  |  |
| Chen 2014 | √ |  |  |  |
| Craik 2014 | √ |  |  |  |
| Gilbody 2014 | √ |  |  |  |
| Jang 2014 | √ |  |  |  |
| Alshryda 2013 | √ | √ |  |  |
| Seo 2013 | √ | √ | √ |  |
| Chimento 2013 | √ |  |  |  |
| Georgiadis 2013 | √ | √ |  |  |
| Konig 2013 | √ |  |  |  |
| Maniar 2012 | √ |  |  | √ |
| Mutsuzaki 2012 | √ |  |  |  |
| Onodera 2012 |  |  |  | √ |
| Roy 2012 | √ | √ | √ | √ |
| Ishida 2011 |  | √ | √ | √ |
| Rajesh 2011 |  |  | √ |  |
| Sa-Ngasoongsong 2011 | √ | √ | √ | √ |
| Abrishami 2010 |  |  |  | √ |
| Wong 2010 | √ | √ | √ | √ |
| Total | 15 | 7 | 6 | 7 |
| Grand total (N) | 35 | | | |
| Real total (r) | 19 | | | |
| No. of meta (c) | 4 | | | |
| CCA | 28.07% | | | |

| Systematic review | Huang C 2020 | Li 2020 | Xiong 2018 | Dai 2018 | Wang 2017 | Meena 2017 | Mi B 2017 | Gao 2016 | Lin 2016 | Fu 2016 | Wen 2016 | Shemshaki 2015 | Wu 2015 | Wang 2014 |
| --- | --- | --- | --- | --- | --- | --- | --- | --- | --- | --- | --- | --- | --- | --- |
| Characteristics | TKA | TKA | TKA | TKA | TKA | TKA | TKA | TKA | TKA | TKA | TKA | TKA | TKA | TKA |
| Administration | IV and topical | IV and topical | IV and topical | IV and topical | IV and topical | IV and topical | IV and topical | IV and topical | IV and topical | IV and topical | IV and topical | IV and topical | IV and topical | IV and topical |
| Including Study |  |  |  |  |  |  |  |  |  |  |  |  |  |  |
| Zhang 2019 | √ |  |  |  |  |  |  |  |  |  |  |  |  |  |
| Kethy 2019 |  | √ |  |  |  |  |  |  |  |  |  |  |  |  |
| Laoruengthana 2019 |  | √ |  |  |  |  |  |  |  |  |  |  |  |  |
| Adravanti 2018 | √ |  |  |  |  |  |  |  |  |  |  |  |  |  |
| Abdel 2018 |  | √ |  |  |  |  |  |  |  |  |  |  |  |  |
| Ahmed 2018 |  | √ |  |  |  |  |  |  |  |  |  |  |  |  |
| Arora 2018 |  | √ |  |  |  |  |  |  |  |  |  |  |  |  |
| George 2018 |  | √ |  |  |  |  |  |  |  |  |  |  |  |  |
| Subramanyam 2018 |  | √ |  |  |  |  |  |  |  |  |  |  |  |  |
| Zecker 2018 |  | √ |  |  |  |  |  |  |  |  |  |  |  |  |
| Yuan 2018 | √ | √ |  |  |  |  |  |  |  |  |  |  |  |  |
| Lee 2017 | √ |  | √ |  | √ |  |  |  |  |  |  |  |  |  |
| Alvaro 2017 |  | √ |  |  |  |  |  |  |  |  |  |  |  |  |
| Lacko 2017 |  | √ |  |  |  |  |  |  |  |  |  |  |  |  |
| Prakash 2017 |  | √ |  |  |  |  |  |  |  |  |  |  |  |  |
| Stowers 2017 |  | √ |  |  |  |  |  |  |  |  |  |  |  |  |
| Balasubramanian 2016 |  | √ |  |  |  |  |  |  |  |  |  |  |  |  |
| Song 2016 | √ | √ | √ |  | √ |  |  |  |  |  |  |  |  |  |
| Tzatzairis 2016 |  | √ |  | √ |  |  | √ |  |  | √ |  |  |  |  |
| May 20 16 |  | √ |  | √ |  |  | √ |  |  | √ |  |  |  |  |
| Drosos 2016 |  | √ |  | √ |  |  | √ |  |  |  |  |  |  |  |
| Aggarwal 2016 |  | √ |  |  |  |  | √ |  |  |  |  |  |  |  |
| Goyal 2016 |  |  |  |  |  |  |  |  |  |  |  |  |  |  |
| Hua 2016 |  |  |  |  |  |  |  |  |  |  |  |  |  |  |
| Xu 2016 |  |  |  |  |  |  |  |  |  |  |  |  |  |  |
| Zhao 2016 |  |  |  |  |  |  |  |  | √ |  |  |  |  |  |
| Pinsornsak 2016 |  | √ |  |  |  |  | √ |  |  |  |  |  |  |  |
| Zekcer 2016 |  | √ |  |  |  |  | √ |  |  |  |  |  |  |  |
| Ugurlu 2016 |  | √ |  | √ |  |  |  |  |  | √ |  |  |  |  |
| Nielsen 2016 | √ |  | √ |  | √ |  |  |  | √ |  |  |  |  |  |
| Jain 2016 | √ |  | √ |  | √ |  |  |  | √ |  |  |  |  |  |
| Oztas 2015 |  | √ |  | √ |  |  |  |  |  | √ |  |  |  |  |
| Cacusoglu 2015 |  |  |  | √ |  |  |  |  |  |  |  |  |  |  |
| Karaaslan 2015 |  |  |  |  | √ |  |  |  | √ |  | √ |  |  |  |
| Wang 2015 |  |  |  |  |  |  |  |  | √ |  | √ |  |  |  |
| Zhao 2015(a) |  |  |  |  |  |  |  |  | √ |  |  |  |  |  |
| Tu 2015 |  |  |  |  |  |  |  |  | √ |  |  |  |  |  |
| Cui and Wu 2015 |  |  |  |  |  |  |  |  | √ |  |  |  |  |  |
| Zhao 2015(b) |  |  |  |  |  |  |  |  | √ |  |  |  |  |  |
| Keyhani 2015 |  | √ |  |  |  |  | √ |  |  | √ |  |  |  |  |
| Aguilera 2015 |  | √ |  | √ |  | √ | √ |  |  |  |  |  |  |  |
| Bagsby 2015 |  |  |  |  |  |  |  |  |  |  | √ |  |  |  |
| Gu 2015 |  |  |  |  |  |  |  |  |  |  | √ |  |  |  |
| Digas 2015 |  | √ |  | √ |  | √ | √ |  |  | √ |  |  |  |  |
| Liu 2015 |  | √ | √ |  |  |  |  |  | √ |  |  |  |  |  |
| Lin 2015 | √ |  |  |  | √ |  |  |  | √ |  |  |  |  |  |
| Huang 2014 | √ |  | √ |  | √ |  |  |  | √ |  |  |  |  |  |
| Zhao 2014 |  |  |  |  |  |  |  |  |  |  | √ |  |  |  |
| Soni 2014 |  | √ |  | √ |  | √ | √ |  |  | √ |  |  |  | √ |
| Gomez-Barrena 2014 |  | √ |  | √ |  | √ | √ |  |  | √ |  |  |  |  |
| Patel 2014 |  | √ |  | √ |  | √ | √ |  |  | √ |  |  |  | √ |
| Sarzaeem 2014 |  | √ |  | √ |  | √ | √ |  |  | √ |  |  |  | √ |
| Xu 2014 |  |  |  |  |  |  |  | √ |  |  |  |  |  |  |
| Kim 2014 |  |  |  |  |  |  |  |  |  |  | √ |  | √ |  |
| Karam 2014 |  |  |  |  |  |  |  |  |  |  | √ |  |  |  |
| Kelley 2014 |  |  |  |  |  |  |  |  |  |  | √ |  |  |  |
| Mutsuzaki 2014 |  |  |  |  |  |  |  |  |  |  | √ |  |  |  |
| He 2014 |  |  |  |  |  |  |  |  |  |  | √ |  |  |  |
| Martin 2014 |  |  |  |  |  |  |  |  |  |  |  | √ | √ |  |
| Oremus 2014 |  |  |  |  |  |  |  |  |  |  |  | √ |  |  |
| Sabatini 2014 |  |  |  |  |  |  |  | √ |  |  |  |  |  |  |
| Pachauri 2013 |  |  |  |  |  |  |  |  |  |  |  |  | √ |  |
| Georgiadis 2013 |  |  |  |  |  |  |  |  |  |  |  | √ | √ |  |
| Lee 2013 |  |  |  |  |  |  |  |  |  |  |  | √ | √ |  |
| Alshryda 2013 |  |  |  |  |  |  |  |  |  |  |  |  | √ |  |
| Aguilera 2013 |  |  |  |  |  |  |  | √ |  | √ |  |  | √ |  |
| Hegde 2013 |  | √ |  |  |  |  |  |  |  |  | √ |  |  | √ |
| Seo 2013 |  | √ |  | √ |  | √ | √ |  |  | √ |  |  | √ | √ |
| Sa-Ngasoongsong 2013 |  |  |  |  |  |  |  |  |  |  |  | √ |  |  |
| Maniar 2012 |  | √ |  | √ |  | √ | √ |  |  | √ |  | √ | √ | √ |
| McConnelll 2012 |  |  |  |  |  |  |  | √ |  |  |  | √ | √ |  |
| Charencholvanich 2012 |  |  |  |  |  |  |  |  |  |  |  |  | √ |  |
| Raviraj 2012 |  |  |  |  |  |  |  |  |  |  | √ |  |  |  |
| Roy 2012 |  |  |  |  |  |  |  |  |  |  |  | √ | √ |  |
| Onodera 2012 |  |  |  |  |  |  |  |  |  |  |  |  | √ |  |
| Ishida 2011 |  |  |  |  |  |  |  |  |  |  |  | √ | √ |  |
| Gautum 2011 |  |  |  |  |  |  |  |  |  |  |  |  | √ |  |
| Sa-Ngasoongsong 2011 |  |  |  |  |  |  |  |  |  |  |  | √ |  |  |
| Charencholvanich 2011 |  |  |  |  |  |  |  |  |  |  |  |  | √ |  |
| MacGillivray 2011 |  |  |  |  |  |  |  |  |  |  | √ | √ |  |  |
| Dhillon 2011 |  |  |  |  |  |  |  |  |  |  | √ |  |  |  |
| Wong 2010 |  |  |  |  |  |  |  |  |  |  |  | √ | √ |  |
| Abrishami 2009 |  |  |  |  |  |  |  |  |  |  |  |  | √ |  |
| Kakar 2009 |  |  |  |  |  |  |  |  |  |  | √ | √ | √ |  |
| Alvarez 2008 |  |  |  |  |  |  |  |  |  |  |  |  | √ |  |
| Zhang 2007 |  |  |  |  |  |  |  |  |  |  |  | √ | √ |  |
| Molloy 2007 |  |  |  |  |  |  |  | √ |  |  |  | √ | √ |  |
| Camarasa 2006 |  |  |  |  |  |  |  |  |  |  |  |  | √ |  |
| Orpen 2006 |  |  |  |  |  |  |  |  |  |  |  | √ | √ |  |
| Zohar 2004 |  |  |  |  |  |  |  |  |  |  |  | √ | √ |  |
| Good 2003 |  |  |  |  |  |  |  |  |  |  |  | √ | √ |  |
| Veien 2002 |  |  |  |  |  |  |  |  |  |  |  | √ | √ |  |
| Engel 2001 |  |  |  |  |  |  |  |  |  |  |  | √ |  |  |
| Tanaka 2001 |  |  |  |  |  |  |  |  |  |  |  | √ | √ |  |
| Ellis 2001 |  |  |  |  |  |  |  |  |  |  |  | √ | √ |  |
| Engel 2001 |  |  |  |  |  |  |  |  |  |  |  |  | √ |  |
| Jansen 1999 |  |  |  |  |  |  |  |  |  |  |  |  | √ |  |
| Hippala 1997 |  |  |  |  |  |  |  |  |  |  |  | √ | √ |  |
| Benoni 1996 |  |  |  |  |  |  |  |  |  |  |  |  | √ |  |
| Hippala 1995 |  |  |  |  |  |  |  |  |  |  |  | √ | √ |  |
| Jansen 1994 |  |  |  |  |  |  |  |  |  |  |  | √ |  |  |
| Total | 9 | 34 | 6 | 15 | 7 | 8 | 16 | 5 | 13 | 14 | 15 | 31 | 34 | 6 |
| Grand total (N) | 213 | | | | | | | | | | | | | |
| Real total (r) | 101 | | | | | | | | | | | | | |
| No. of meta (c) | 14 | | | | | | | | | | | | | |
| CCA | 8.53% | | | | | | | | | | | | | |

Red: Not RCT; TKA= total knee arthroplasty; IV=intravenous

CCA =Corrected covered area. Calculation = CCA (%)=N-r/rc-r:

Where N=number of included publications (sum of checked boxes), r = number of rows (primary studies), c =number of columns (number of systematic reviews)

F. Total hip arthroplasty

| Systematic review | Sukeik 2019 | Xin 2019 | He 2015 | Zhou 2013 | Sukeik 2010 |
| --- | --- | --- | --- | --- | --- |
| Characteristics | total hip arthroplasty | total hip arthroplasty | total hip arthroplasty | total hip arthroplasty | total hip arthroplasty |
| Administration | intravenous | intravenous | intravenous | intravenous | intravenous |
| Including Study |  | | | | |
| Reichel 2018 |  | √ |  |  |  |
| Peck 2018 |  | √ |  |  |  |
| Mariani 2017 |  | √ |  |  |  |
| Fernandez 2017 | √ |  |  |  |  |
| Melo 2017 | √ |  |  |  |  |
| Fraval 2017 | √ |  |  |  |  |
| Zhang 2016 | √ |  |  |  |  |
| Wang 2016 | √ |  |  |  |  |
| Barrachina 2016 | √ |  |  |  |  |
| Park 2016 |  | √ |  |  |  |
| Duncan 2015 |  | √ |  |  |  |
| Jaszczyk 2015 | √ |  |  |  |  |
| Hsu 2015 | √ |  |  |  |  |
| Oremus 2014 | √ |  |  |  |  |
| Kakar 2014 |  |  | √ |  |  |
| Karam 2014 |  |  | √ |  |  |
| Kim 2013 |  |  | √ |  |  |
| MacGillivray 2013 |  |  | √ |  |  |
| Lee 2013 | √ |  |  |  |  |
| Norio 2012 |  |  |  | √ |  |
| Fu 2012 |  |  |  | √ |  |
| Imai 2012 | √ |  |  |  |  |
| Clave 2012 |  |  |  | √ |  |
| Kazi 2012 |  | √ |  |  |  |
| Jamie 2011 |  |  |  | √ |  |
| McConnell 2011 | √ |  |  |  |  |
| Malhotra 2011 | √ |  |  | √ |  |
| Hegde 2011 |  |  | √ |  |  |
| Singh 2010 |  |  |  | √ |  |
| Kazemi 2010 | √ |  |  | √ |  |
| Gill 2009 |  | √ |  |  |  |
| Rahesparan 2009 |  |  |  | √ |  |
| Dhilon 2009 |  |  | √ |  |  |
| Claeys 2007 | √ |  |  | √ | √ |
| Phillips 2006 |  | √ |  |  |  |
| Johansson 2005 | √ |  |  | √ | √ |
| Niskanen 2005 | √ |  |  | √ | √ |
| Garneti 2004 | √ |  |  | √ | √ |
| Yamasaki 2004 | √ |  |  | √ | √ |
| Husted 2003 | √ |  |  | √ | √ |
| Lemay 2003 | √ |  |  | √ | √ |
| Benoni 2001 | √ |  |  | √ | √ |
| Benoni 2000 | √ |  |  | √ | √ |
| Ekback 2000 | √ |  |  | √ | √ |
| Ido 2000 | √ |  |  | √ | √ |
| Total | 25 | 7 | 6 | 19 | 11 |
| Grand total (N) | 68 | | | | |
| Real total (r) | 45 | | | | |
| No. of meta (c) | 5 | | | | |
| CCA | 12.78% | | | | |

| Systematic review | Chen S 2016 | Xu 2015 | Wang 2015 |
| --- | --- | --- | --- |
| Characteristics | total hip arthroplasty | total hip arthroplasty | total hip arthroplasty |
| Administration | topical | topical | topical |
| Including Study |  | | |
| Yue C 2014 | √ | √ | √ |
| Yin 2014 | √ |  |  |
| Machin 2014 | √ |  |  |
| BagsBy 2014 | √ |  | √ |
| Chang 2014 | √ |  | √ |
| Ding 2014 | √ |  |  |
| Fan 2014 | √ |  |  |
| Gilbody 2014 | √ |  |  |
| Martin JG 2014 | √ | √ | √ |
| Wind 2014 | √ |  | √ |
| Wei 2014 | √ | √ | √ |
| Van Elst 2013 | √ |  |  |
| Konig G 2013 | √ | √ | √ |
| Alshryda 2013 | √ | √ | √ |
| Total | 14 | 4 | 8 |
| Grand total (N) | 26 | | |
| Real total (r) | 14 | | |
| No. of meta (c) | 3 | | |
| CCA | 42.86% | | |

| Systematic review | Wang 2019 | Wang 2018 | Zhang 2018 | Wu Y 2018 |
| --- | --- | --- | --- | --- |
| Characteristics | total hip arthroplasty | total hip arthroplasty | total hip arthroplasty | total hip arthroplasty |
| Administration | oral and intravenous | oral and intravenous | oral and intravenous | oral and intravenous |
| Including Study |  | | | |
| Gortemoller 2018 |  | √ |  |  |
| Wu 2018 |  | √ |  |  |
| Liu 2018 |  |  | √ | √ |
| Zhao 2018 | √ | √ |  |  |
| Cao 2018 | √ |  |  |  |
| Luo 2018 | √ | √ |  |  |
| Kayupov 2017 | √ | √ |  |  |
| Zhang JK 2017 |  |  | √ | √ |
| Zhang JZ 2017 |  |  | √ | √ |
| Wang 2017 |  |  | √ |  |
| Jans 2016 |  |  | √ | √ |
| Gao 2015 |  |  | √ | √ |
| Total | 4 | 5 | 6 | 5 |
| Grand total (N) | 20 | | | |
| Real total (r) | 12 | | | |
| No. of meta (c) | 4 | | | |
| CCA | 22.22% | | | |

| Systematic review | Sun YB 2017 | Zhang H 2017 | Liu 2017 | Zhang P 2017 | Li J 2016 | Sun 2016 |
| --- | --- | --- | --- | --- | --- | --- |
| Characteristics | total hip arthroplasty | total hip arthroplasty | total hip arthroplasty | total hip arthroplasty | total hip arthroplasty | total hip arthroplasty |
| Administration | intravenous and topical | intravenous and topical | intravenous and topical | intravenous and topical | intravenous and topical | intravenous and topical |
| Including Study |  | | | | | |
| Wu 2016 | √ | √ |  |  |  |  |
| Zeng 2016 | √ |  |  | √ |  |  |
| Zhu 2016 |  | √ |  | √ |  |  |
| Yi 2016 |  | √ | √ |  |  |  |
| Lu 2016 |  | √ | √ | √ |  |  |
| Ueno 2016 |  |  |  |  | √ | √ |
| Zhao 2016 |  | √ | √ | √ |  |  |
| Sun 2016 | √ |  | √ |  |  |  |
| Yue 2015 |  |  |  | √ | √ |  |
| Zhang 2015 | √ | √ | √ | √ | √ |  |
| North 2015 |  |  |  |  | √ | √ |
| Xie 2015 | √ | √ | √ | √ | √ | √ |
| Wind 2014 |  |  |  |  | √ | √ |
| Wei 2014 |  |  |  |  | √ | √ |
| Machin 2014 |  | √ |  |  |  |  |
| Total | 5 | 8 | 6 | 7 | 7 | 5 |
| Grand total (N) | 38 | | | | |  |
| Real total (r) | 15 | | | | |  |
| No. of meta (c) | 6 | | | | |  |
| CCA | 30.67% | | | | |  |

| Systematic review | Ye 2020 | Sun 2020 | Han 2018 | Chen 2019 |
| --- | --- | --- | --- | --- |
| Characteristics | total hip arthroplasty and total knee arthroplasty | total hip arthroplasty and total knee arthroplasty | total hip arthroplasty and total knee arthroplasty | total hip arthroplasty and total knee arthroplasty |
| Administration | oral and intravenous | oral and intravenous | oral and intravenous | oral and intravenous |
| Including Study |  | | | |
| Luo 2018 | √ | √ | √ | √ |
| Cao-K 2018 | √ | √ | √ |  |
| Zhao 2018 | √ | √ |  |  |
| Cao-H 2018 | √ | √ |  |  |
| Wang 2018 | √ | √ | √ |  |
| Wu 2018 | √ | √ |  |  |
| Kayupov 2017 | √ | √ | √ | √ |
| Gortemoller 2017 |  |  | √ | √ |
| Yuan 2017 | √ | √ | √ | √ |
| Fillingham 2016 | √ | √ | √ | √ |
| Irwin 2013 |  |  | √ | √ |
| Zohar-L 2004 | √ | √ | √ | √ |
| Zohar-S 2005 | √ |  |  |  |
| Total | 11 | 10 | 9 | 7 |
| Grand total (N) | 37 | | | |
| Real total (r) | 13 | | | |
| No. of meta (c) | 4 | | | |
| CCA | 61.54% | | | |

| Systematic review | Sun 2019 | Arianna 2018 | Zhang 2017 | Mi 2017 | Xie 2017 | Li F 2017 | Chen 2016 | Shang 2016 |
| --- | --- | --- | --- | --- | --- | --- | --- | --- |
| Characteristics | total hip arthroplasty and total knee arthroplasty | total hip arthroplasty and total knee arthroplasty | total hip arthroplasty and total knee arthroplasty | total hip arthroplasty and total knee arthroplasty | total hip arthroplasty and total knee arthroplasty | total hip arthroplasty and total knee arthroplasty | total hip arthroplasty and total knee arthroplasty | total hip arthroplasty and total knee arthroplasty |
| Administration | oral and intravenous | oral and intravenous | oral and intravenous | oral and intravenous | oral and intravenous | oral and intravenous | oral and intravenous | oral and intravenous |
| Including Study |  | | | | | | | |
| Song 2017 |  | √ |  | √ |  | √ |  |  |
| Goyal 2017 |  | √ |  |  | √ |  |  |  |
| Lee 2017 |  |  | √ |  |  |  |  |  |
| Yuan 2017 |  |  | √ |  |  |  |  |  |
| Perreault 2017 |  |  | √ |  |  |  |  |  |
| Ugurlu 2017 | √ | √ |  |  | √ |  |  |  |
| Zhang 2016 | √ | √ |  |  | √ |  | √ |  |
| Drosos 2016 | √ | √ |  |  | √ |  |  |  |
| Chen 2016 | √ | √ |  |  | √ |  | √ |  |
| Aggarwal 2016 | √ | √ |  |  | √ |  | √ |  |
| Xie 2016 | √ | √ |  |  | √ | √ | √ | √ |
| Zeng 2016 |  |  |  |  |  |  |  | √ |
| Keyhani 2016 | √ | √ |  |  | √ |  |  |  |
| May 20 16 | √ | √ |  |  | √ |  |  |  |
| Pinsornsak 2016 |  | √ |  |  |  |  |  |  |
| Pitta 2016 |  | √ |  |  |  |  |  |  |
| North 2016 | √ | √ |  |  | √ |  | √ |  |
| Tzatzairis 2016 | √ | √ |  |  | √ |  |  |  |
| Ueno 2016 |  | √ |  |  |  |  |  |  |
| Nielsen 2016 | √ | √ |  | √ |  | √ |  | √ |
| Jain 2016 | √ | √ |  | √ |  | √ |  | √ |
| Yi 2016 | √ | √ |  |  |  |  |  |  |
| Wu 2016 | √ | √ |  |  |  | √ |  |  |
| Digas 2015 | √ | √ |  |  | √ |  | √ |  |
| Hamlin 2015 |  | √ |  |  |  |  |  |  |
| Oztas 2015 | √ |  |  |  | √ |  |  |  |
| Aguilera 2015 | √ | √ |  |  |  |  | √ |  |
| Hou 2015 |  |  |  |  |  |  | √ |  |
| Jiang 2015 |  |  |  |  |  |  | √ |  |
| Liu 2015 |  |  |  |  |  |  | √ |  |
| Chai 2015 |  |  |  |  |  |  | √ |  |
| Cavusoglu 2015 |  | √ |  |  | √ |  |  |  |
| Lin 2015 | √ |  |  | √ |  |  |  |  |
| Patel 2014 | √ | √ |  |  | √ |  | √ |  |
| Sarzaeem 2014 | √ | √ |  |  | √ |  | √ |  |
| Gomez-Barrena 2014 | √ | √ |  |  | √ |  | √ |  |
| Soni 2014 | √ | √ |  |  | √ |  | √ |  |
| Tang 2014 |  |  |  |  |  |  | √ |  |
| Emara 2014 |  | √ |  |  |  |  |  |  |
| Machin 2014 |  | √ |  |  |  |  |  |  |
| Wei 2014 | √ | √ |  |  | √ |  | √ |  |
| Han 2014 |  |  |  |  |  |  | √ |  |
| Maniar 2014 |  |  |  |  |  |  | √ |  |
| Wind 2014 |  | √ |  |  |  |  |  |  |
| Huang 2014 | √ | √ |  | √ |  | √ |  | √ |
| Hegde 2013 |  | √ |  |  |  |  |  |  |
| Pispati 2013 |  | √ |  |  |  |  |  |  |
| Alipour 2013 |  |  | √ |  |  |  |  |  |
| Seo 2013 | √ | √ |  |  | √ |  | √ |  |
| Maniar 2012 | √ | √ |  |  | √ |  |  |  |
| Bradshaw 2012 |  |  | √ |  |  |  |  |  |
| Aguilera 2012 |  |  |  |  | √ |  |  |  |
| Zohar 2004 |  |  | √ |  |  |  |  |  |
| Total | 26 | 36 | 6 | 5 | 22 | 6 | 20 | 5 |
| Grand total (N) | 126 | | | | | | | |
| Real total (r) | 53 | | | | | | | |
| No. of meta (c) | 8 | | | | | | | |
| CCA | 19.67% | | | | | | | |

Mark Red: Not RCT

CCA =Corrected covered area. Calculation = CCA (%)=N-r/rc-r:

Where N=number of included publications (sum of checked boxes), r = number of rows (primary studies), c =number of columns (number of systematic reviews)

G. Osteology

| Systematic review | Olsen 2015 | Qiu 2019 | Zhao 2019 | Song 2013 |  | Olsen 2015 | Zhao 2019 |
| --- | --- | --- | --- | --- | --- | --- | --- |
| Characteristics | orthognathic surgery | orthognathic surgery | orthognathic surgery | orthognathic surgery |  | orthognathic surgery | orthognathic surgery |
| Including study |  | | | |  |  | |
| Apipan 2017 |  |  | √ |  |  |  | √ |
| Secher 2017 |  |  | √ |  |  |  | √ |
| Christabel 2016 |  |  | √ |  |  |  | √ |
| Eftekharian 2015 | √ | √ | √ |  |  | √ | √ |
| Christabel 2014 | √ | √ |  |  |  | √ |  |
| Sankar 2012 | √ | √ | √ | √ |  | √ | √ |
| Karimi 2012 | √ |  | √ | √ |  | √ | √ |
| Kaewpradub 2011 | √ | √ | √ | √ |  | √ | √ |
| Espitalier 2011 | √ |  |  |  |  | √ |  |
| Rossi 2010 | √ |  |  |  |  | √ |  |
| Tang 2009 | √ |  |  |  |  | √ |  |
| Choi 2009 | √ | √ | √ | √ |  | √ | √ |
| Stewart 2001 | √ |  |  |  |  | √ |  |
| Enlund 1997 | √ |  |  |  |  | √ |  |
| Total | 11 | 6 | 8 | 4 |  | 11 | 8 |
| Grand total (N) | 29 | | | |  | 19 | |
| Real total (r) | 13 | | | |  | 13 | |
| No. of meta (c) | 4 | | | |  | 2 | |
| CCA | 41.00% | | | |  | 46.10% | |

CCA =Corrected covered area. Calculation = CCA (%)=N-r/rc-r:

Where N=number of included publications (sum of checked boxes), r = number of rows (primary studies), c =number of columns (number of systematic reviews)

H. Orthopedics

| Systematic review | Xiao C 2019 | Haj-Younes 2019 | Baskaran 2017 | Zhang P 2017 | Farrow 2016 |
| --- | --- | --- | --- | --- | --- |
| Characteristics | hip fracture surgery | hip fracture surgery | hip fracture surgery | hip fracture surgery | hip fracture surgery |
| Including Study |  | | | | |
| Luo 2019 |  | √ |  |  |  |
| Tian 2018 | √ | √ |  |  |  |
| Haghighi 2017 | √ | √ |  |  |  |
| Watts 2017 | √ | √ |  |  |  |
| Lei 2017 | √ | √ |  |  |  |
| Baruah 2016 | √ | √ |  |  |  |
| Tengberg 2016 | √ | √ | √ | √ | √ |
| Zhu 2015 |  |  |  | √ |  |
| Ji 2015 |  |  |  | √ |  |
| Lee 2015 |  |  | √ |  | √ |
| Shiva 2015 |  |  | √ |  |  |
| Mohib 2015 | √ |  | √ | √ | √ |
| Emara 2014 | √ |  | √ | √ | √ |
| Vijay 2013 | √ | √ | √ |  | √ |
| Wang 2013 |  |  |  | √ |  |
| Zufferery 2010 | √ | √ | √ | √ | √ |
| Sadeghi 2007 | √ | √ | √ | √ | √ |
| Total | 11 | 10 | 8 | 8 | 7 |
| Grand total (N) | 44 | | | | |
| Real total (r) | 17 | | | | |
| No. of meta (c) | 5 | | | | |
| CCA | 39.70% | | | | |

| Systematic review | Xiong 2020 | Hariharan 2019 | Zhang Y 2019 | Hui 2018 | Yuan Q 2017 | Cheriyan 2014 | Zhang F 2014 | Li Z 2013 | Yang 2013 | Zhan F 2021 | Chen J 2021 |
| --- | --- | --- | --- | --- | --- | --- | --- | --- | --- | --- | --- |
| Characteristics | spine surgery | spine surgery | spine surgery | spine surgery | spine surgery | spine surgery | spine surgery | spine surgery | spine surgery | spine surgery | spine surgery |
| Including Study |  | | | | | | | | | | |
| Wang 2019 | √ |  |  |  |  |  |  |  |  |  |  |
| Zheng 2019 | √ |  |  |  |  |  |  |  |  |  |  |
| Mu 2019 | √ |  |  |  |  |  |  |  |  |  | √ |
| Hui 2019 | √ |  |  |  |  |  |  |  |  |  |  |
| Carabini 2018 |  | √ | √ |  |  |  |  |  |  | √ | √ |
| Moslem 2018 |  |  |  |  |  |  |  |  |  |  | √ |
| Goobie 2018 |  |  |  |  |  |  |  |  |  | √ |  |
| Pong 2018 |  | √ |  |  |  |  |  |  |  |  |  |
| Duan 2018 | √ |  |  |  |  |  |  |  |  |  |  |
| Xue 2018 |  |  | √ |  |  |  |  |  |  |  |  |
| Choi 2017 |  | √ | √ | √ |  |  |  |  |  |  |  |
| Yu 2017 |  |  |  | √ |  |  |  |  |  |  |  |
| Meng 2017 | √ |  |  |  |  |  |  |  |  |  |  |
| Geng 2017 |  |  |  | √ |  |  |  |  |  |  |  |
| Kim 2017 |  |  |  |  |  |  |  |  |  |  | √ |
| Shi 2017 |  |  |  |  |  |  |  |  |  |  | √ |
| Colomina 2016 |  |  | √ | √ |  |  |  |  |  | √ | √ |
| Wu 2016 | √ |  |  |  |  |  |  |  |  |  |  |
| Lu 2016 | √ |  |  |  |  |  |  |  |  |  |  |
| Kushioka 2016 |  |  |  | √ |  |  |  |  |  |  |  |
| Sui 2016 |  |  |  | √ | √ |  |  |  |  |  |  |
| Xie 2015 |  | √ |  | √ |  |  |  |  |  |  |  |
| Ng 2015 |  |  |  | √ | √ |  |  |  |  |  |  |
| da Rocha 2015 |  |  |  | √ | √ |  |  |  |  |  |  |
| Naik 2015 |  |  |  | √ |  |  |  |  |  |  |  |
| Berney 2015 |  |  |  | √ | √ |  |  |  |  |  |  |
| Raksakietisak 2015 |  |  | √ | √ |  |  |  |  |  | √ | √ |
| Peter 2015 |  | √ | √ | √ |  |  |  |  |  |  |  |
| Verma 2014 |  |  |  | √ | √ |  |  |  |  | √ |  |
| Wang 2013 |  |  | √ | √ |  | √ |  |  |  | √ |  |
| Lykissas 2013 |  |  |  | √ | √ |  |  |  |  |  |  |
| Khurana 2012 |  | √ | √ | √ |  |  |  |  |  |  |  |
| Yagi 2012 |  |  |  | √ | √ |  |  |  |  |  |  |
| Newton 2012 |  |  |  | √ |  |  |  |  |  |  |  |
| Dhawale 2012 |  |  |  | √ |  |  |  |  |  |  |  |
| Xu 2012 |  |  |  | √ | √ | √ |  |  |  |  |  |
| Farrokhi 2011 |  |  | √ | √ |  | √ | √ | √ | √ | √ | √ |
| Hang C 2011 |  |  |  |  |  |  |  |  | √ |  |  |
| Endres 2011 |  |  | √ | √ |  |  |  |  |  |  |  |
| Tsutsumimoto 2011 |  |  |  | √ |  | √ | √ | √ | √ | √ |  |
| Huang 2011 |  |  |  | √ |  | √ |  |  |  |  |  |
| Suksamosorn 2011 |  |  |  | √ |  | √ |  |  |  |  |  |
| Baldus 2010 |  | √ | √ | √ |  |  |  |  |  |  |  |
| Taghaddomi 2009 |  |  |  | √ |  |  |  |  |  |  |  |
| Jalaeian 2009a |  |  |  |  |  |  |  |  | √ |  |  |
| Elwatidy 2008 |  |  |  | √ |  | √ | √ | √ | √ |  | √ |
| Wong 2008 |  |  |  | √ |  | √ | √ | √ |  |  | √ |
| Shapiro 2007 |  |  |  | √ |  |  |  |  |  |  |  |
| Bednar 2006 |  |  |  | √ |  |  |  |  |  |  |  |
| Haghighi 2006 |  |  |  | √ |  |  |  |  |  |  |  |
| Sethna 2005 |  |  |  | √ | √ | √ | √ | √ | √ |  |  |
| Wong 2005 |  |  |  |  |  |  |  |  | √ |  |  |
| Neilipovitz 2001 |  |  |  | √ | √ | √ | √ | √ | √ | √ |  |
| Kim 2000 |  |  |  |  |  | √ |  |  | √ |  |  |
| Total | 8 | 7 | 11 | 36 | 10 | 11 | 6 | 6 | 9 | 9 | 10 |
| Grand total (N) | 123 | | | | | | | | | | |
| Real total (r) | 54 | | | | | | | | | | |
| No. of meta (c) | 11 | | | | | | | | | | |
| CCA | 12.70% | | | | | | | | | | |

| Systematic review | Xiong 2020 | Chen J 2021 |
| --- | --- | --- |
| Characteristics | spine surgery | spine surgery |
| Including Study |  | |
| Wang 2019 | √ |  |
| Zheng 2019 | √ |  |
| Mu 2019 | √ | √ |
| Hui 2019 | √ |  |
| Carabini 2018 |  | √ |
| Moslem 2018 |  | √ |
| Duan 2018 | √ |  |
| Meng 2017 | √ |  |
| Colomina 2016 |  | √ |
| Kim 2017 |  | √ |
| Wu 2016 | √ |  |
| Lu 2016 | √ |  |
| Shi 2017 |  | √ |
| Raksakietisak 2015 |  | √ |
| Farrokhi 2011 |  | √ |
| Taghaddomi 2009 |  | √ |
| Elwatidy 2008 |  | √ |
| Total | 8 | 10 |
| Grand total (N) | 18 | |
| Real total (r) | 17 | |
| No. of meta (c) | 2 | |
| CCA | 0.05% | |

| Systematic review | Kuo 2018 | He J 2017 | Kirsch 2017 | Yu B 2017 | Sun C 2017 |
| --- | --- | --- | --- | --- | --- |
| Characteristics | total shoulder arthroplasty | total shoulder arthroplasty | total shoulder arthroplasty | total shoulder arthroplasty | total shoulder arthroplasty |
| Including Study |  | | | | |
| Pauzenberger 2017 | √ |  | √ |  |  |
| Kim 2017 | √ |  |  |  |  |
| Vara 2017 | √ | √ | √ | √ | √ |
| Abildgaard 2016 | √ | √ | √ | √ | √ |
| Friedman 2016 | √ | √ | √ | √ | √ |
| Gillespie 2015 | √ | √ | √ | √ | √ |
| Total | 6 | 4 | 5 | 4 | 4 |
| Grand total (N) | 23 | | | | |
| Real total (r) | 6 | | | | |
| No. of meta (c) | 5 | | | | |
| CCA | 70.80% | | | | |

| Systematic review | Bai 2019 | Du 2018 | Gong 2018 |
| --- | --- | --- | --- |
| Characteristics | spinal fusion surgery | spinal fusion surgery | spinal fusion surgery |
| Including Study |  | | |
| Ou 2018 | √ |  | √ |
| Nagabhushan 2018 |  | √ |  |
| Roopa 2017 | √ |  | √ |
| Kim 2017 | √ | √ | √ |
| Shi 2017 | √ | √ | √ |
| Xu 2017 |  | √ |  |
| Ren 2017 | √ |  |  |
| Liang 2016 | √ | √ |  |
| Kushioka 2016 | √ |  | √ |
| Wang 2013 | √ | √ | √ |
| Endres 2011 | √ |  | √ |
| Total | 9 | 6 | 7 |
| Grand total (N) | 22 | | |
| Real total (r) | 11 | | |
| No. of meta (c) | 3 | | |
| CCA | 50.0% | | |

| Systematic review | Luo 2019 | Jiang 2018 | Zhu 2018 | Wang W 2017 | Zhou X 2019 | Zhong Y 2019 |
| --- | --- | --- | --- | --- | --- | --- |
| Characteristics | Intertrochanteric fracture surgery | Intertrochanteric fracture surgery | Intertrochanteric fracture surgery | Intertrochanteric fracture surgery | Intertrochanteric fracture surgery | Intertrochanteric fracture surgery |
| Including Study |  | | | | | |
| Luo 2019 | √ |  |  |  |  |  |
| Tian 2018 | √ |  | √ |  | √ | √ |
| Xia 2018 |  |  |  |  |  | √ |
| Schiavone 2018 |  |  |  |  | √ | √ |
| Jin 2018 |  |  |  |  |  | √ |
| Lei 2017 | √ | √ | √ | √ | √ | √ |
| Wang 2017 |  | √ |  |  |  | √ |
| Cheng 2017 |  |  |  |  |  | √ |
| Virani 2016 |  | √ | √ | √ | √ | √ |
| Baruah 2016 |  |  | √ |  | √ | √ |
| Athanasios 2016 |  |  |  | √ |  |  |
| Tengberg 2016 | √ |  | √ |  | √ | √ |
| Drakos 2016 | √ | √ | √ |  | √ | √ |
| Lin 2016 |  |  |  |  |  | √ |
| Mohib 2015 |  | √ | √ | √ | √ | √ |
| Total | 5 | 5 | 7 | 4 | 8 | 13 |
| Grand total (N) | 42 | | | | | |
| Real total (r) | 15 | | | | | |
| No. of meta (c) | 6 | | | | | |
| CCA | 36.0% | | | | | |

Mark Red: Not RCT

CCA =Corrected covered area. Calculation = CCA (%)=N-r/rc-r:

Where N=number of included publications (sum of checked boxes), r = number of rows (primary studies), c =number of columns (number of systematic reviews)

I. Antifibrinolytic agents

| Systematic review | Karimi 2019 | Lu 2018 | Li G 2017 | Wang 2015 |
| --- | --- | --- | --- | --- |
| Characteristics | spine surgery | spine surgery | spine surgery | spine surgery |
| Including Study |  | | | |
| Colomina 2017 |  | √ |  |  |
| Shi 2017 |  | √ |  |  |
| Peter 2015 |  | √ | √ |  |
| Raksakietisak 2015 |  | √ | √ |  |
| Verma 2014 | √ |  | √ | √ |
| Wang 2013 |  | √ | √ |  |
| Lykissas 2013 |  |  |  | √ |
| Lorio 2013 |  |  |  | √ |
| Newton 2012 |  |  |  | √ |
| Khurana 2012 |  |  |  | √ |
| Yagi 2012 |  |  |  | √ |
| Xu 2012 | √ |  | √ | √ |
| Farrokhi 2011 |  | √ | √ |  |
| Tsutsumimoto 2011 |  | √ | √ |  |
| Berenholtz 2009 |  | √ | √ |  |
| Elwatidy 2008 |  | √ | √ |  |
| Wong 2008 |  | √ | √ |  |
| Tayyab 2008 |  |  |  | √ |
| Kasimian 2008 |  |  |  | √ |
| Thompson 2008 |  |  |  | √ |
| Sethna 2005 | √ |  | √ | √ |
| Florentino 2004 | √ |  |  |  |
| Pineda 2004 |  |  | √ | √ |
| Khoshlial 2003 |  |  | √ | √ |
| Cole 2003 |  |  | √ | √ |
| Karapurkar 2002 |  |  | √ | √ |
| Neilipovitz 2001 | √ |  | √ | √ |
| Urban 2001 |  | √ |  |  |
| Pineda 2001 |  |  |  | √ |
| Lentschener 1999 |  |  | √ | √ |
| Total | 5 | 11 | 17 | 18 |
| Grand total (N) | 51 | | | |
| Real total (r) | 30 | | | |
| No. of meta (c) | 4 | | | |
| CCA | 23.30% | | | |

| Systematic review | Meybohm 2013 | Schouten 2009 | Abrishami 2009 | Takagi 2008 | Carless 2005 |
| --- | --- | --- | --- | --- | --- |
| Characteristics | cardiac surgery | cardiac surgery | cardiac surgery | cardiac surgery | cardiac surgery |
| Including Study |  | | | | |
| DeSantis 2011 | √ |  |  |  |  |
| Karkouti 2010 | √ |  |  |  |  |
| Sniecinski 2010 | √ |  |  |  |  |
| Sander 2010 | √ |  |  |  |  |
| Stamou 2009 | √ |  |  |  |  |
| Later 2009 | √ |  |  |  |  |
| Waldow 2009 | √ |  |  |  |  |
| Greilich 2009 | √ |  |  |  |  |
| Jakobsen 2009 | √ |  |  |  |  |
| Lindvall 2008 | √ |  |  |  |  |
| Martin 2008 | √ |  |  |  |  |
| Shaw 2008 | √ |  |  |  |  |
| Schneeweiss 2008 | √ |  |  |  |  |
| Wagener 2008 | √ |  |  |  |  |
| Maslow 2008 | √ |  |  |  |  |
| Dietrich 2008 | √ |  |  |  |  |
| Fergusson 2008 | √ |  |  | √ |  |
| Baric 2007 |  |  | √ |  |  |
| Kristeller 2007 | √ |  |  |  |  |
| Mangano 2006 | √ |  |  |  |  |
| Abul-Azm 2006 |  |  | √ |  |  |
| Diprose 2005 | √ |  |  | √ |  |
| Kuitunen 2005 | √ |  |  | √ |  |
| Bulutcu 2005 |  | √ |  |  |  |
| Yasim 2005 |  |  | √ |  |  |
| Hekmat 2004 | √ |  |  |  |  |
| Chauhan 2004 |  | √ |  |  |  |
| Chauhan 2003 |  | √ |  |  |  |
| Mossinger 2003 |  | √ |  |  |  |
| Isgro 2002 |  |  | √ |  |  |
| Greilich 2001 |  |  |  |  | √ |
| Ray 2001 |  |  |  |  | √ |
| De Bonis 2000 |  |  | √ |  |  |
| Chauhan 2000 |  | √ |  |  |  |
| Levin 2000 |  | √ |  |  |  |
| Nuttall 2000 | √ |  |  | √ | √ |
| Maineri 2000 |  |  |  |  | √ |
| Wong 2000 | √ |  |  | √ | √ |
| Rao 2000 |  | √ |  |  |  |
| Casati 2000 | √ |  |  | √ | √ |
| Mand ak 1999 |  |  | √ |  |  |
| Casati 1999 | √ |  |  | √ | √ |
| Bernet 1999 | √ |  |  |  | √ |
| Miller 1998 |  | √ |  |  |  |
| Mongan 1998 | √ |  |  | √ | √ |
| Eberle 1998 |  |  |  |  | √ |
| Misfeld 1998 | √ |  |  |  | √ |
| Hardy 1998 |  |  |  |  | √ |
| Coniff 1998 |  | √ |  |  |  |
| Davies 1997 |  | √ |  |  |  |
| Pinosky 1997 |  |  |  |  | √ |
| Landmore 1997 | √ |  |  |  |  |
| Reid 1997 |  | √ |  |  |  |
| Seghaye 1996 |  | √ |  |  |  |
| Zonis 1996 |  | √ |  |  |  |
| Menichetti 1996 |  |  |  |  | √ |
| D Erricio 1996 |  | √ |  |  |  |
| Penta 1995 |  |  |  |  | √ |
| Corbeau 1995 |  |  |  |  | √ |
| Pugh 1995 |  |  |  |  | √ |
| Speekenbrink 1995 |  |  |  |  | √ |
| Gomar 1995 |  | √ |  |  |  |
| Boldt 1994 |  | √ |  |  |  |
| O regan 1994 |  |  | √ |  |  |
| Blauhut 1994 | √ |  |  | √ | √ |
| Herynkopf 1994 |  | √ |  |  |  |
| Huang 1993 |  | √ |  |  |  |
| Isetta 1993 |  |  |  |  | √ |
| Tatar 1993 |  |  | √ |  |  |
| Dietrich 1993 |  | √ |  |  |  |
| Boldt 1993 |  | √ |  |  |  |
| McClure 1974 |  | √ |  |  |  |
| Total | 31 | 21 | 8 | 9 | 20 |
| Grand total (N) | 89 | | | | |
| Real total (r) | 72 | | | | |
| No. of meta (c) | 5 | | | | |
| CCA | 5.90% | | | | |

Mark Red: Not RCT

CCA =Corrected covered area. Calculation = CCA (%)=N-r/rc-r:

Where N=number of included publications (sum of checked boxes), r = number of rows (primary studies

## Appendix 6. Characteristics of studies included in systematic review

| Author Year | Agent | Surgery | Timing | Administration | Including studies | Amount | Follow-up | Funding | AMSTAR2 rate |
| --- | --- | --- | --- | --- | --- | --- | --- | --- | --- |
| Zhao 2019 ^[1]^ | IV 10-20mg/kg | orthognathic surgery | Perioperative | intravenous vs control | RCT(8) | 575 | not reported | Natural Science Foundation of Shandong Province | High |
| Huang 2014 ^[2]^ | Inconsistent | major orthopedic surgery | Perioperative | intravenous or topical or oral vs control | RCT(46) | 2925 | not reported | Anhui Medical University | High |
| Guo 2018 ^[3]^ | Oral 1g | TKA | postoperative | oral vs control | RCT(5) | 608 | 0-180days | None | High |
| Wu 2017 ^[4]^ | IV 10 mg/kg | TKA | perioperative | intravenous vs control | RCT(6) | 394 | not reported | National Natural Science Foundation of China | High |
| Chen 2014 ^[5]^ | Topical 0.5-3g | TKA | in-operative | topical vs control | RCT(6) | 647 | not reported | None | High |
| Li 2020 ^[6]^ | Inconsistent | TKA | perioperative | intravenous vs topical | RCT(34) | 3867 | 6mouths | None | High |
| Sukeik 2019 ^[7]^ | IV 10-15mg/kg | THA | Perioperative | intravenous vs control | RCT(25) | 1608 | not reported | None | High |
| Zhang H 2017 ^[8]^ | IV 15mg/kg | THA | perioperative | combined intravenous and topical vs intravenous only | RCT(8) | 850 | not reported | National Natural Science Foundation of Hainan Province, Funds for the Associated Committee of Hainan Social Science | High |
| Huang 2015 ^[9]^ | Inconsistent^#^ | THA | perioperative | intravenous or topical vs control | RCT(28) | 2131 | not reported | None | High |
| Ye 2020 ^[10]^ | Inconsistent | TKA&THA | Perioperative | oral vs intravenous | RCT(10) | 1140 | long-term | None | High |
| Sun 2019 ^[11]^ | 1-1.5g IV +  1-3g topical | TKA&THA | Perioperative | intravenous vs topical, combined intravenous and topical vs intravenous or topical only | RCT(26) | 1912 | not reported | None | High |
| Chen 2016 ^[12]^ | Inconsistent | TKA&THA | perioperative | topical vs intravenous | RCT(20) | 1800 | not reported | None | High |
| Kirsch 2017 ^[13]^ | IV 10-20mg/kg | total shoulder arthroplasty | Perioperative | intravenous or topical vs control | RCT(3),cohort studies(2) | 629 | not reported | None | High |
| Zhang XQ 2017 ^[14]^ | IV 15mg/kg | total joint arthroplasty | perioperative | combined intravenous and topical vs intravenous only | RCT(7) | 683 | not reported | None | High |
| Xiong 2020 ^[15]^ | IV 10-15mg/kg | spine surgery | Perioperative | intravenous vs topical | RCT(8) | 660 | not reported | None | High |
| Li G 2017 ^[16]^ | Inconsistent^#^ | spine surgery | perioperative | intravenous vs control | RCT(17) | 1191 | not reported | None | High |
| Chen 2021 ^[17]^ | IV 5-30mg/kg | spine surgery | perioperative | intravenous vs control | RCT(10) | 802 | not reported | the Scientific Research Fund of Sichuan Health Commission | High |
| Du 2018 ^[18]^ | IV 10-30mg/kg | spinal fusion surgery | Perioperative | intravenous vs control | RCT(6) | 394 | not reported | None | High |
| Luo 2020 ^[19]^ | Topical 1-3g | intertrochanteric fracture | Perioperative | intravenous vs control | RCT(5) | 540 | not reported | None | High |
| Zhou 2019 ^[20]^ | 4g | intertrochanteric fracture | perioperative | intravenous or topical vs control | RCT(8) | 836 | not reported | None | High |
| Zhang 2019 ^[21]^ | IV 10-15mg/kg | calcaneal fracture surgery | perioperative | intravenous vs control | RCT(7) | 469 | not reported | None | High |
| Xiao 2019 ^[22]^ | IV 10-15mg/kg | hip fracture surgery | Perioperative | intravenous vs control | RCT(11) | 892 | not reported | None | High |
| Yao 2019 ^[23]^ | Inconsistent | periacetabular osteotomy and high tibial osteotomy | Perioperative | intravenous or topical vs control | RCT(6) | 665 | 30 days | None | High |
| Zhang 2019 ^[24]^ | IV 10-20mg/kg | cardiac surgery | Perioperative | intravenous vs control | RCT(28) | 7446 | not reported | National Natural Science Foundation of China | High |
| Khaie 2019 ^[25]^ | Inconsistent | cardiac surgery | Perioperative | intravenous vs control | RCT(70) | not reported | One mouth or greater | None | High |
| Guo 2019 ^[26]^ | Inconsistent | cardiac surgery | Perioperative | intravenous or topical vs control | RCT(49) | 10591 | not reported | None | High |
| July 2020 ^[27]^ | IV 1g | traumatic brain injury | Perioperative | intravenous vs control | RCT(7) | 34132 | not reported | None | High |
| Hu 2019 ^[28]^ | Inconsistent | cerebral hemorrhage | Perioperative | intravenous vs control | RCT(14) | 4703 | 0-180days | None | High |
| Lu 2019 ^[29]^ | IV 10-50mg/kg | craniosynostosis open surgery | Perioperative | intravenous vs control | RCT(2) | 542 | not reported | None | Moderate |
| Bryant-Smith 2018 ^[30]^ | Oral 3g-4g | heavy menstrual bleeding | periodic | oral vs control | RCT(13) | 1312 | 3-6mouths | None | High |
| Tsai 2020 ^[31]^ | IV 500mg-1g | hemoptysis | Periodic | intravenous vs control | RCT(4) | 183 | not reported | None | High |
| Twum-Barimah 2020 ^[32]^ | Inconsistent | upper gastrointestinal bleeding | Periodic | intravenous or topical or oral vs control | RCT(11) | 2076 | not reported | None | High |
| Juliana 2016 ^[33]^ | Topical 0.5g | minor oral surgery | at least 48 h after surgery | topical vs control | RCT(5) | 252 | 0-7days | None | High |
| Chan 2013 ^[34]^ | IV 10mg/kg | tonsillectomy | preoperatively, perioperatively or postoperatively) | intravenous or topical or oral vs control | RCT(3) case-control studies(4) | 2444 | not reported | None | High |
| Longo 2018 ^[35]^ | IV 10-15mg/kg | prostate surgery | Perioperative | intravenous or oral or local spray vs control | RCT(9) | 978 | not reported | None | High |
| Ker 2015 ^[36]^ | Inconsistent^#^ | acute traumatic injury | perioperative | intravenous vs control | RCT(3) | 40739 | not reported | the National Institute for Health  Research | High |
| Montroy 2017 ^[37]^ | Inconsistent^#^ | cancer | perioperative | intravenous or topical vs control | RCT(11) | 1177 | not reported | None | High |
| Kang 2020 ^[38]^ | IV 1-2g | Endoscopic sinus surgery | Perioperative | topical vs control | RCT(4) | 226 | not reported | National Research Foundation of Korea, funded by the Ministry of Education | Moderate |
| Kim 2019 ^[39]^ | IV 10-15mg/kg | endoscopic sinus surgery | Perioperative | intravenous vs control | RCT(7) | 562 | not reported | National Research Foundation of Korea, funded by the Ministry of Education | High |
| Juliana 2018 ^[40]^ | Inconsistent | rhinoplast | Perioperative | oral or intravenous vs control | RCT(5) | 276 | 1day to 7days | None | High |
| Joseph 2018 ^[41]^ | Topical 400mg | epistaxis | not reported | topical or oral vs control | RCT(6) | 692 | 10mins to 3weeks | National Institute for Health Research, via Cochrane Infrastructure | High |
| Wang 2019 ^[42]^ | IV 10mg/kg | cesarean section | Preoperative | intravenous vs control | RCT(21) | 3852 | not reported | None | High |
| Xia 2020 ^[43]^ | IV 1g | vaginal delivery | After deliver the anterior shoulder | intravenous vs control | RCT(4) | 4579 | not reported | None | High |
| Taeuber 2021 ^[44]^ | Inconsistent | any medical disciplines | Preoperative | intravenous vs control | RCT(216) | 125550 | not reported | None | High |
| Heyns 2021 ^[45]^ | IV 10-20mg/kg | types of surgeries | Preoperative | intravenous vs control | RCT(57) | 5698 | not reported | None | High |
| Murao 2021 ^[46]^ | Inconsistent | types of surgeries | not reported | intravenous vs control | RCT(234) | 102681 | not reported | None | High |
| Zufferey 2021 ^[47]^ | IV 5.5mg/kg to 20 g | Cardiac surgery | Perioperative | intravenous vs intravenous(different dosage) | RCT(64) | 12378 | not reported | None | High |

KIU= Kallikrein Inhibitor Units

^#^Included some data of EACA and aprotinin.

Reference

1. Zhao H, Liu S, Wu Z, Zhao H, Ma C. (2019) Comprehensive assessment of tranexamic acid during orthognathic surgery: A systematic review and meta-analysis of randomized, controlled trials. J Craniomaxillofac Surg. 47(4):592-601. doi: 10.1016/j.jcms.2019.01.021

2. Huang F, Wu D, Ma G, Yin Z, Wang Q. (2014) The use of tranexamic acid to reduce blood loss and transfusion in major orthopedic surgery: a meta-analysis. J Surg Res. 186(1):318-327. doi: 10.1016/j.jss.2013.08.020

3. Guo P, He Z, Wang Y, Gao F, Sun W, Guo W, et al. (2018) Efficacy and safety of oral tranexamic acid in total knee arthroplasty: A systematic review and meta-analysis. Medicine (Baltimore). 97(18):e0587. doi: 10.1097/MD.0000000000010587

4. Wu Y, Yang T, Zeng Y, Si H, Cao F, Shen B. (2017) Tranexamic acid reduces blood loss and transfusion requirements in primary simultaneous bilateral total knee arthroplasty: a meta-analysis of randomized controlled trials. Blood Coagul Fibrinolysis. 28(7):501-508. doi: 10.1097/MBC.0000000000000637

5. Chen ZY, Gao Y, Chen W, Liu YJ, Zhang YZ. (2014) Reduced blood loss after intra-articular tranexamic acid injection during total knee arthroplasty: a meta-analysis of the literature. Knee Surg Sports Traumatol Arthrosc. 22(12):3181-3190. doi: 10.1007/s00167-013-2814-3

6. Li J, Liu R, Rai S, Ze R, Tang X, Hong P. (2020) Intra-articular vs. intravenous administration: a meta-analysis of tranexamic acid in primary total knee arthroplasty. J Orthop Surg Res. 2;15(1):581. doi: 10.1186/s13018-020-02119-1

7. Sukeik M, Alshryda S, Powell J, Haddad FS. (2020) The effect of tranexamic acid on wound complications in primary total Hip Arthroplasty: A meta-analysis. Surgeon. 18(1):53-61. doi: 10.1016/j.surge.2019.05.003

8. Zhang H, He G, Zhang C, Xu B, Wang X, Zhang C. (2017) Is combined topical and intravenous tranexamic acid superior to intravenous tranexamic acid alone for controlling blood loss after total hip arthroplasty?: A meta-analysis. Medicine (Baltimore). 96(21):e6916. doi: 10.1097/MD.0000000000006916

9. Huang F, Wu Y, Yin Z, Ma G, Chang J. (2015) A systematic review and meta-analysis of the use of antifibrinolytic agents in total hip arthroplasty. Hip Int. 25(6):502-509. doi: 10.5301/hipint.5000285

10. Ye W, Liu Y, Liu WF, Li XL, Fei Y, Gao X. (2020) Comparison of efficacy and safety between oral and intravenous administration of tranexamic acid for primary total knee/hip replacement: a meta-analysis of randomized controlled trial. J Orthop Surg Res. 15(1):21. doi:10.1186/s13018-019-1528-8

11. Sun Q, Li J, Chen J, Zheng C, Liu C, Jia Y. (2019) Comparison of intravenous, topical or combined routes of tranexamic acid administration in patients undergoing total knee and hip arthroplasty: a meta-analysis of randomised controlled trials. BMJ Open. 9(1):e024350. doi: 10.1136/bmjopen-2018-024350

12. Chen Y, Chen Z, Cui S, Li Z, Yuan Z. (2016) Topical versus systemic tranexamic acid after total knee and hip arthroplasty: A meta-analysis of randomized controlled trials. *Medicine (Baltimore)*. 95(41):e4656. doi:10.1097/MD.0000000000004656

13. Kirsch JM, Bedi A, Horner N, Wiater JM, Pauzenberger L, Koueiter DM, et al. (2017) Tranexamic Acid in Shoulder Arthroplasty: A Systematic Review and Meta-Analysis. JBJS Rev. 5(9):e3. doi: 10.2106/JBJS.RVW.17.00021

14. Zhang XQ, Ni J, Ge WH. (2017) Combined use of intravenous and topical versus intravenous tranexamic acid in primary total joint arthroplasty: A meta-analysis of randomized controlled trials. Int J Surg. 38:15-20. doi: 10.1016/j.ijsu.2016.11.136

15. Xiong Z, Liu J, Yi P, Wang H, Tan M. (2020) Comparison of Intravenous versus Topical Tranexamic Acid in Nondeformity Spine Surgery: A Meta-Analysis. Biomed Res Int. 2020:7403034. doi:10.1155/2020/7403034

16. Li G, Sun TW, Luo G, Zhang C. (2017) Efficacy of antifibrinolytic agents on surgical bleeding and transfusion requirements in spine surgery: a meta-analysis. Eur Spine J. 26(1):140-154. doi:10.1007/s00586-016-4792-x

17. Chen JP, Li K, Chen Q, Guo HR, Zhang YB, Wei B. (2021) Meta-analysis of the efficacy and safety of tranexamic acid in open spinal surgery. Zhongguo Zuzhi Gongcheng Yanjiu. 25(9):1458-1464. doi: 10.3969/j.issn.2095-4344.3765

18. Du Y, Feng C. (2018) The Efficacy of Tranexamic Acid on Blood Loss from Lumbar Spinal Fusion Surgery: A Meta-Analysis of Randomized Controlled Trials. World Neurosurg. 119:e228-234. doi: 10.1016/j.wneu.2018.07.120

19. Luo X, Huang H, Tang X. (2020) Efficacy and safety of tranexamic acid for reducing blood loss in elderly patients with intertrochanteric fracture treated with intramedullary fixation surgery: A meta-analysis of randomized controlled trials. Acta Orthop Traumatol Turc. 54(1):4-14. doi:10.5152/j.aott.2020.01.88

20. Zhou XD, Li J, Fan GM, Huang Y, Xu NW. (2019) Efficacy and safety of tranexamic acid in elderly patients with intertrochanteric fracture: An updated meta-analysis. World J Clin Cases. 7(11):1302-14. doi: 10.12998/wjcc.v7.i11.1302

21. Zhang S, Ge WL, Li C, Han SC, Wang G, Ren WJ. (2019) Efficacy and safety of intravenous tranexamic acid in the operative treatment of calcaneal fractures: a meta-analysis. Zhongguo Zuzhi Gongcheng Yanjiu. 23(20):3268-3274. doi: 10.3969/j.issn.2095-4344.3765

22. Xiao C, Zhang S, Long N, Yu W, Jiang Y. (2019) Is intravenous tranexamic acid effective and safe during hip fracture surgery? An updated meta-analysis of randomized controlled trials. Arch Orthop Trauma Surg. 139(7):893-902. doi:10.1007/s00402-019-03118-6

23. Yao RZ, Gao WQ, Wang BW, Wang GL, Wu CX, A-Mu YD. (2019) Efficacy and Safety of Tranexamic Acid in Reducing Blood Loss of Lower Extremity Osteotomy in Peri-acetabulum and High Tibia: A Systematic Review and Meta-analysis. Orthop Surg. 11(4):545-551. doi: 10.1111/os.12515

24. Zhang Y, Bai Y, Chen M,  Zhou Y, Yu X, Zhou H, et al. (2019) The safety and efficiency of intravenous administration of tranexamic acid in coronary artery bypass grafting (CABG): a meta-analysis of 28 randomized controlled trials. BMC Anesthesiol. 19(1):104. doi: 10.1186/s12871-019-0761-3

25. Khair S, Perelman I, Yates J, Taylor J, Lampron J, Tinmouth A, et al. (2019) Exclusion criteria and adverse events in perioperative trials of tranexamic acid in cardiac surgery: a systematic review and meta-analysis. Can J Anaesth. 66(10):1240-1250. doi: 10.1007/s12630-019-01393-w.

26. Guo J, Gao X, Ma Y, Lv H, Hu W, Zhang S, et al. (2019) Different dose regimes and administration methods of tranexamic acid in cardiac surgery: a meta-analysis of randomized trials. BMC Anesthesiol. 19(1):129. doi: 10.1186/s12871-019-0772-0

27. July J, Pranata R. (2020) Tranexamic acid is associated with reduced mortality, hemorrhagic expansion, and vascular occlusive events in traumatic brain injury - meta-analysis of randomized controlled trials. BMC Neurol. 20(1):119. doi: 10.1186/s12883-020-01694-4

28. Hu W, Xin Y, Chen X, Song Z, He Z, Zhao Y. (2019) Tranexamic Acid in Cerebral Hemorrhage: A Meta-Analysis and Systematic Review. CNS Drugs. 33(4):327-336. doi: 10.1007/s40263-019-00608-4

29. Lu VM, Goyal A, Daniels DJ. (2019) Tranexamic Acid Decreases Blood Transfusion Burden in Open Craniosynostosis Surgery Without Operative Compromise. J Craniofac Surg. 30(1):120-126. doi: 10.1097/SCS.0000000000004875

30. Bryant-Smith AC, Lethaby A, Farquhar C, Hickey M. (2018) Antifibrinolytics for heavy menstrual bleeding. Cochrane Database Syst Rev. 4(4):CD000249. doi: 10.1002/14651858.CD000249

31. Tsai YS, Hsu LW, Wu MS, Chen KH, Kang YN. (2020) Effects of Tranexamic Acid on Hemoptysis: A Systematic Review and Meta-Analysis of Randomized Controlled Trials. Clin Drug Investig. 40(9):789-797. doi: 10.1007/s40261-020-00946-y

32. Twum-Barimah E, Abdelgadir I, Gordon M, Akobeng AK. (2020) Systematic review with meta-analysis: the efficacy of tranexamic acid in upper gastrointestinal bleeding. Aliment Pharmacol Ther. 51(11):1004-1013. doi: 10.1111/apt.15761

33. de Vasconcellos SJ, de Santana Santos T, Reinheimer DM, Faria-E-Silva AL, de Melo MF, Martins-Filho PR. (2017) Topical application of tranexamic acid in anticoagulated patients undergoing minor oral surgery: A systematic review and meta-analysis of randomized clinical trials. J Craniomaxillofac Surg. 45(1):20-26. doi: 10.1016/j.jcms.2016.10.001

34. Chan CC, Chan YY, Tanweer F. (2013) Systematic review and meta-analysis of the use of tranexamic acid in tonsillectomy. Eur Arch Otorhinolaryngol. 270(2):735-748. doi: 10.1007/s00405-012-2184-3

35. Longo MA, Cavalheiro BT, de Oliveira Filho GR. (2018) Systematic review and meta-analyses of tranexamic acid use for bleeding reduction in prostate surgery. J Clin Anesth. 48:32-38. doi: 10.1016/j.jclinane.2018.04.014

36. Ker K, Roberts I, Shakur H, Coats TJ. (2015) Antifibrinolytic drugs for acute traumatic injury. Cochrane Database Syst Rev. (5):CD004896. doi:10.1002/14651858.CD004896.pub4

37. Montroy J, Fergusson NA, Hutton B, Lavallée LT, Morash C, Cagiannos I,et al. (2017) The Safety and Efficacy of Lysine Analogues in Cancer Patients: A Systematic Review and Meta-Analysis. Transfus Med Rev. 31(3):141-148. doi: 10.1016/j.tmrv.2017.03.002.

38. Kang H, Hwang SH. (2020) Does topical application of tranexamic acid reduce intraoperative bleeding in sinus surgery during general anesthesia? Braz J Otorhinolaryngol. 86(1):111-118. doi: 10.1016/j.bjorl.2019.08.006

39. Kim DH, Kim S, Kang H, Jin HJ, Hwang SH. (2019) Efficacy of tranexamic acid on operative bleeding in endoscopic sinus surgery: A meta-analysis and systematic review. Laryngoscope. 129(4):800-807. doi: 10.1002/lary.27766

40. de Vasconcellos SJA, do Nascimento-Júnior EM, de Aguiar Menezes MV, Tavares Mendes ML, de Souza Dantas R, Martins-Filho PRS. (2018) Preoperative Tranexamic Acid for Treatment of Bleeding, Edema, and Ecchymosis in Patients Undergoing Rhinoplasty: A Systematic Review and Meta-analysis. JAMA Otolaryngol Head Neck Surg. 144(9):816-823. doi: 10.1001/jamaoto.2018.1381

41. Joseph J, Martinez-Devesa P, Bellorini J, urton MJ. (2018) Tranexamic acid for patients with nasal haemorrhage (epistaxis). Cochrane Database Syst Rev. 12(12):CD004328. doi: 10.1002/14651858.CD004328

42. Wang Y, Liu S, He L. (2019) Prophylactic use of tranexamic acid reduces blood loss and transfusion requirements in patients undergoing cesarean section: A meta-analysis. J Obstet Gynaecol Res. 45(8):1562-1575. doi: 10.1111/jog.14013

43. Xia Y, Griffiths BB, Xue Q. (2020) Tranexamic acid for postpartum hemorrhage prevention in vaginal delivery: A meta-analysis. Medicine (Baltimore). 99(3):e18792. doi: 10.1097/MD.0000000000018792

44. Taeuber I, Weibel S, Herrmann E, Neef V, Schlesinger T, Kranke P, et al. (2021) Association of Intravenous Tranexamic Acid With Thromboembolic Events and Mortality: A Systematic Review, Meta-analysis, and Meta-regression. JAMA Surg. 156(6):e210884. doi: 10.1001/jamasurg.2021.0884

45. Heyns M, Knight P, Steve AK, Yeung JK. (2021) A Single Preoperative Dose of Tranexamic Acid Reduces Perioperative Blood Loss: A Meta-analysis. Ann Surg. 273(1):75-81. doi: 10.1097/SLA.0000000000003793

46. Murao S, Nakata H, Roberts I, Yamakawa K. (2021) Effect of tranexamic acid on thrombotic events and seizures in bleeding patients: a systematic review and meta-analysis. Crit Care. 25(1):380. doi: 10.1186/s13054-021-03799-9

47. Zufferey PJ, Lanoiselée J, Graouch B, Vieille B, Delavenne X, Ollier E. (2021) Exposure-Response Relationship of Tranexamic Acid in Cardiac Surgery. Anesthesiology. 134(2):165-178. doi: 10.1097/ALN.0000000000003633

## Appendix 7. List of excluded studies and reasons for their exclusion

| Author Year | Agent | Surgery | Reasons for exclusion |
| --- | --- | --- | --- |
| Yates 2018 | TXA | all kinds of surgery | data deficiencies |
| Ma 2011 | TXA | off-pump coronary artery bypass grafting surgery surgery | sample size is too small |
| Dai 2017 | TXA | off-pump coronary artery bypass grafting surgery | Incomplete type of operation |
| Habbab 2019 | TXA | cardiac surgery | bad AMASAR |
| Takagi 2017 | TXA | cardiac surgery | sample size is too small |
| Faraoni 2012 | TXA | cardiac surgery | sample size is too small |
| Zehtabchi 2016 | TXA | traumatic brain injury | sample size is too small |
| Weng 2018 | TXA | traumatic brain injury | sample size is too small |
| Huang 2018 | TXA | cerebral hemorrhage | sample size is too small and bad AMSTAR |
| Qiu 2019 | TXA | orthognathic surgery | sample size is small |
| Song 2013 | TXA | orthognathic surgery | sample size is too small |
| Olsen 2015 | TXA, aprotinin, Yunnan Baiyao, ropivacaine | orthognathic surgery | Not update |
| Gao 2020 | TXA | intracranial hematoma Traumatic Brain Injury | sample size is too small |
| Zhang 2017 | TXA | total knee arthroplasty | Not all studies are RCT |
| Liao 2018 | TXA | total knee arthroplasty | Wrong experimental group |
| Han Y 2018 | TXA | total knee arthroplasty | Wrong experimental group |
| Zhang Y 2017 | TXA | total knee arthroplasty | Wrong experimental group |
| Chen TP 2017 | TXA | total knee arthroplasty | bad AMSTAR |
| Yu 2015 | TXA | total knee arthroplasty | bad AMSTAR |
| Chen X 2015 | TXA | total knee arthroplasty | bad AMSTAR |
| Fu 2013 | TXA | total knee arthroplasty | bad AMSTAR |
| Yang 2012 | TXA | total knee arthroplasty | bad AMSTAR |
| Cid 2005 | TXA | total knee arthroplasty | bad AMSTAR |
| Chen 2015 | TXA | total knee arthroplasty | bad AMSTAR |
| Tian 2017 | TXA | (revision) total knee arthroplasty | bad AMSTAR |
| Tan 2013 | TXA | total knee arthroplasty | Old studies (only include sutdies 10 years ago) |
| Panteli 2013 | TXA | total knee arthroplasty | Not update |
| Moskal 2017 | TXA | (primary, unilateral) total knee arthroplasty | bad AMSTAR |
| Zhang 2014 | TXA | total knee arthroplasty | bad AMSTAR |
| Wang 2014 | TXA | total knee arthroplasty | Not update |
| Wang 2017 | TXA | total knee arthroplasty | bad AMSTAR |
| Gao 2016 | Fibrin sealant(FS), TXA | total knee arthroplasty | bad AMSTAR and not all study are RCT |
| Lin 2016 | TXA | total knee arthroplasty | Not update |
| Fu 2016 | TXA | total knee arthroplasty | Not update |
| Meena 2017 | TXA | total knee arthroplasty | Not update |
| Mi B 2017 | TXA | total knee arthroplasty | bad AMSTAR |
| Shemshaki 2015 | TXA | total knee arthroplasty | sample size is small than the other one |
| Wen 2016 | TXA | (bilateral) total knee arthroplasty | bad AMSTAR and not all study are RCT |
| Xin 2019 | TXA | total hip arthroplasty | bad AMSTAR and not all study are RCT |
| Zhou 2013 | TXA | total hip arthroplasty | not update |
| Sukeik 2010 | TXA | total hip arthroplasty | not update |
| He 2015 | TXA | (bilateral) total hip arthroplasty | bad AMSTAR |
| Xu 2019 | TXA | total hip arthroplasty | bad AMSTAR |
| Xu 2015 | TXA | total hip arthroplasty | Sample size is too small |
| Wang 2015 | TXA | total hip arthroplasty | bad AMSTAR |
| Wang 2018 | TXA | total hip arthroplasty | not all study are RCT |
| Wu Y 2018 | TXA | total hip arthroplasty | sample size is small than the other one |
| Fillingham 2018 | TXA | total hip arthroplasty | bad AMSTAR |
| Sun 2017 | TXA | total hip arthroplasty | sample size is small than others |
| Liu 2017 | TXA | total hip arthroplasty | bad AMSTAR |
| Huang C 2020 | TXA | total knee arthroplasty | sample size is small than others |
| Dai 2018 | TXA | total knee arthroplasty | sample size is small than others |
| Wu 2015 | TXA | total knee arthroplasty | Old studies (only include sutdies 8 years ago) |
| Yoon 2018 | TXA | total hip arthroplasty | too complicated |
| Zhang P 2017 | TXA | total hip arthroplasty | bad AMSTAR and not all study are RCT |
| Li J 2016 | TXA | total hip arthroplasty | Not all study are RCT |
| Sun 2016 | TXA | total hip arthroplasty | sample size is small and not all study are RCT |
| Chen S 2016 | TXA | total hip arthroplasty | Not all studies are RCT |
| Wang 2019 | TXA | total hip arthroplasty | bad AMASAR |
| Sun 2020 | TXA | total hip arthroplasty and total knee arthroplasty | sample size is small than others |
| Han 2018 | TXA | total hip arthroplasty and total knee arthroplasty | sample size is small than others |
| Chen 2019 | TXA | total hip arthroplasty and total knee arthroplasty | sample size is small than others and and not all study are RCT |
| Arianna 2018 | TXA | total knee arthroplasty and total hip arthroplasty | Bad AMSTAR |
| Zhang 2017 | TXA | total hip arthroplasty and total knee arthroplasty | sample size is small than others and and not all study are RCT |
| Shang 2016 | TXA | total hip arthroplasty and total knee arthroplasty | sample size is small than others |
| Mi 2017 | TXA | total hip arthroplasty and total knee arthroplasty | sample size is small than others |
| Xie 2017 | TXA | total hip arthroplasty and total knee arthroplasty | sample size is small than others |
| Li F 2017 | TXA | total hip arthroplasty and total knee arthroplasty | sample size is too small |
| Xu 2019 | TXA | total hip arthroplasty and total knee arthroplasty | Bad AMSTAR |
| Wei 2014 | TXA | total knee arthroplasty and total hip arthroplasty | Bad AMSTAR |
| HO 2003 | TXA | total knee arthroplasty and total hip arthroplasty | Not update |
| Alshryda 2014 | TXA | total knee arthroplasty and total hip arthroplasty | bad AMASAR |
| Kuo 2018 | TXA | total joint arthroplasty | bad AMSTAR |
| Fillingham 2018 | TXA | total joint arthroplasty | bad AMSTAR |
| Fillingham 2018 | TXA | total joint arthroplasty | bad AMSTAR |
| Li 2017 | TXA | total joint arthroplasty | sample size is too small |
| Alshryda 2011 | TXA | total knee arthroplasty | unable to find all relavent data |
| Zhang P 2017 | TXA | hip fracture | bad AMSTAR |
| Haj-Younes 2019 | TXA | hip fractures surgery | sample size is small than others and and not all study are RCT |
| Farrow 2016 | TXA | hip fracture | not all study are RCT |
| Baskaran 2017 | TXA | hip fracture | bad AMSTAR |
| Zhong 2019 | TXA | adolescent idiopathic scoliosis undergoing corrective surgery | bad AMSTAR |
| Yerneni 2018 | TXA | spine surgery | sample size is small than others and and not all study are RCT |
| Luo W 2018 | TXA | spine surgery | bad AMSTAR |
| Gill 2008 | aprotinin, TXA, and EACA | spine surgery | bad AMSTAR |
| Hariharan 2019 | TXA | adult spinal deformity surgery | sample size is small than others |
| Yuan Q 2017 | TXA | scoliosis surgery | sample size is small than others |
| Zhang Y 2019 | TXA | spine surgery | sample size is small than others |
| Cheriyan 2014 | TXA | spine surgery | Not update |
| Zhang F 2014 | TXA | spine surgery | Not update |
| Li Z 2013 | TXA | spine surgery | Not update |
| Yang 2013 | TXA | spine surgery | Not update |
| Kuo 2018 | TXA | total shoulder arthroplasty | bad AMSTAR |
| He J 2017 | TXA | total shoulder arthroplasty | sample size is small than others |
| Yu B 2017 | TXA | total shoulder arthroplasty | sample size is small than others |
| Sun C 2017 | TXA | total shoulder arthroplasty | sample size is small than others |
| Bai 2019 | TXA | posterior lumbar fusion surgery | not all study are RCT |
| Gong 2018 | TXA | posterior lumbar interbody fusion | not all study are RCT |
| Jiang 2018 | TXA | Intertrochanteric fracture surgery | Not update |
| Zhu 2018 | TXA | intertrochanteric fracture surgery | bad AMSTAR |
| Wang W 2017 | TXA | intertrochanteric fractures | sample size is small than others |
| Hu 2019 | TXA | orthopaedic trauma surgery | data can't be separate clearly |
| Amer 2017 | TXA | fracture repair surgery | data can't be separate clearly |
| Gausden 2017 | TXA | orthopaedic trauma surgery | data can't be separate clearly |
| Karimi 2019 | TXA, EACA | paediatric spine surgery | sample size is small |
| Lu 2018 | TXA, EACA | adult spine surgery | bad AMSTAR |
| Wang 2015 | aprotinin, TXA, EACA | scoliosis surgery | bad AMSTAR and not all study are RCT |
| Henry 2009 | aprotinin, TXA, EACA | cardiac surgery | data deficiencies |
| Carless 2005 | aprotinin, TXA, EACA | ardiac surgery | bad AMSTAR |
| Schouten 2009 | aprotinin, TXA, EACA | cardiac surgery, scoliosis surgery | data can't be separate clearly |
| Meybohm 2013 | aprotinin, TXA, EACA | cardiac surgery | bad AMSTAR and not all study are RCT |
| Liu Q 2018 | TXA, EACA | total knee and total hip arthroplasty | not all study are RCT |
| Kagoma 2008 | aprotinin, TXA, EACA | total hip arthroplasty or total knee arthroplasty | bad AMSTAR |
| Gill 2006 | aprotinin, TXA | total knee arthroplasty | bad AMSTAR |
| Molenaar 2007 | aprotinin, TXA | liver transplantation | bad AMSTAR |
| Ross 2012 | TXA, EACA | spontaneous Bleeding | bad AMSTAR |
| Breau 2014 | PAMBA, TXA, EACA | pelvic surgery | bad AMSTAR |
| Zufferey 2006 | aprotinin, TXA, EACA | orthopedic surgery | data can't be separate clearly |
| Wardrop 2013 | TXA, EACA | aematological disorders | sample size is too small |
| Ker 2013 | TXA | cardiac, orthopaedic, obstetric & gynaecological, head & neck, breast cancer, hepatic and urological | data can't be separate clearly |
| Lin 2016 | TXA | Pulmonary endarterectomy, Cardiac surgery | bad AMSTAR |
| El-Menyar 2018 | TXA | traumatic injury | sample size is too small |
| Chen 2019 | TXA | traumatic brain injury, neurosurgery, extracranial surgery | data can't be separate clearly |
| Franchini 2018 | TXA | major orthopaedic surgery | bad AMSTAR |
| Glass 2016 | TXA | craniofacial pediatric craniosynostosis surgery,neck dissection, oncological tumor resection of the breast | bad AMSTAR |
| Chornenki 2019 | TXA | leukemia-related bleeding GI bleeding, heavy menstrual bleeding, prevention or treatment of post-partum hemorrhage, intracranial bleeding or neurologic injury,  non-specific traumatic injury, hereditary hemorrhagic telangiectasia, and melasma. | bad AMSTAR |
| Riaz 2019 | TXA,EACA | total knee arthroplasty | sample size is too small |
| Ker 2012 | TXA | surgical bleeding | data deficiencies and data can't be separate clearly |
| Manabe 2008 | TXA,aprotinin | cardiac surgery | bad AMSTAR |
| Estcourt 2016 | TXA,EACA | haematological disorders | not meta analysis |
| Tzortzopoulou 2008 | aprotinin, TXA, EACA | scoliosis surgery | sample size is too small |
| Gayet-Ageron 2018 | TXA | acute severe bleeding | sample size is small than others |
| Prutsky 2016 | TXA | haemoptysis | sample size is too small |
| Gluud 2012 | TXA | upper gastrointestinal bleeding | bad AMSTAR |
| Novikova 2011 | TXA | postpartum haemorrhage | sample size is too small |
| Katharine 2013 | TXA | orthognathic surgery | data can't be separate clearly |
| Brown 2007 | aprotinin, TXA, EACA | cardiac surgery | data deficiencies |
| Baharoglu 2013 | TXA, EACA | aneurysmal subarachnoid haemorrhage | data deficiencies |
| Chen R 2019 | EACA | open spinal deformity surgery | not all study are RCT |
| Li L 2019 | EACA | total knee and hip arthroplasty | not all study are RCT |
| Li Y 2018 | EACA | total knee and hip arthroplasty | not all study are RCT |
| Lu J 2015 | EACA | open-heart surgery | sample size is small |
| Dong Q 2018 | EACA | total knee and hip arthroplasty | not all study are RCT |
| Liu C 2008 | aprotinin | orthotopic  liver transplantation | bad AMSTAR |
| Sedrakyan 2004 | aprotinin | artery bypass grafting | bad AMSTAR |
| Munoz 1999 | aprotinin,EACA | cardiac surgery | bad AMSTAR |
| Joshua 2009 | aprotinin | cardiac surgery | data can't be separate clearly |
| Xu J 2019 | aprotinin | total hip arthroplasty | sample size is too small |
| Donald 2005 | aprotinin | cardiac surgery | Not update |
| Burke 2021 | TXA | upper gastrointestinal bleeding | bad AMSTAR |
| Du C 2020 | TXA | traumatic brain injury | bad AMSTAR |
| Lin 2020 | TXA | total knee and hip arthroplasties | bad AMSTAR |
| Lu Z 2019 | TXA | primary unilateral  total hip arthroplasty | bad AMSTAR |
| Sun L 2020 | TXA | primary hip arthroplasty | not all study are RCT |
| Zhang Y 2019 | TXA | total knee arthroplasty | bad AMSTAR |
| Huang L 2020 | TXA | spinal deformity | data deficiencies |
| Teoh 2020 | TXA | surgery | data can't be separate clearly |
| Zhong D 2018 | TXA | thoracolumbar posterior fusion | bad AMSTAR |
| Al-Jeabory 2021 | TXA | Emergency Trauma | not all study are RCT |
| Alhelaly 2019 | TXA | traumatic brain injury | sample size is small than others |
| Yokobori 2020 | TXA | traumatic brain injury | sample size is small than others |
| Hui 2018 | TXA | spine surgery | not all study are RCT |
| Zhan F 2021 | TXA | spinal surgery | sample size is small than others |
| Zhong Y 2019 | TXA | intertrochanteric fracture | not all study are RCT |
| Xiong 2018 | TXA | total knee arthroplasty | sample size is small than others |
| Ageron 2020 | TXA | acute bleeding | bad AMSTAR |
| Sampaio 2019 | Not extracted | Not extracted | not English |
| Seta 2014 | Not extracted | Not extracted | not much about antifibrinolytics |
| Yao Y 2019 | Not extracted | Not extracted | not a common antifibrinolytics |
| Mousa 2017 | Not extracted | postpartum haemorrhage | not much about antifibrinolytics |
| Coats 2008 | Not extracted | Not extracted | sample size is too small |
| Henry 2011 | Not extracted | Not extracted | data can't be separate clearly |
| Perel 2013 | Not extracted | Not extracted | data can't be separate clearly |
| Ray S 2016 | Not extracted | bleeding disorders | not much about antifibrinolytics |
| Martin-Hirsch 2013 | Not extracted | Not extracted | not much about antifibrinolytics |
| Hutton 2018 | Not extracted | Not extracted | data can't be separate clearly |
| Yuan 2019 | aprotinin, EACA,TXA | spine surgery | CCA>10% and relevant lower AMSTAR score |
| Howell 2013 | aprotinin | cardiac surgery | CCA>10% and relevant lower AMSTAR score |
| Brown 2009 | aprotinin | cardiac surgery | CCA>10% and relevant lower AMSTAR score |
| Derzon 2019 | TXA, EACA, and aprotinin | orthopedic and cardiac surgery | CCA>10% and relevant lower AMSTAR score |
| Bridwell 2019 | TXA | Epistaxis | critically low AMSTAR score |
| Ping 2019 | TXA | endoscopic sinus surgery, rhinoplasty, septoplasty | CCA>10% and relevant lower AMSTAR score |
| Pundir 2013 | TXA | endoscopic sinus surgery | CCA>10% and relevant lower AMSTAR score |
| McGuire 2019 | TXA | primary elective rhinoplasty | CCA>10% and same AMSTAR score but fewer outcome data |
| Gottlieb 2019 | TXA | epistaxis | not Cochrane review |
| Franchini 2018 | TXA | cesarean section | CCA>10% and relevant lower quality of analysis |
| Li 2017 | TXA | caesarean section, vaginal delivery | CCA>10% and relevant lower quality of analysis |
| Topsoee 2016 | TXA | cesarean section, myomectomy | CCA>10% and relevant fewer including studies |
| Simonazzi 2016 | TXA | caesarean section | CCA>10% and relevant fewer including studies |
| Alam 2015 | TXA | caesarean section, vaginal delivery | CCA>10% and relevant fewer including studies |
| Wang 2015 | TXA | caesarean section | CCA>10% and relevant fewer including studies |
| Heesen 2014 | TXA | caesarean section, vaginal delivery | CCA>10% and relevant lower AMSTAR score |
| Peitsidis 2011 | TXA | caesarean section | CCA>10% and relevant lower AMSTAR score |
| Corte 2018 | TXA | vaginal delivery | Insufficient meta-analysis data of outcome |
| Fusca 2018 | TXA | myomectomy | Insufficient data of heterogeneity analysis |
| Wu 2018 | TXA,epinephrine | total knee arthroplasty | Wrong experimental group |
| Zhang 2018 | TXA,epinephrine | total hip arthroplasty | Wrong experimental group |
| Yu 2017 | TXA,epinephrine | total knee arthroplasty and total hip arthroplasty | Wrong experimental group |

## Appendix 8. Tabular presentation of outcomes in included Meta-analysis.

A1. joint replacement surgery--TKA

| Dosage | Surgery | Author year | Type of Incl. study | AMSTAR | Outcome | Subgroup | No. of  incl. study | No. of  Patient | Evidence synthesis | I2 | Public-ation bias | GRADE  quality |
| --- | --- | --- | --- | --- | --- | --- | --- | --- | --- | --- | --- | --- |
| 1g Oral | total knee arthroplasty | Guo 2018 | RCT | High | hemoglobin (Hb) drop |  | 3 | 515 | SMD -0.936[-1.118,-0.754] | 0.0% | High | Moderate |
|  |  |  |  |  | hematocrit |  | 2 | 99 | SMD -0.936[-1.113,-0.274] | 0.0% | High | Moderate |
|  |  |  |  |  | drain output |  | 5 | 608 | SMD -0.793[-0.959,-0.628] | 35.6% | High | Moderate |
|  |  |  |  |  | transfusion rate |  | 4 | 555 | RD -0.087[-0.197,0.022] | 87.2% | High | Very low |
|  |  |  |  |  | thromboembolic complications |  | 5 | 608 | RD 0.003[-0.015,0.021] | 0.0% | High | Low |
|  | | | | | | | | | | | | |
| 10mg/kg IV | total knee arthroplasty | Wu 2017 | RCT | High | transfusion requirements |  | 5 | 398 | RR 0.38[0.21,0.68] | 67.0% | High | Moderate |
|  |  |  |  |  | deep venous thrombosis (DVT) |  | 6 | 394 | RR 1.00[0.18,5.42] | 0.0% | Low | Moderate |
|  |  |  |  |  | total blood loss(ml) |  | 4 | 332 | WMD -322.96[-519.52,-126.40] | 89.0% | High | Very low |
|  |  |  |  |  | drainage volume(ml) |  | 4 | 328 | WMD -345.16[-551.76,-138.57] | 95.0% | High | Very low |
|  | | | | | | | | | | | | |
| Inconsistent | total knee arthroplasty | longo 2018 | RCT | High | total blood loss(ml) |  | 4 | 517 | WMD -320.44[-401.20, -239.68] | 89.1% | High | Very low |
|  |  |  |  |  | drainage blood loss(ml) |  | 4 | 298 | WMD -206.09[-407.31,-4.8] | 97.2% | High | Very low |
|  |  |  |  |  | Hb loss(g/dl) |  | 4 | 298 | WMD 0.63[-0.96,0.31] | 68.2% | High | Low |
|  |  |  |  |  | blood transfusions rates | total | 6 | 647 | RR 0.28[0.19,0.42] | 14.0% | Low | High |
|  |  |  |  |  |  | CAS-TKR | 2 | 128 | RR 0.33[0.14,0.79] | N/A | High | Moderate |
|  |  |  |  |  |  | Anticoagulant | 4 | 329 | RR 0.43[0.23,0.80] | N/A | High | Moderate |
|  |  |  |  |  |  | Total dose<1.5 g | 2 | 168 | RR 0.20[0.12,0.35] | N/A | High | Moderate |
|  |  |  |  |  |  | Total dose ≥1.5 g | 4 | 344 | RR 0.41[0.22,0.75] | N/A | High | Moderate |
|  |  |  |  |  |  | Clamped drain<2 h | 3 | 250 | RR 0.22[0.13,0.38] | N/A | High | Moderate |
|  |  |  |  |  |  | Clamped drain ≥2 h | 2 | 128 | RR 0.33[0.14,0.79] | N/A | High | Moderate |
|  |  |  |  |  |  | Transfusion protocol | 4 | 329 | RR 0.16[0.09,0.29] | N/A | High | High |
|  |  |  |  |  | deep vein thrombosis |  | 2 | 184 | RR 1.75[0.4,7.56] | N/A | High | Very low |
|  | | | | | | | | | | | | |
| IV 10-20mg/kg | total shoulder arthroplasty | Kirsch 2017 | RCT,non-RCT | High | change in hemoglobin(g/dL) |  | 5 | 632 | WMD -0.64[-0.84,-0.44] | 0.0% | High | Very low |
|  |  |  |  |  | drain output(ml) |  | 3 | 438 | WMD -116.80[-139.20,-94.40] | 76.0% | High | Very low |
|  |  |  |  |  | transfusion requirements |  | 5 | 632 | RR 0.45[0.18,1.09] | 0.0% | High | Very low |

A2. joint replacement surgery—THA

| Dosage | Surgery | Author year | Type of Incl. study | AMSTAR | Outcome | Subgroup | No. of  incl. study | No. of  Patient | Evidence synthesis | I2 | Public-ation bias | GRADE  quality |
| --- | --- | --- | --- | --- | --- | --- | --- | --- | --- | --- | --- | --- |
| IV 15mg/kg | total hip arthroplasty | Zhang H 2017 | RCT | High | total blood loss(ml) |  | 7 | 802 | WMD -146.29[-206.66,-85.92] | 83.0% | <0.00001* | Low |
|  |  |  |  |  | intraoperative blood loss(ml) |  | 5 | 654 | WMD -64.65[-74.75,-54.55] | 82.0% | <0.00001* | High |
|  |  |  |  |  | need for transfusion |  | 7 | 668 | RR 0.41[0.27,0.63] | 84.0% | <0.0001* | Low |
|  |  |  |  |  | the occurrence of deep venous thrombosis |  | 8 | 950 | RR 1.22[0.52,2.89] | 0.0% | 0.65 | Low |
|  | | | | | | | | | | | | |
| Inconsistent | THA&TKA | Ye 2020 | RCT | High | total blood loss(ml) |  | 7 | 692 | WMD -3.67[-45.12,37.78] | 0.0% | 0.86 | Low |
|  |  |  |  |  | decline in hemoglobin(g/dl) |  | 7 | 854 | WMD -0.03[-0.11,0.05] | 0.0% | 0.45 | Low |
|  |  |  |  |  | length of hospital stay(d) |  | 6 | 452 | WMD 0.09[-0.10,0.27] | 0.0% | 0.36 | Low |
|  |  |  |  |  | incidence of DVT or IVT |  | 4 | 626 | OR 0.37[0.10,1.40] | 0.0% | 0.14 | High |
|  |  |  |  |  | transfusion rate |  | 9 | 934 | OR 1.03[0.65,1.61] | 0.0% | 0.91 | Low |
|  | | | | | | | | | | | | |
| Inconsistent | THA&TKA | Sun 2019 | RCT | High | IV between topical- total blood loss(ml) | total | 14 |  | WMD 30.92[-28.40,90.25] | 87.0% | 0.31 | Low |
|  |  |  |  |  |  | TKA | 10 |  | WMD 52.69 [-18.58,123.97] | 89.9% | 0.15 | Low |
|  |  |  |  |  |  | THA | 4 |  | WMD -31.03[-156.16,94.10] | 70.3% | 0.63 | High |
|  |  |  |  |  | IV between topical-transfusion rate | total | 17 |  | RR 1.08[0.78,1.50] | 0.0% | 0.75 | Low |
|  |  |  |  |  |  | TKA | 12 |  | RR 1.25[0.80,1.96] | 8.3% | 0.32 | Low |
|  |  |  |  |  |  | THA | 5 |  | RR 0.80[0.46,1.37] | 0.0% | 0.41 | High |
|  |  |  |  |  | IV between topical-postoperative venous thromboembolism | total | 9 |  | RR 1.89[0.79,4.55] | 0.0% | 0.15 | Low |
|  |  |  |  |  |  | TKA | 5 |  | RR 2.14[0.74,6.18] | 0.0% | 0.16 | High |
|  |  |  |  |  |  | THA | 4 |  | RR 1.45[0.30,6.93] | 0.0% | 0.64 | High |
|  |  |  |  |  | combined between single route-total blood loss volume | total | 7 |  | WMD 198.07[88.46,307.67] | 92.3% | <0.05* | Low |
|  |  |  |  |  |  | TKA | 4 |  | WMD 168.34[85.44,251.25] | 59.4% | <0.05* | High |
|  |  |  |  |  |  | THA | 3 |  | WMD 210.36[13.34,407.39] | 96.3% | <0.05* | High |
|  |  |  |  |  | combined between single route-transfusion rate | total | 7 |  | RR 2.51[1.48,4.25] | 0.0% | <0.05* | Low |
|  |  |  |  |  |  | TKA | 4 |  | RR 2.09[0.72,6.07] | 0.0% | <0.05* | High |
|  |  |  |  |  |  | THA | 3 |  | RR 2.66[1.45,4.89] | 0.0% | <0.05* | High |
|  |  |  |  |  | combined between single route-postoperative venous thromboembolism | total | 6 |  | RR 0.80[0.27,2.35] | 0.0% | 0.68 | Low |
|  |  |  |  |  |  | TKA | 3 |  | RR 2.98[0.31,28.27] |  | 0.34 | High |
|  |  |  |  |  |  | THA | 3 |  | RR 0.54[ 0.16,1.84] |  | 0.32 | High |
|  | | | | | | | | | | | | |
| Inconsistent | THA&TKA | Chen 2016 | RCT | High | transfusion rates | TKA | 12 | 1026 | OR 0.90 [0.58,1.40] | 30.0% | 0.64 | Low |
|  |  |  |  |  |  | THA | 4 | 550 | OR 1.19[0.67,2.09] | 0.0% | 0.63 | High |
|  |  |  |  |  | total blood loss(ml) | TKA | 8 | 688 | WMD -28.72[-195.97,138.54] | 97.0% | 0.74 | Low |
|  |  |  |  |  |  | THA | 4 | 550 | WMD 38.66[-38.97,116.30] | 60.0% | 0.33 | High |
|  |  |  |  |  | total drain out(ml) | TKA | 12 | 1009 | WMD -3.09[-39.05,32.88] | 98.0% | 0.87 | Low |
|  |  |  |  |  |  | THA | 1 | 68 | WMD -31.00[-66.56,4.66] | / | 0.09 | High |
|  |  |  |  |  | hidden blood loss(ml) | TKA | 3 | 123 | WMD -43.35[-325.25,238.26] | 95.0% | 0.76 | High |
|  |  |  |  |  |  | THA | 2 | 208 | WMD -5.84[-38.44,26.75] | 0.0% | 0.73 | High |
|  |  |  |  |  | haemoglobin drop(g/dl) | TKA-day1 | 12 | 986 | WMD 0.18[-0.09,0.46] | 93.0% | 0.19 | Low |
|  |  |  |  |  |  | TKA-day2 | 3 | 208 | WMD -0.59[-1.40,0.23] | 98.0% | 0.16 | High |
|  |  |  |  |  |  | THA-day1 | 3 | 482 | WMD 0.30[-0.08,0.68] | 0.0% | 0.12 | High |
|  |  |  |  |  | length of hospital stay(d) | TKA | 2 | 178 | WMD -0.77[-1.65,0.10] | 73.0% | 0.08 | High |
|  |  |  |  |  |  | THA | 2 | 343 | WMD -0.05[-0.32,0.42] | 68.0% | 0.80 | High |
|  |  |  |  |  | complications | TKA-infection | 2 | 160 | OR 1.00[0.14,7.24] | 0.0% | 1.00 | High |
|  |  |  |  |  |  | TKA-DVT | 9 | 640 | OR 1.10[0.45,2.68] | 0.0% | 0.83 | Low |
|  |  |  |  |  |  | THA-infection | 1 | 140 | OR 1.52[0.25,9.49] | / | 0.65 | High |
|  |  |  |  |  |  | THA-DVT | 3 | 383 | OR 0.23[0.05,1.10] | 0.0% | 0.07 | High |
|  |  |  |  |  |  | THA-PE | 3 | 482 | OR 0.33[0.01,8.32] | / | 0.50 | High |
|  | | | | | | | | | | | | |
| IV 15mg/kg | total joint arthroplasty | Zhang XQ 2016 |  |  | total blood loss(ml) | total | 6 | 643 | WMD -138.70[-196.14,-81.26] | 65.0% | < 0.00001* | Low |
|  |  |  |  |  |  | TKA | 3 | 363 | WMD -189.75[-306.94,-72.56] | 70.0% | 0.002* | High |
|  |  |  |  |  |  | THA | 3 | 280 | WMD -95.62[-129.63,-61.60] | 0.0% | < 0.00001* | High |
|  |  |  |  |  | transfusion rates |  | 6 | 643 | RR 0.42[0.20,0.85] | 9.0% | 0.02* | Low |
|  |  |  |  |  | postoperative thromboembolic complications |  | 3 | 424 | RR 1.00[0.28,3.63] | 0.0% | 1 | High |
|  | | | | | | | | | | | | |
| Inconsistent | total hip arthroplasty | Huang 2015 | RCT | High | total blood loss(ml) | total | 15 | 866 | WMD -389.14[-483.05,-295.23] | 74.0% | Low | Moderate |
|  |  |  |  |  |  | TXA | 11 | 687 | WMD -369.17[-481.73,-256.61] | 73.0% | Low | Moderate |
|  |  |  |  |  |  | AP | 3 | 133 | WMD -471.70[-650.94,-292.47] | 72.0% | High | Low |
|  |  |  |  |  |  | EACA | 1 | 46 | WMD -331.00[-565.20,-96.80] | N/A | High | Low |
|  |  |  |  |  | mean nuit of transfusions per patient | total | 6 | 435 | SMD -0.65[-1.19,-0.12] | 93.0% | Low | Low |
|  |  |  |  |  |  | TXA | 1 | 50 | SMD 0.80[0.18,1.42] | N/A | High | Low |
|  |  |  |  |  |  | AP | 5 | 385 | SMD -0.92[-1.46,-0.38] | 93.0% | High | Very low |
|  |  |  |  |  | blood transfusion requirements | total | 21 | 1557 | RR 0.55[0.43,0.70] | 59.0% | Low | Moderate |
|  |  |  |  |  |  | TXA | 14 | 816 | RR 0.42[0.26,-.66] | 68.0% | Low | High |
|  |  |  |  |  |  | AP | 5 | 745 | RR 0.68[0.58,0.80] | 0.0% | High | Moderate |
|  |  |  |  |  |  | EACA | 2 | 76 | RR 0.85[0.37,1.96] | 0.0% | High | Low |
|  |  |  |  |  | deep-vein thrombosis |  | 25 | 1788 | RR 0.85[0.51,1.42] | 0.0% | Low | Moderate |
|  | | | | | | | | | | | | |
| IV 10-15mg/kg | total hip arthroplasty | Sukeik 2019 | RCT | High | wound complications |  | 25 | 1608 | RD -0.02[-0.04,-0.00] | 0.0% | 0.01* | Low |
|  |  |  |  |  | intraoperative blood loss(ml) |  | 13 | 834 | WMD -49[-61.-37] | 92.0% | 0.01* | Low |
|  |  |  |  |  | postoperative blood loss(ml) |  | 15 | 1048 | WMD -229[-245,-213] | 90.0% | <0.01* | Low |
|  |  |  |  |  | total blood loss(ml) |  | 13 | / | WMD -349[-390,-308] | 78.0% | <0.01* | Low |
|  |  |  |  |  | blood transfusion |  | 19 | 1331 | RD -0.19[-0.23,-0.15] | 72.0% | <0.01* | Low |
|  |  |  |  |  | deep venous thrombosis |  | 16 | 1432 | / | / | 0.95 | Low |
|  |  |  |  |  | pulmonary embolisms |  | 7 | / | / | / | 0.72 | Low |
|  |  |  |  |  | other complications |  | 25 | 1608 | / | / | 0.90 | Low |

N/R= not reported

TKA= total knee arthroplasty

THA= total hip arthroplasty

B. Other orthopedic surgery

| Dosage | Surgery | Author year | Type of Incl. study | AMSTAR | Outcome | Subgroup | No. of  incl. study | No. of  Patient | Evidence synthesis | I2 | Public-ation bias | GRADE  quality |
| --- | --- | --- | --- | --- | --- | --- | --- | --- | --- | --- | --- | --- |
| IV 10-15mg/kg | hip fracture surgery | Xiao 2019 | RCT | High | transfusion requirement |  | 11 | 892 | RR 0.60[0.38,0.93] | 94.0% | Low | Low |
|  |  |  |  |  | total blood loss(ml) |  | 6 | 416 | WMD -326.64[-462.23,-191.06] | 95.0% | Low | Low |
|  |  |  |  |  | deep vein thrombosis |  | 10 | 854 | RD 0.02[-0.01,0.04] | 0.0% | Low | Moderate |
|  |  |  |  |  | total thromboembolic events |  | 10 | 854 | RD 0.02[-0.01,0.05] | 32.0% | Low | Moderate |
|  | | | | | | | | | | | | |
| Inconsistent | spine surgery | Chen J 2021 | RCT | High | intraoperative blood loss(ml) |  | 7 | 587 | WMD -210.38[-267.31,-153.45] | 62.0% | Low | Moderate |
|  |  |  |  |  | postoperative drainage(ml) |  | 4 | 336 | WMD -113.40[-126.97,-99.83] | 11.0% | High | Moderate |
|  |  |  |  |  | total perioperative blood loss(ml) | total | 10 | 802 | WMD -266.85[-351.18,-182.52] | 62.0% | Low | Moderate |
|  |  |  |  |  |  | continuous TXA | 8 | 674 | WMD -214.68[-281.10,-148.27] | 37.0% | Low | High |
|  |  |  |  |  |  | non-continuous TXA | 2 | 128 | WMD -402.52[-522.74,-282.29] | 0.0% | High | Moderate |
|  |  |  |  |  | postoperative hemoglobin |  | 6 | 492 | SMD 0.20[0.02,0.38] | 42.0% | Low | High |
|  |  |  |  |  | operation time(min) |  | 6 | 509 | WMD -7.75[-16.65,1.15] | 0.0% | Low | Moderate |
|  |  |  |  |  | length of hospital stay(d) |  | 5 | 426 | WMD -1.09[-1.86,-0.32] | 56.0% | High | Low |
|  |  |  |  |  | blood transfusion rate |  | 5 | 471 | RR 0.61[0.48,0.79] | 45.0% | High | Moderate |
|  |  |  |  |  | thrombotic complications |  | 4 | 351 | RR 0.92[0.47,1.82] | 0.0% | High | Low |
|  | | | | | | | | | | | | |
| IV 10-30mg/kg | spinal fusion surgery | Du 2018 | RCT | High | intraoperative blood loss | total | 6 | 394 | SMD -0.32[-0.58,-0.06] | 40.0% | Low | High |
|  |  |  |  |  |  | intravenous | 4 | 254 | SMD -0.52[-0.77,-0.27] | 0.0% | High | Moderate |
|  |  |  |  |  |  | topical | 2 | 140 | SMD 0.04[-0.32,0.41] | 17.0% | High | Low |
|  |  |  |  |  | drain |  | 4 | 286 | SMD -1.12[-1.59,-0.64] | 71.0% | High | Low |
|  |  |  |  |  | hemoglobin |  | 4 | 254 | SMD -0.10[-0.56,0.37] | 70.0% | High | Very low |
|  |  |  |  |  | transfusion |  | 3 | 231 | RR 0.44[0.16,1.19] | 44.0% | High | High |
|  |  |  |  |  | hematocrit 1 day after surgery |  | 4 | 254 | SMD -0.34[-1.08,0.40] | 88.0% | High | Very low |
|  |  |  |  |  | Duration of hospitalization |  | 2 | 140 | SMD -1.00[-1.68,-0.32] | 73.0% | High | Low |
|  | | | | | | | | | | | | |
| Inconsistent | intertrochanteric fracture surgery | Luo 2019 | RCT | High | mean total blood loss(ml) |  | 4 | 339 | WMD -172.83[-241.43,-104.23] | 0.0% | High | Moderate |
|  |  |  |  |  | intra-ostoperative visible blood loss(ml) |  | 4 | 339 | WMD -33.46[-52.40,-14.52] | 0.0% | High | Moderate |
|  |  |  |  |  | intra- and postoperative transfusion rate |  | 5 | 540 | RR 0.71[0.52,0.97] | 63.0% | High | Low |
|  |  |  |  |  | hidden blood loss(ml) |  | 3 | 267 | WMD -144.20[-210.74,-77.66] | 0.0% | High | Moderate |
|  |  |  |  |  | overall incidence of thrombotic event | topical or intravenous | 5 | 539 | RR 0.84[0.46,1.54] | 0.0% | High | Low |
|  |  |  |  |  |  | intravenous | 4 | 239 | RR 0.76[0.30,1.90] | 0.0% | High | Low |
|  |  |  |  |  | hemoglobin values on postoperative day 3 |  | 3 | 364 | WMD 0.32[-0.09,0.74] | 27.0% | High | Low |
|  |  |  |  |  | red blood cell infusion9 |  | 5 | 540 | WMD -0.43[-0.63,-0.23] | 54.0% | High | Low |
|  |  |  |  |  | mortality on postoperative day 30 |  | 3 | 239 | RR 1.69[0.20,14.20] | 44.0% | High | Low |
|  | | | | | | | | | | | | |
| Inconsistent | intertrochanteric fracture surgery | Zhou 2019 | RCT | High | postoperative blood loss(ml) |  | 3 | 314 | WMD -14.38[-20.83,-7.93] | 30.0% | High | Moderate |
|  |  |  |  |  | hidden blood loss(ml) |  | 2 | 177 | WMD -139.05[-213.67,-64.43] | 0.0% | High | Moderate |
|  |  |  |  |  | total blood loss(ml) |  | 4 | 309 | WMD -177.83[-332.49,-23.18] | 86.0% | High | Very low |
|  |  |  |  |  | intraoperative blood loss(ml) |  | 4 | 309 | WMD -31.89[-73.32, 9.53] | 77.0% | High | Very low |
|  |  |  |  |  | transfusion |  | 8 | 836 | OR 0.50[0.36,0.69] | 0.0% | Low | High |
|  |  |  |  |  | deep venous thrombosis |  | 5 | 539 | OR 1.34[0.49,3.69] | 0.0% | High | Low |
|  | | | | | | | | | | | | |
| Inconsistent | periacetabular osteotomy and high tibial osteotomy | Yao 2019 | RCT | High | total blood loss in PAO(ml) |  | 3 | 333 | WMD -330.49[-390.16,-270.83] | 30.0% | High | Moderate |
|  |  |  |  |  | total blood loss in HTO(ml) |  | 3 | 332 | WMD -252.50[-356.81,-148.18] | 80.0% | High | Very low |
|  |  |  |  |  | hemoglobin decline | total | 5 | 565 | WMD -0.74[-1.09,-0.38] | 71.0% | High | Low |
|  |  |  |  |  |  | PAO | 2 | 233 | WMD -0.56[-0.94,-0.17] | 10.0% | High | Moderate |
|  |  |  |  |  |  | HTO | 3 | 232 | WMD -0.82[-1.34,-0.30] | 81.0% | High | Very low |
|  |  |  |  |  | transfusion rates | total | 6 | 665 | RR 0.26[0.09,0.75] | 70.0% | Low | High |
|  |  |  |  |  |  | PAO | 3 | 333 | RR 0.25[0.08,0.86] | 79.0% | High | Low |
|  |  |  |  |  |  | HTO | 3 | 332 | RR 0.20[0.01,4.10] | 10.0% | High | Moderate |
|  |  |  |  |  | wound complications |  | 5 | 565 | RR 0.62[0.13,2.04] | 0.0% | High | Low |
|  | | | | | | | | | | | | |
| Inconsistent | major orthopedic surgery | Huang 2013 | RCT | High | intraoperative total blood loss(ml) | total | 21 | 1259 | WMD -125.65[-182.58,-68.72] | 83.0% | Low | Low |
|  |  |  |  |  |  | knee | 7 | 389 | WMD -54.62[-129.52,20.29] | 82.0% | Low | Low |
|  |  |  |  |  |  | hip | 8 | 463 | WMD -157.74[-268.46,-47.03] | 79.0% | Low | Low |
|  |  |  |  |  |  | spine | 6 | 407 | WMD -306.87[-182.58,-68.72] | 88.0% | Low | Low |
|  |  |  |  |  | total blood loss(ml) | total | 24 | 1696 | WMD -408.33[-505.69,-310.97] | 89.0% | Low | Low |
|  |  |  |  |  |  | knee | 14 | 873 | WMD -459.82[-582.08,-337.56] | 85.0% | Low | Low |
|  |  |  |  |  |  | hip | 9 | 488 | WMD -331.00[-436.35,-225.65] | 54.0% | Low | Moderate |
|  |  |  |  |  |  | spine | 5 | 335 | WMD -438.57[-734.51,-142.62] | 83.0% | High | Very low |
|  |  |  |  |  | postoperative total blood loss(ml) | total | 19 | 1267 | WMD -214.58[-274.63,-154.52] | 90.0% | Low | Low |
|  |  |  |  |  |  | knee | 7 | 600 | WMD -306.60[-456.54,-156.67] | 93.0% | Low | Low |
|  |  |  |  |  |  | hip | 8 | 416 | WMD -157.85[-232.36,-83.34] | 73.0% | Low | Moderate |
|  |  |  |  |  |  | spine | 3 | 251 | WMD -95.12[-142.65,-47.60] | 21.0% | High | Moderate |
|  |  |  |  |  | blood units transfused per patient(U) |  | 11 | 917 | WMD -0.78[-0.19,-0.37] | 95.0% | Low | Low |
|  |  |  |  |  | blood volumes of blood transfused per patient(ml) |  | 7 | 397 | WMD -205.33[-301.37,-109.28] | 47.0% | Low | High |
|  |  |  |  |  | transfusion and deep vein thrombosis |  | 44 | 2689 | RR 1.11[0.69,1.79] | 0.0% | Low | Moderate |
|  |  |  |  |  | transfusion requirements |  | 42 | 2649 | RR 0.51[0.46,0.56] | 49.0% | Low | High |

C. Cerebral surgery.

| Dosage | Surgery | Author year | Type of Incl. study | AMSTAR | Outcome | Subgroup | No. of  incl. study | No. of  Patient | Evidence synthesis | I^2^ | Public-ation bias | GRADE  quality |
| --- | --- | --- | --- | --- | --- | --- | --- | --- | --- | --- | --- | --- |
| IV 1g | traumatic brain injury | July 2020 | RCT | High | mortality |  | 5 | 30262 | RR 0.92[0.87,0.97] | 0.0% | High | Moderate |
|  |  |  |  |  | hemorrhagic expansion |  | 4 | 938 | RR 0.79[0.64,0.97] | 0.0% | High | Moderate |
|  |  |  |  |  | neurosurgical intervention |  | 4 | 10566 | RR 0.99[0.89,1.12] | 5.0% | High | Low |
|  |  |  |  |  | unfavourable Glasgow Outcome Scale |  | 3 | 455 | RR 0.93[0.72,1.21] | 20.0% | High | Low |
|  |  |  |  |  | vascular occlusive events | total | 4 | 33625 | RR 0.85[0.71, 1.02] | 21.0% | High | Low |
|  |  |  |  |  |  | DVT | 4 | 33625 | RR 0.79[0.53,1.19] | 25.0% | High | Low |
|  |  |  |  |  |  | PE | 4 | 33625 | RR 0.91[0.70,1.20] | 0.0% | High | Low |
|  |  |  |  |  |  | stroke | 4 | 33625 | RR 0.83[0.54,1.27] | 41.0% | High | Low |
|  |  |  |  |  |  | MI | 3 | 33387 | RR 0.75[0.50,1.11] | 11.0% | High | Low |
|  | | | | | | | | | | | | |
| Inconsistent | cerebral Hemorrhage | Hu 2019 | RCT | High | mortality | total | 20 | 10253 | OR 0.89 [0.77, 1.02] | 26.0% | Low | Moderate |
|  |  |  |  |  |  | Day 7 | 1 | 2307 | OR 0.81[0.61,1.06] | N/A | High | Very low |
|  |  |  |  |  |  | 4 week | 1 | 270 | OR 0.55[0.27,1.12] | N/A | High | Very low |
|  |  |  |  |  |  | 6 week | 1 | 59 | OR 1.57[0.50,4.91] | N/A | High | Very low |
|  |  |  |  |  |  | Day 90 | 4 | 2869 | OR 0.99[0.84,1.18] | 0.0% | High | Low |
|  |  |  |  |  |  | 6 mouth | 2 | 605 | OR 1.01[0.51,2.01] | 53.0% | High | Very low |
|  |  |  |  |  |  | overall | 11 | 4143 | OR 0.82[0.62,1.08] | 43.0% | Low | Moderate |
|  |  |  |  |  |  | total | 11 | 4143 | OR 0.82[0.62,1.08] | 43.0% | Low | Moderate |
|  |  |  |  |  |  | administration within 8h | 2 | 2545 | OR 0.98[0.78,1.22] | 4.0% | High | Low |
|  |  |  |  |  |  | administration not limited to 8h | 9 | 1598 | OR 0.76[0.51,1.13] | 47.0% | Low | Moderate |
|  |  |  |  |  | poor functional outcomes | total | 6 | 3874 | OR 0.95[0.79,1.14] | 28.0% | Low | Moderate |
|  |  |  |  |  |  | administration within 8h | 2 | 2563 | OR 0.94[0.79,1.12] | 0.0% | High | Low |
|  |  |  |  |  |  | administration not limited to 8h | 4 | 1311 | OR 0.96[0.69,1.34] | 51.0% | High | Very low |
|  |  |  |  |  | hemorrhagic expansion | total | 11 | 4336 | OR 0.54[0.37,0.80] | 67.0% | Low | Moderate |
|  |  |  |  |  |  | SAH | 7 | 1719 | OR 0.48[0.30,0.78] | 59.0% | Low | High |
|  |  |  |  |  |  | Spons-ICH | 3 | 2379 | OR 0.75[0.30,1.85] | 21.0% | High | Low |
|  |  |  |  |  |  | Traumatic-ICH | 1 | 238 | OR 0.57[0.31,0.80] | N/A | High | Very low |
|  |  |  |  |  |  | administration within 30mins | 2 | 2355 | OR 0.44[0.06,3.37] | 56.0% | High | Low |
|  |  |  |  |  |  | administration not within 30mins | 1 | 24 | OR 1.62[0.14,18.58] | N/A | High | Very low |
|  |  |  |  |  | hemorrhage volume | total | 4 | 2687 | SMD -1.98[-3.00, -0.97] | 23.0% |  |  |
|  |  |  |  |  |  | less than 2ml | 2 | 110 | SMD -3.29[-4.95,-1.62] | 0.0% | High | Moderate |
|  |  |  |  |  |  | more than 2ml | 2 | 2577 | SMD -1.22[-2.49,0.06] | 23.0% | High | Moderate |
|  |  |  |  |  | adverse events | ACS or MI | 1 | 2325 | OR 1.85[0.68,5.01] | N/A | High | Very low |
|  |  |  |  |  |  | DVT | 5 | 2978 | OR 1.25[0.75,2.08] | 0.0% | High | Low |
|  |  |  |  |  |  | PE | 4 | 3366 | OR 0.97[0.59,1.58] | 0.0% | High | Low |
|  |  |  |  |  |  | ischaemic stroke or TIA | 5 | 3601 | OR 1.43[0.87,2.34] | 61.0% | High | Very low |
|  |  |  |  |  |  | combimed thrombotic events | 3 | 2904 | OR 1.47[1.07,2.01] | 23.0% | High | Moderate |
|  |  |  |  |  |  | hydrocephalus | 4 | 1091 | OR 1.21[0.90,1.62] | 0.0% | High | Low |
|  |  |  |  |  |  | seizure or convulsions | 1 | 2325 | OR 0.90[0.65,1.24] | N/A | High | Very low |
|  | | | | | | | | | | | | |
| IV 10-50mg/kg | craniosynostosis open surgery | Lu 2019 | 542 | RCT,comparative study | intraoperative RBC transfusion(mL/kg) |  | 9 | 542 | WMD -8.25[-12.27,-4.23] | 58.7% | Low | Very low |
|  |  |  |  |  |  | intraoperative RBC transfusion(ml) | 3 | 184 | WMD -94.19[-178.05,-10.33] | 88.1% | High | Very low |
|  |  |  |  |  | postoperative RBC transfusion |  | 5 | 408 | OR 0.12[0.03,0.53] | 63.4% | High | Very low |
|  |  |  |  |  | intraoperative blood loss(mL/kg) |  | 7 | 477 | SMD -10.96[-17.00,-4.92] | 73.9% | Low | Very low |
|  |  |  |  |  |  | intraoperative blood loss(ml) | 4 | 209 | WMD -51.9[-84.40,-17.97] | 81.0% | Low | Very low |
|  |  |  |  |  | FFP transfusion |  | 3 | unclear | OR 0.24[0.01,4.07] | 75.0% | Low | Very low |
|  |  |  |  |  | crystalloid transfusion(mL/kg) |  | 3 | unclear | SMD 0.64[-11.42,12.81] | 57.0% | Low | Very low |
|  |  |  |  |  | operation time(min) |  | 7 | unclear | WMD -0.90[-6.61,4.81] | 28.0% | Low | Very low |
|  |  |  |  |  | length of stay-ICU(d) |  | 3 | unclear | WMD -0.17[-0.53,0.19] | 0.0% | Low | Very low |
|  |  |  |  |  | length of stay-hospital(d) |  | 6 | unclear | WMD -0.34[-0.76,0.07] | 67.0% | Low | Very low |
|  |  |  |  |  | complications |  | 6 | unclear | OR 0.53[0.07,4.19] | 0.0% | Low | Very low |
|  |  |  |  |  | Urinary output(mL/kg) |  | 3 | unclear | WMD 0.31[-0.85,1.47] | 0.0% | Low | Very low |
|  |  |  |  |  | hermatocrit |  | 4 | unclear | SMD 0.70[-2.31,3.72] | 81.0% | Low | Very low |

D. Cardiac surgery

| Dosage | Surgery | Author year | Type of Incl. study | AMSTAR | Outcome | Subgroup | No. of  incl. study | No. of  Patient | Evidence synthesis | I^2^ | Public-ation bias | GRADE  quality |
| --- | --- | --- | --- | --- | --- | --- | --- | --- | --- | --- | --- | --- |
| IV 10-15mg/kg | CABG | Zhang 2019 | RCT | High | cerebrovascular acident | total | 22 | 6775 | RR 0.83[0.62,1.39] | 0.0% | Low | Moderate |
|  |  |  |  |  |  | on-pump | 13 | 1397 | RR 0.95[0.44,2.06] | 0.0% | Low | Moderate |
|  |  |  |  |  |  | Mixed | 1 | 4629 | RR 0.92[0.57,1.48] | N/A | High | Very low |
|  |  |  |  |  |  | off-pump | 9 | 749 | Not estimable | N/A | Low | Low |
|  |  |  |  |  | seizures |  | 4 | 4911 | RR 6.67[1.77,25.20] | 0.0% | High | High |
|  |  |  |  |  | operation for bleeding | total | 16 | 6259 | RR 0.46[0.31,0.68] | 0.0% | Low | High |
|  |  |  |  |  |  | on-pump | 11 | 1194 | RR 0.60[0.34,1.07] | 0.0% | Low | Moderate |
|  |  |  |  |  |  | Mixed | 1 | 4630 | RR 0.38[0.22,0.65] | N/A | High | Low |
|  |  |  |  |  |  | off-pump | 5 | 435 | RR 0.33[0.01,7.90] | N/A | High | Low |
|  |  |  |  |  | postoperative mortality | total | 17 | 6259 | RR 0.82[0.53,1.28] | 0.0% | Low | Moderate |
|  |  |  |  |  |  | on-pump | 12 | 1302 | RR 0.93[0.36,2.38] | 0.0% | Low | Moderate |
|  |  |  |  |  |  | Mixed | 1 | 4641 | RR 0.79[0.47,1.31] | N/A | High | Very low |
|  |  |  |  |  |  | off-pump | 5 | 471 | RR 0.96[0.06,14.55] | N/A | High | Very low |
|  |  |  |  |  | myocardial infarction | total | 23 | 6714 | RR 0.90[0.78,1.05] | 0.0% | Low | Moderate |
|  |  |  |  |  |  | on-pump | 13 | 1286 | RR 0.90[0.51,1.58] | 0.0% | Low | Moderate |
|  |  |  |  |  |  | Mixed | 1 | 4630 | RR 0.90[0.77,1.05] | 0.0% | High | Very low |
|  |  |  |  |  |  | off-pump | 9 | 798 | RR 1.56[0.22,11.23] | 0.0% | Low | Moderate |
|  |  |  |  |  | acute renal insufficiency | total | 14 | 5954 | RR 1.01[0.78,1.30] | 0.0% | Low | Moderate |
|  |  |  |  |  |  | on-pump | 8 | 836 | RR 0.91[0.36,2.29] | 0.0% | Low | Moderate |
|  |  |  |  |  |  | Mixed | 1 | 4629 | RR 1.03[0.78,1.35] | 0.0% | High | Very low |
|  |  |  |  |  |  | off-pump | 5 | 489 | RR 0.85[0.29,2.47] | 0.0% | High | Low |
|  |  |  |  |  | allogeneic transfusions rate | total | 11 | 5360 | RR 0.60[0.52,0.78] | 76.0% | Low | Low |
|  |  |  |  |  |  | on-pump | 7 | 523 | RR 0.68[0.47,1.00] | 81.0% | Low | Low |
|  |  |  |  |  |  | Mixed | 1 | 4631 | RR 0.69[0.65,0.74] | 76.0% | High | Very low |
|  |  |  |  |  |  | off-pump | 3 | 206 | RR 0.32[0.19,0.54] | 0.0% | High | High |
|  |  |  |  |  | 24h postoperative chest tube drainage(ml) | total | 16 | 6247 | WMD -206.19[-248.13,-164.15] | 72.0% | Low | Moderate |
|  |  |  |  |  |  | on-pump | 9 | 984 | WMD -211.36[-261.13,-159.59] | 20.0% | Low | High |
|  |  |  |  |  |  | Mixed | 1 | 4631 | WMD -134.00[-148.23,-164.15] | 72.0% | High | Very low |
|  |  |  |  |  |  | off-pump | 7 | 632 | WMD -220.25[-290.58,-149.91] | 76.0% | Low | Low |
|  | | | | | | | | | | | | |
| Inconsistent | cardiac surgery | Khaie 2019 | RCT | High | adverse events | vs placebo | 44 | 9896 | RR 0.97[0.88,1.07] | 0.0% | Low | Moderate |
|  |  |  |  |  |  | vs EACA | 4 | NR | RR 1.03 [0.97,1.10] | N/A | High | Very low |
|  |  |  |  |  |  | vs aprotinin | 21 | NR | RR 0.93[0.88,0.98] | N/A | Low | Low |
|  | | | | | | | | | | | | |
| Inconsistent | cardiac surgery | Guo 2019 | RCT | High | transfusion rate | total | 31 | 8925 | RR 0.71[0.65,0.78] | 49.0% | Low | High |
|  |  |  |  |  |  | total | 28 | 8138 | RR 0.69[0.66,0.73] | 22.0% | Low | High |
|  |  |  |  |  |  | Continuous  +bolus | 20 | 2310 | RR 0.70[0.65,0.76] | 10.0% | Low | High |
|  |  |  |  |  |  | bolus | 10 | 5828 | RR 0.69[0.64,0.74] | 40.0% | Low | High |
|  |  |  |  |  |  | total | 27 | 8016 | RR 0.69[0.65,0.72] | 41.0% | Low | High |
|  |  |  |  |  |  | Continuous  +bolus low dose | 7 | 265 | RR 0.50[0.38,0.67] | 44.0% | Low | High |
|  |  |  |  |  |  | Continuous  +bolus high dose | 12 | 2043 | RR 0.71[0.65,0.77] | 21.0% | Low | High |
|  |  |  |  |  |  | bolus low dose | 5 | 496 | RR 0.69[0.65,0.74] | 0.0% | High | Moderate |
|  |  |  |  |  |  | bolus high dose | 4 | 5212 | RR 0.69[0.65,0.72] | 71.0% | High | Very low |
|  |  |  |  |  |  | total | 30 | 3959 | RR 0.69[0.62,0.77] | 51.0% | Low | Moderate |
|  |  |  |  |  |  | on-pump | 23 | 3299 | RR 0.71[0.63,0.80] | 53.0% | Low | Moderate |
|  |  |  |  |  |  | off-pump | 7 | 660 | RR 0.60[0.44,0.83] | 42.0% | Low | High |
|  |  |  |  |  |  | total | 30 | 8409 | RR 0.71[0.68,0.75] | 47.0% | Low | High |
|  |  |  |  |  |  | CABG | 22 | 7224 | RR 0.69[0.65,0.73] | 40.0% | Low | High |
|  |  |  |  |  |  | all kinds combined | 8 | 1185 | RR 0.85[0.74,0.96] | 40.0% | Low | High |
|  |  |  |  |  |  | total | 32 | 8820 | RR 0.72[0.69,0.76] | 46.0% | Low | High |
|  |  |  |  |  |  | intravenous | 28 | 8053 | RR 0.70[0.66,0.74] | 11.0% | Low | High |
|  |  |  |  |  |  | topical | 4 | 797 | RR 1.02[0.87,1.20] | 0.0% | High | Moderate |
|  |  |  |  |  | transfusion volume | all patients | 10 | 2105 | SMD -0.60[-0.85,-0.35] | 92.0% | Low | Low |
|  |  |  |  |  |  | transfused patients | 14 | 6610 | SMD -1.02[-1.47,-0.56] | 94.0% | Low | Low |
|  |  |  |  |  | post-operative blood loss(ml) |  | 44 | 5560 | WMD -246.98[-287.89,-206.06] | 97.0% | Low | Low |
|  |  |  |  |  | re-operation rate |  | 32 | 8937 | SMD 0.62[0.49,0.79] | 0.0% | Low | High |
|  |  |  |  |  | seizure | high dose | 5 | 5807 | RR 4.83[1.75,13.33] | 0.0% | High | High |
|  |  |  |  |  |  | low dose | 3 | 313 | Not estimable |  |  |  |
|  |  |  |  |  | mortality |  | 29 | 8907 | RR 0.78[0.54,1.14] | 0.0% | Low | Moderate |
|  |  |  |  |  | stroke |  | 32 | 9257 | RR 0.88[0.61,1.28] | 0.0% | Low | Moderate |
|  |  |  |  |  | myocardial infraction |  | 32 | 8688 | RR 0.89[0.77,1.04] | 0.0% | Low | Moderate |
|  |  |  |  |  | pulmonary embolism |  | 18 | 6587 | RR 1.08[0.59,2.00] | 0.0% | Low | Moderate |
|  |  |  |  |  | renal dysfunction |  | 19 | 7210 | RR 0.99[0.77,1.27] | 0.0% | Low | Moderate |

CABG=coronary artery bypass grafting surgery

E. Nasal surgery

| Dosage | Surgery | Author year | Type of Incl. study | AMSTAR | Outcome | Subgroup | No. of  incl. study | No. of  Patient | Evidence synthesis | I2 | Public-ation bias | GRADE  quality |
| --- | --- | --- | --- | --- | --- | --- | --- | --- | --- | --- | --- | --- |
| IV 10-15 mg/kg | endoscopic sinus surgery | Kim 2019 | RCT | High | intraoperative blood loss |  | 7 | 562 | SMD -0.66 [-0.86,-0.46] | 26.1% | Low | High |
|  |  |  |  |  | surgical field score |  | 5 | 332 | SMD -0.71 [-1.03,-0.38] | 31.0% | High | Moderate |
|  |  |  |  |  | operative time |  | 4 | 232 | SMD -0.60 [-0.93, -0.27] | 33.1% | High | Moderate |
|  |  |  |  |  | intraoperative blood pressure |  | 2 | 198 | SMD 0.08 [-0.20, 0.37] | 0.0% | High | Low |
|  |  |  |  |  | postoperative nausea and vomiting |  | 5 | 434 | OR 0.89 [0.44, 1.80] | 0.0% | High | Low |
|  |  |  |  |  | postoperative thrombotic accident |  | 4 | 374 | OR 0.91 [0.13, 6.57] | 0.0% | High | Low |
|  |  |  |  |  | Coagulation function | platelet count | 3 | 254 | SMD -0.18 [-0.42, 0.07] | 0.0% | High | Low |
|  |  |  |  |  |  | prothrombin time | 3 | 254 | SMD 0.16 [-0.19, 0.50] | 48.4% | High | Low |
|  |  |  |  |  |  | partial thromboplastin time | 3 | 254 | SMD -0.17 [-0.62, 0.27] | 66.9% | High | Very low |
|  | | | | | | | | | | | | |
| IV 1-2g | sinus surgery | Kang 2019 | RCT | Moderate | intraoperative blood loss |  | 4 | 226 | SMD -0.71 [-1.03, -0.38] | 31.0% | High | Moderate |
|  |  |  |  |  | surgical field score |  | 4 | 226 | SMD -0.89 [-1.32, -0.45] | 59.4% | High | Low |
|  |  |  |  |  | operative time |  | 2 | 110 | SMD -0.25 [-0.71, 0.21] | 32.1% | High | Low |
|  |  |  |  |  | intraoperative blood pressure |  | NR | NR | SMD -0.29 [-1.51, 0.94] | 93.2% | High | Very low |
|  |  |  |  |  | postoperative nausea and vomiting |  | 2 | 110 | OR 2.41 [0.32, 18.00] | 0.0% | High | Low |
|  |  |  |  |  | postoperative thrombotic accident |  | 2 | 110 | OR 1.00 [-16.44, 16.44] | 0.0% | High | Low |
|  |  |  |  |  | Coagulation function | prothrombin time | 2 | 110 | SMD -0.01 [-0.38, 0.36] | 0.0% | High | Low |
|  |  |  |  |  |  | partial thromboplastin time | 2 | 110 | SMD -0.32 [-0.69, 0.06] | 0.0% | High | Low |
|  | | | | | | | | | | | | |
| Inconsistent | rhinoplasty | Juliana 2018 | RCT | High | intraoperative blood loss (ml) | Oral or Intravenous | 4 | 246 | WMD -42.28 [-70.36, -14.21] | 84.0% | High | Very low |
|  |  |  |  |  |  | Oral | 2 | 100 | WMD -61.70 [-83.02, -40.39] | 0.0% | High | Low |
|  |  |  |  |  |  | Intravenous | 2 | 146 | WMD -23.88 [-45.19, -2.58] | 56.0% | High | Very low |
|  |  |  |  |  | Eyelid edema (score) | Upper POD1 | NR | NR | WMD -1.06 [-1.59, -0.54] | 0.0% | High | Moderate |
|  |  |  |  |  |  | POD3 | NR | NR | WMD -0.99 [-1.63, -0.36] | 0.0% | High | Moderate |
|  |  |  |  |  |  | POD7 | NR | NR | WMD -0.80 [-1.15, -0.45] | 0.0% | High | Moderate |
|  |  |  |  |  |  | Lower POD1 | NR | NR | WMD -0.86 [-1.15, -0.57] | 0.0% | High | Moderate |
|  |  |  |  |  |  | POD3 | NR | NR | WMD -0.69 [-1.03, -0.34] | 0.0% | High | Moderate |
|  |  |  |  |  |  | POD7 | NR | NR | WMD -0.76 [-1.04, -0.49] | 0.0% | High | Moderate |
|  |  |  |  |  | Postrhinoplasty ecchymosis (score) | Upper POD1 | NR | NR | WMD -1.24 [-2.34, -0.14] | 0.0% | High | Moderate |
|  |  |  |  |  |  | POD3 | NR | NR | WMD -1.53 [-2.29, -0.78] | 0.0% | High | Moderate |
|  |  |  |  |  |  | POD7 | NR | NR | WMD -1.33 [-2.62, -0.04] | 0.0% | High | Moderate |
|  |  |  |  |  |  | POD1 | NR | NR | WMD -0.72 [-1.32, -0.11] | 0.0% | High | Moderate |
|  |  |  |  |  |  | POD3 | NR | NR | WMD -0.95 [-1.27, -0.63] | 0.0% | High | Moderate |
|  |  |  |  |  |  | POD7 | NR | NR | WMD -0.94 [-1.80, -0.08] | 0.0% | High | Moderate |
|  | | | | | | | | | | | | |
| Topical 400mg | epistaxis | Joseph 2018 | RCT | High | re-bleeding proportion | Oral or Topical | 3 | 225 | RR 0.71 [0.56, 0.90] | 0.0% | High | Moderate |
|  |  |  |  |  |  | Oral | 2 | 157 | RR 0.73 [0.55, 0.96] | 0.0% | High | Moderate |
|  |  |  |  |  |  | Topical | 1 | 68 | RR 0.66 [0.41, 1.05] | N/A | High | Very low |
|  |  |  |  |  | stop initial bleeding within 30 mins | Topical | 1 | 68 | RR 0.79 [0.56, 1.11] | N/A | High | Very low |
|  |  |  |  |  | transfusion requirement | Oral | 1 | 89 | RR 0.81 [0.27, 2.48] | N/A | High | Very low |
|  |  |  |  |  | compare to other haemostatic agents | Topical | 3 | 460 | RR 2.35 [1.90, 2.92] | 0.0% | High | Moderate |

NR=not reported

F. Obstetrics and gynecological surgery

| Dosage | Surgery | Author year | Type of Incl. study | AMSTAR | Outcome | Subgroup | No. of  incl. study | No. of  Patient | Evidence synthesis | I2 | Public-ation bias | GRADE  quality |
| --- | --- | --- | --- | --- | --- | --- | --- | --- | --- | --- | --- | --- |
| IV 10  mg/kg | caesarean section | Wang 2019 | RCT | High | intraoperative blood loss (ml) | Total | 14 | 1665 | WMD -155.23 [-195.64, -114.81] | 96.0% | High | Very low |
|  |  |  |  |  |  | Elective surgery | 5 | 634 | WMD -184.96 [-200.43, -169.50] | 96.0% | High | Very low |
|  |  |  |  |  | postoperative blood loss (ml) | Total | 12 | 1398 | WMD -26.67 [-32.98, -20.36] | 99.0% | Low | Low |
|  |  |  |  |  |  | Elective surgery | 4 | 514 | WMD -47.66 [-67.66, -27.67] | 98.0% | High | Very low |
|  |  |  |  |  | total blood loss (ml) | Total | 11 | 2777 | WMD -184.88 [-218.83, -150.94] | 97.0% | High | Very low |
|  |  |  |  |  |  | Elective surgery | 6 | 1994 | WMD -191.34 [-237.72, -144.96] | 96.0% | High | Very low |
|  |  |  |  |  | transfusion requirement | Total | 9 | 1472 | RR 0.29 [0.18, 0.49] | 0.0% | Low | High |
|  |  |  |  |  |  | Elective surgery | 5 | 1218 | RR 0.33 [0.17, 0.63] | 0.0% | High | High |
|  |  |  |  |  | hemoglobin drop (g/dL) | Total | 9 | 1807 | WMD -0.80 [-1.07, -0.53] | 97.0% | High | Very low |
|  |  |  |  |  |  | Elective surgery | 6 | 1374 | WMD -0.73 [-0.86, -0.61] | 75.0% | Low | Low |
|  |  |  |  |  | hematocrit drop | Total | 3 | 1053 | WMD -2.05 [-3.09, -1.01] | 86.0% | High | Very low |
|  |  |  |  |  |  | Elective surgery | 1 | 740 | WMD -2.48 [-2.96, -2.00] | N/A | High | Low |
|  |  |  |  |  | additional uterotonic agents use | Total | 7 | 1900 | RR 0.40 [0.30, 0.55] | 7.0% | Low | High |
|  |  |  |  |  |  | Elective surgery | 4 | 1720 | RR 0.46 [0.32, 0.66] | 28.0% | High | High |
|  |  |  |  |  | massive hemorrhage | Total | 8 | 2234 | RR 0.39 [0.30, 0.51] | 64.0% | Low | High |
|  |  |  |  |  |  | Elective surgery | 6 | 2018 | RR 0.33 [0.22, 0.48] | 75.0% | Low | Moderate |
|  | | | | | | | | | | | | |
| IV 1g | vaginal delivery | Xia 2020 | RCT | High | total blood loss |  | 4 | 4579 | WMD -65.61 [-115.01, -16.21] | 85.0% | High | Very low |
|  |  |  |  |  | intraoperative blood loss |  | 3 | 4140 | WMD -14.30 [-28.39, -0.22] | 0.0% | High | Moderate |
|  |  |  |  |  | postoperative blood loss |  | 2 | 301 | WMD -41.24 [-55.50, -26.98] | 0.0% | High | Moderate |
|  |  |  |  |  | postpartum hemorrhage |  | 4 | 4579 | RR 0.48 [0.25, 0.91] | 73.0% | High | Moderate |
|  |  |  |  |  | severe postpartum hemorrhage |  | 3 | 4398 | RR 0.78 [0.54, 1.13] | 0.0% | High | Low |
|  |  |  |  |  | transfusion requirement |  | 2 | 4278 | RR 0.87 [0.46, 1.64] | 0.0% | High | Low |
|  |  |  |  |  | postoperative adverse event | nausea or vomiting | 2 | 4020 | RR 2.17 [1.62, 2.90] | 0.0% | High | High |
|  |  |  |  |  |  | nausea | 2 | 4450 | RR 2.24 [1.67, 3.01] | 0.0% | High | High |
|  |  |  |  |  |  | vomiting | 2 | 4430 | RR 2.19 [1.56, 3.07] | 0.0% | High | High |
|  |  |  |  |  |  | dizziness | 2 | 4631 | RR 1.28 [0.83, 1.95] | 0.0% | High | Low |
|  |  |  |  |  |  | photopsia | 1 | 4072 | RR 1.00 [0.25, 3.99] | N/A | High | Low |

G. Other types of surgery

| Dosage | Surgery | Author year | Type of Incl. study | AMSTAR | Outcome | Subgroup | No. of  incl. study | No. of  Patient | Evidence synthesis | I2 | Public-ation bias | GRADE  quality |
| --- | --- | --- | --- | --- | --- | --- | --- | --- | --- | --- | --- | --- |
| Inconsistent | upper gastrointestinal bleeding | Twum-Barimah 2020 | RCT | High | mortality |  | 10 | 2013 | RR 0.59[0.43,0.82] | 0.0% | Low | High |
|  |  |  |  |  | re-bleeding |  | 8 | 1750 | RR 0.79[0.61,1.02] | 23.0% | Low | Moderate |
|  |  |  |  |  | surgical interventions |  | 9 | 1863 | RR 0.70[0.43,1.13] | 60.0% | Low | Low |
|  |  |  |  |  | blood transfusions rate |  | 8 | 1763 | RR 1.00[0.93,1.08] | 0.0% | Low | Moderate |
|  |  |  |  |  | tromboembolic events |  | 6 | 1041 | RR 0.89[0.17,4.59] | 55.0% | Low | Moderate |
|  |  |  |  |  | thrombophlebtis |  | 2 | 354 | RR 2.02[0.44,9.26] | 0.0% | High | Moderate |
|  | | | | | | | | | | | | |
| IV 500mg-1g | hemoptysis | Tsai 2020 | RCT | High | bleeding duration(h) |  | 2 | 70 | WMD -24.64[-35.98,-13.29] | 0.0% | High | Moderate |
|  |  |  |  |  | hemoptysis resolution |  | 3 | 117 | RR 1.26[0.86,1.86] | 77.0% | High | Low |
|  |  |  |  |  | bleeding volume(ml) |  | 2 | 90 | WMD -56.21[-17.72,-94.7] | N/A | High | Low |
|  |  |  |  |  | further intervention risk |  | 2 | 113 | OR 0.24 [0.08,0.67] | 0.0% | High | High |
|  |  |  |  |  | length of hospital stay(d) |  | 2 | 113 | WMD -1.62[-2.93,-0.31] | 0.0% | High | Moderate |
|  |  |  |  |  | adverse events |  | 2 | 70 | OR 3.15[0.85,11.63] | 26.0% | High | Moderate |
|  | | | | | | | | | | | | |
| IV 10-15mg/kg | prostate surgery | Longo 2018 | RCT | High | intraoperative blood loss | total | 6 | 738 | SMD -1.93[-2.81,-1.05] | 96.0% | Low | Low |
|  |  |  |  |  |  | prostatectomy | 3 | 462 | SMD -1.05[-1.80,-0.30] | 92.0% | High | Very low |
|  |  |  |  |  |  | turp | 3 | 276 | SMD -3.70[-6.17,-1.23] | 98.0% | High | Very low |
|  |  |  |  |  | transfusion rate | total | 7 | 718 | RR 0.61[0.47,0.80] | 0.0% |  |  |
|  |  |  |  |  |  | prostatectomy | 3 | 462 | RR 0.60[0.45,0.81] | 4.0% | Low | High |
|  |  |  |  |  |  | turp | 4 | 256 | RR 0.65[0.35,1.23] | 31.0% | High | Moderate |
|  |  |  |  |  | hemoglobin levels after 24h of surgery | total | 4 | 526 | SMD 0.22[-0.05,0.48] | 52.0% | High | Low |
|  |  |  |  |  |  | prostatectomy | 2 | 386 | SMD 0.14[-0.28,0.56] | 77.0% |  |  |
|  |  |  |  |  |  | turp | 2 | 140 | SMD 0.32[-0.04,0.68] | 13.0% | High | Very low |
|  |  |  |  |  | hemoglobin decline after 24h of surgery | total | 4 | 462 | SMD -0.06[-0.24,0.13] | 1.0% | High | Very low |
|  |  |  |  |  |  | prostatectomy | 1 | 186 | SMD -0.03[-0.31,0.26] | N/A | High | Low |
|  |  |  |  |  |  | turp | 3 | 276 | SMD -0.08[-0.32,0.16] | 1.0% |  |  |
|  | | | | | | | | | | | | |
| Topical 0.5g | minor oral surgery | Juliana 2016 | RCT | High | bleeding after minor oral surgeries | total | 5 | 262 | RR 0.13[0.05,0.36] | 0.0% | High | High |
|  |  |  |  |  |  | TXA vs placebo | 3 | 162 | RR 0.13[0.03,0.45] | 0.0% | High | High |
|  |  |  |  |  |  | TXA vs EACA | 1 | 51 | RR 0.12[0.01,0.94] | N/A | High | Moderate |
|  |  |  |  |  |  | TXA vs fibrin glue | 1 | 49 | RR 0.18 [0.01,3.52] | N/A | High | Low |
|  | | | | | | | | | | | | |
| IV 10mg/kg | tonsillectomy | Chan 2012 | RCT(3) CCS(4) | High | blood loss(ml) |  | 2 | 180 | WMD -32.72[-42.66,-22.78] | 0.0% | High | Moderate |
|  |  |  |  |  | post-operative haemorrhage |  | 5 | 1670 | RR 0.51[0.25,1.07] | 49.0% | High | Low |
|  |  |  |  |  | duration of haemorrhage(h) |  | 1 | 38 | WMD -3.60[-5.25,-1.95] | N/A | High | Very low |
|  |  |  |  |  | further interventions |  | 2 | 27 | RR 1.05[0.33,3.34] | N/A | High | Very low |
|  | | | | | | | | | | | | |
| Oral 3g-4g | heavy menstrual bleeding | Bryant-Smith 2018 | RCT | High | menstrual blood loss:mean loss-antifibrinolytics compared to no treatment or placebo(ml) |  | 4 | 565 | WMD -53.2[-62.7,-43.7] | 7.9% | High | Moderate |
|  |  |  |  |  | menstrual blood loss:improvement rates-antifibrinolytics compared to no treatment or placebo(ml) |  | 3 | 271 | RR 3.34[1.84,6.09] | 32.5% | High | High |
|  |  |  |  |  | quality of life scores-antifibrinolytics compared to no treatment or placebo | limitation in social/lesure activitues | 2 | 365 | SMD 0.52[0.31,0.74] | 0.0% | High | Moderate |
|  |  |  |  |  |  | limitation in physical activitues | 1 | 365 | SMD 0.55[0.34,0.77] | N/A | High | Low |
|  |  |  |  |  |  | limitation in work in or ouside the home | 1 | 187 | SMD 0.55[0.3,0.8] | N/A | High | Low |
|  |  |  |  |  | adverse events-antifibrinolytics compared to no treatment or placebo | any adverse event | 1 | 297 | RR 1.05[0.93,1.18] | N/A | High | Very low |
|  |  |  |  |  |  | gastrointestinal side effects | 2 | 244 | RR 1.07[0.55,2.07] | 0.0% | High | Low |
|  |  |  |  |  |  | headache | 2 | 244 | RR 1.09[0.82,1.45] | 0.0% | High | Low |
|  |  |  |  |  |  | uterine cancer | 1 | 55 | RR 1.38[0.06,32.36] | N/A | High | Very low |
|  |  |  |  |  |  | vaginal dryness | 1 | 55 | RR 0.15[0.01,3.6] | N/A | High | Moderate |
|  |  |  |  |  |  | dysmenorrhoea | 2 | 244 | RR 1.18[0.9,1.55] | 40.8% | High | Low |
|  |  |  |  |  |  | viral URTI | 2 | 486 | RR 1.19[0.58,2.47] | 27.6% | High | Low |
|  |  |  |  |  |  | fatigue | 2 | 486 | RR 1.65[0.68,3.97] | 0.0% | High | Low |
|  |  |  |  |  |  | musculoskeletal pain | 2 | 366 | RR 1.4[0.82,2.39] | 0.0% | High | Low |
|  |  |  |  |  |  | arthralgia | 2 | 486 | RR 1.78[0.72,4.41] | 0.0% | High | Low |
|  |  |  |  |  |  | myalgia | 2 | 486 | RR 2.52[0.78,8.16] | 0.0% | High | Moderate |
|  |  |  |  |  |  | nasal congestion | 1 | 297 | RR 6.77[0.4,113.42] | N/A | High | Moderate |
|  |  |  |  |  |  | sinusitis | 2 | 486 | RR 2.82[0.84,9.51] | 0.0% | High | Moderate |
|  |  |  |  |  |  | multiple allergies | 2 | 486 | RR 1.78[0.68,4.65] | 19.2% | High | Low |
|  |  |  |  |  |  | throat irritation | 1 | 297 | RR 1.02[0.22,4.79] | N/A | High | Very low |
|  |  |  |  |  |  | anaemia | 2 | 486 | RR 1.89[0.72,4.99] | 0.0% | High | Moderate |
|  |  |  |  |  |  | abdominal discomfort | 1 | 189 | RR 0.82[0.3,2.27] | N/A | High | Very low |
|  |  |  |  |  |  | cough | 1 | 189 | RR 0.86[0.28,2.61] | N/A | High | Very low |
|  |  |  |  |  |  | insomnia | 1 | 189 | RR 0.62[0.21,1.84] | N/A | High | Very low |
|  |  |  |  |  |  | dyspepsia | 1 | 189 | RR 0.23[0.06,0.84] | N/A | High | Moderate |
|  |  |  |  |  |  | migraine | 1 | 189 | RR 2.15[0.46,10.09] | N/A | High | Low |
|  | | | | | | | | | | | | |
| Inconsistent | any medical disciplines | Taeuber 2021 | RCT | High | Total Thromboembolic Events |  | 176 | 65900 | RD 0.001[-0.002, 0.003] | 0.0% | Low | High |
|  |  |  |  |  | Mortality |  | 63 | 55305 | RD -0.01[-0.015, -0.007] | N/A | Low | Moderate |
|  |  |  |  |  | Nonbleeding mortality |  | 48 | 46619 | RD -0.002[-0.006, 0.002] | N/A | Low | Low |
|  |  |  |  |  | Bleeding mortality |  | 49 | 46702 | RD -0.008[-0.011, -0.005] | N/A | Low | Moderate |
|  | | | | | | | | | | | | |
| 10-20  mg/kg | orthognathic surgery | Zhao 2019 | RCT | High | intraoperative blood loss(ml) | total | 10 | 655 | WMD -153.97[-166.52,-141.41] | 65.0% | Low | High |
|  |  |  |  |  |  | 10mg/kg-injection(mg/kg) | 3 | 290 | WMD -151.67[-164.40,-168.58] | 76.0% | High | Very low |
|  |  |  |  |  |  | 15mg/kg-injection(mg/kg) | 1 | 40 | WMD -385.00[-601.42,-168.58] | N/A | High | Very low |
|  |  |  |  |  |  | 20mg/kg-injection(mg/kg) | 3 | 133 | WMD -288.90[-400.86,-176.94] | 49.0% | High | Moderate |
|  |  |  |  |  |  | fixed-500mg-injection(mg) | 1 | 40 | WMD -85.01[-316.64,146.62] | N/A | High | Very low |
|  |  |  |  |  |  | fixed-1000mg-injection(mg) | 1 | 96 | WMD -129.21[-199.89,-58.53] | N/A | High | Very low |
|  |  |  |  |  |  | fixed-1000mg-irrigation(mg) | 1 | 56 | WMD -242.85[-386.72,-98.98] | N/A | High | Very low |
|  |  |  |  |  | haematocrit | total | 4 | 421 | SMD -2.32[-3.38, -1.26] | 66.0% | High | Low |
|  |  |  |  |  |  | hct-single maxillary | 1 | 200 | SMD -1.32[-1.87,-0.78] | 0.0% | High | Low |
|  |  |  |  |  |  | hct-bimaxillary | 3 | 221 | SMD -3,21[-.4.92,-1.51] | 56.0% | High | Low |
|  |  |  |  |  | operation time(min) | total | 8 | 655 | WMD -16.18[-19.60, -12.75] | 49.0% |  |  |
|  |  |  |  |  |  | Time-single maxiilary | 1 | 200 | WMD -17[-21.72,-14.10] | 78.0% | Low | High |
|  |  |  |  |  |  | Time-bimaxillary | 7 | 455 | WMD -8.88[-16.69,-1.07] | 27.0% | Low | Very low |
|  |  |  |  |  | quality of surgical field |  | 2 | 250 | SMD -1.01[-1.23,-.080] | 74.0% | High | Low |
|  |  |  |  |  | transfusion rates | total | 7 | 463 | OR 0.33[0.13, 0.83] | 0.0% | Low | High |
|  |  |  |  |  |  | transfusion-10mg/kg-injection | 3 | 290 | OR 0.22[0.04,1.32] | 35.0% | High | High |
|  |  |  |  |  |  | transfusion-15mg/kg-injection | 1 | 40 | OR 0.32[0.01,9.26] | N/A | High | Low |
|  |  |  |  |  |  | transfusion-20mg/kg-injection | 3 | 133 | OR 0.41[0.13,1.30] | 0.0% | High | High |
|  | | | | | | | | | | | | |
| Inconsistent | cancer | Montroy 2017 | RCT | High | venous thromboembolic events |  | 9 | 1075 | OR 0.60[0.28, 1.30] | 0.0% | Low | Moderate |
|  |  |  |  |  | blood transfusion |  | 7 | 955 | RR 0.52[0.34,0.80] | 67.0% | Low | Moderate |
|  |  |  |  |  | blood loss |  | 9 | 1109 | SMD -1.57[-2.21,-0.92] | 95.0% | Low | Low |
|  | | | | | | | | | | | | |
| Inconsistent | acute traumatic injury | Ker 2015 | RCT | High | antifibrinolytics versus control-mortality | total | 3 | 20437 | RR 0.90[0.85,0.96] | 13% | High | Moderate |
|  |  |  |  |  |  | TXA | 2 | 20367 | RR 0.90[0.85,0.97] | 0.0% | High | Moderate |
|  |  |  |  |  |  | aprotinin | 1 | 70 | RR 0.14[0.01,2.67] | N/A | High | Very low |
|  |  |  |  |  | myocardial infarction |  | 2 | 20437 | RR 0.61[0.40,0.92] | 0.0% | High | Moderate |
|  |  |  |  |  | stroke |  | 2 | 20437 | RR 0.86[0.61,1.23] | 0.0% | High | Low |
|  |  |  |  |  | deep vein thrombosis |  | 2 | 20437 | RR 0.95[0.62,1.47] | 0.0% | High | Low |
|  |  |  |  |  | pulmonary embolism |  | 2 | 20437 | RR 1.01[0.73,1.41] | 0.0% | High | Low |
|  |  |  |  |  | surgical intervention | total | 3 | 20437 | RR 1.00[0.97,1.03] | 5.0% | High | Low |
|  |  |  |  |  |  | TXA | 2 | 20437 | RR 1.00[0.97,1.03] | 40.0% | High | Low |
|  |  |  |  |  |  | aprotinin | 1 | 70 | RR 1.07[0.87,1.33] | 5.0% | High | Low |
|  |  |  |  |  | blood transfusion |  | 2 | 20367 | RR 0.98[0.96,1.01] | 0.0% | High | Low |
|  |  |  |  |  | volume of blood transfused | total | 2 | 20197 | WMD -0.21[-0.41,-0.01] | 0.0% | High | Moderate |
|  |  |  |  |  |  | TXA | 1 | 20127 | WMD -0.40[0.91,0.11] | N/A | High | Very low |
|  |  |  |  |  |  | aprotinin | 1 | 70 | WMD -0.17[-0.39,0.05] | N/A | High | Very low |

CS=cohort studies

CCS=case-control studies

## Appendix 9. Tabular presentation of discussion in included Meta-analysis.

| Dosage | Surgery | Author year | Type of Incl. study | AMSTAR | Outcome | Subgroup | No. of  incl. study | No. of  Patient | Evidence synthesis | I^2^ | Public-ation bias | GRADE  quality |
| --- | --- | --- | --- | --- | --- | --- | --- | --- | --- | --- | --- | --- |
| Inconsistent | TKA | Li 2020 | RCT | High | Total Blood Loss (ml) | TKA | 13 | 1197 | WMD 33.38 [19.24, 47.51] | 11.0% | Low | High |
|  |  |  |  |  | Transfusion rate | TKA | 25 | 2950 | OR 0.93 [0.69, 1.24] | 0.0% | Low | Moderate |
|  |  |  |  |  | All adverse event | TKA | 20 | 2594 | OR 1.00 [0.72, 1.39] | 0.0% | Low | Moderate |
|  |  |  |  |  | DVT | TKA | 10 | 1641 | OR 0.92 [0.44, 1.92] | 0.0% | Low | Moderate |
|  |  |  |  |  | PE | TKA | 3 | 342 | OR 1.02 [0.25, 4.20] | 0.0% | High | Low |
|  | | | | | | | | | | | | |
| IV 15mg/kg | THA | Zhang 2017 | RCT | High | total blood loss(ml) |  | 7 | 802 | WMD -146.29[-206.66,-85.92] | 83.0% | Low | Low |
|  |  |  |  |  | intraoperative blood loss(ml) |  | 5 | 654 | WMD -64.65[-74.75,-54.55] | 82.0% | High | Very low |
|  |  |  |  |  | transfusion rates |  | 7 | 668 | RR 0.41[0.27,0.63] | 84.0% | Low | Moderate |
|  |  |  |  |  | DVT |  | 8 | 950 | RR 1.22[0.52,2.89] | 0.0% | Low | Moderate |
|  |  |  |  |  | total blood loss(ml) |  | 7 | 802 | WMD -146.29[-206.66,-85.92] | 83.0% | Low | Low |
|  | | | | | | | | | | | | |
| Inconsistent | THA and TKA | Ye 2020 | RCT | High | total blood loss(ml) |  | 7 | 692 | WMD -3.67[-45.12,37.78] | 0.0% | Low | Low |
|  |  |  |  |  | decline in Hb(g/dl) |  | 7 | 854 | WMD -0.03[-0.11,0.05] | 0.0% | Low | Low |
|  |  |  |  |  | length of hospital stay(d) |  | 6 | 452 | WMD 0.09[-0.10,0.27] | 0.0% | Low | Low |
|  |  |  |  |  | DVT |  | 4 | 626 | OR 0.37[0.10,1.40] | 0.0% | High | Moderate |
|  |  |  |  |  | transfusion rate |  | 9 | 934 | OR 1.03[0.65,1.61] | 0.0% | Low | Moderate |
|  | | | | | | | | | | | | |
| Inconsistent | THA and TKA | Sun 2019 | RCT | High | total blood loss(ml)- IV vs topical | total | 14 |  | WMD 30.92[-28.40,90.25] | 87.0% | Low | very low |
|  |  |  |  |  |  | TKA | 10 |  | WMD 52.69 [-18.58,123.97] | 89.9% | Low | very low |
|  |  |  |  |  |  | THA | 4 |  | WMD -31.03[-156.16,94.10] | 70.3% | High | Very low |
|  |  |  |  |  | transfusion rate-IV vs topical | total | 17 |  | RR 1.08[0.78,1.50] | 0.0% | Low | Moderate |
|  |  |  |  |  |  | TKA | 12 |  | RR 1.25[0.80,1.96] | 8.3% | Low | Moderate |
|  |  |  |  |  |  | THA | 5 |  | RR 0.80[0.46,1.37] | 0.0% | High | Moderate |
|  |  |  |  |  | DVT-IV vs topical | total | 9 |  | RR 1.89[0.79,4.55] | 0.0% | Low | Moderate |
|  |  |  |  |  |  | TKA | 5 |  | RR 2.14[0.74,6.18] | 0.0% | High | High |
|  |  |  |  |  |  | THA | 4 |  | RR 1.45[0.30,6.93] | 0.0% | High | Low |
|  |  |  |  |  | total blood loss-combined vs single | total | 7 |  | WMD -198.07[-88.46,-307.67] | 92.3% | Low | Low |
|  |  |  |  |  |  | TKA | 4 |  | WMD -168.34[-85.44,-251.25] | 59.4% | High | Low |
|  |  |  |  |  |  | THA | 3 |  | WMD -210.36[-13.34,-407.39] | 96.3% | High | Low |
|  |  |  |  |  | transfusion rate-combined vs single | total | 7 |  | RR 0.40[0.24,0.68] | 0.0% | Low | High |
|  |  |  |  |  |  | TKA | 4 |  | RR 0.48[0.16,1.39] | 0.0% | High | High |
|  |  |  |  |  |  | THA | 3 |  | RR 0.38[0.20,0.69] | 0.0% | High | High |
|  |  |  |  |  | DVT-combined vs single | total | 6 |  | RR 1.25[0.43,3.70] | 0.0% | Low | Moderate |
|  |  |  |  |  |  | TKA | 3 |  | RR 0.34[0.04,3.23] | N/A | High | Moderate |
|  |  |  |  |  |  | THA | 3 |  | RR 1.85[0.54,6.25] | N/A | High | very low |
|  | | | | | | | | | | | | |
| Inconsistent | THA and TKA | Chen 2016 | RCT | High | transfusion rates | TKA | 12 | 1026 | OR 0.90 [0.58,1.40] | 30.0% | Low | Moderate |
|  |  |  |  |  |  | THA | 4 | 550 | OR 1.19[0.67,2.09] | 0.0% | High | Low |
|  |  |  |  |  | total blood loss(ml) | TKA | 8 | 688 | WMD -28.72[-195.97,138.54] | 97.0% | Low | Very low |
|  |  |  |  |  |  | THA | 4 | 550 | WMD 38.66[-38.97,116.30] | 60.0% | High | Very low |
|  |  |  |  |  | infection | TKA | 2 | 160 | OR 1.00[0.14,7.24] | 0.0% | High | Low |
|  |  |  |  |  |  | THA | 1 | 140 | OR 1.52[0.25,9.49] | N/A | High | Very low |
|  |  |  |  |  | DVT | TKA | 9 | 640 | OR 1.10[0.45,2.68] | 0.0% | Low | Moderate |
|  |  |  |  |  |  | THA | 3 | 383 | OR 0.23[0.05,1.10] | 0.0% | High | Moderate |
|  |  |  |  |  | PE | THA | 3 | 482 | OR 0.33[0.01,8.32] | N/A | High | Low |
|  | | | | | | | | | | | | |
| IV 15mg/kg | total joint arthroplasty | Zhang 2016 | RCT | High | total blood loss(ml)) | total | 6 | 643 | WMD -138.70[-196.14,-81.26] | 65.0% | Low | Moderate |
|  |  |  |  |  |  | TKA | 3 | 363 | WMD -189.75[-306.94,-72.56] | 70.0% | High | Low |
|  |  |  |  |  |  | THA | 3 | 280 | WMD -95.62[-129.63,-61.60] | 0.0% | High | Moderate |
|  |  |  |  |  | transfusion rates |  | 6 | 643 | RR 0.42[0.20,0.85] | 9.0% | Low | High |
|  |  |  |  |  | Thromboembolic complications |  | 3 | 424 | RR 1.00[0.28,3.63] | 0.0% | High | Low |
|  | | | | | | | | | | | | |
| IV 10-15mg/kg | spine surgery | Xiong 2020 | RCT | High | blood loss(ml) | total | 10 | 960 | WMD -57.24[-109.69,-4.80] | 94.0% | High | Very low |
|  |  |  |  |  |  | intraoperative | 4 | 336 | WMD -32.72[-129.17,63.72] | 97.0% | High | Very low |
|  |  |  |  |  |  | hidden | 3 | 266 | WMD -76.73[-178.30,24.84] | 94.0% | High | Very low |
|  |  |  |  |  | blood transfusion |  | 4 | 274 | RR 0.91[0.60,1.40] | 0.0% | High | Low |
|  | | | | | | | | | | | | |
| IV 10-20mg/kg | Types of surgeries | Heyns 2021 | RCT | High | blood transfusion | 10mg/kg | / | / | 0.50 [0.24,1.03] | / | Low | Moderate |
|  |  |  |  |  | blood transfusion | 15mg/kg | / | / | 0.26 [0.19,0.36] | / | Low | High |
|  |  |  |  |  | blood transfusion | 20mg/kg | / | / | 0.17[0.07,0.41] | / | Low | High |

TXA=tranexamic acid , AP=aprotinin

TKA= total knee arthroplasty , THA= total hip arthroplasty

RCT=Randomized controlled trial

## Appendix 10. AMSTAR 2 quality appraisal scores of included meta-analysis

| Study | 1 | 2 | 3 | 4 | 5 | 6 | 7 | 8 | 9 | 10 | 11 | 12 | 13 | 14 | 15 | 16 | Rate |
| --- | --- | --- | --- | --- | --- | --- | --- | --- | --- | --- | --- | --- | --- | --- | --- | --- | --- |
| Zhao 2019 | Yes | Partial Yes | Yes | Partial Yes | Yes | No | Yes | Yes | Yes | Yes | Yes | Yes | Yes | Yes | Yes | Yes | High |
| Huang 2013 | Yes | Partial Yes | Yes | Partial Yes | Yes | Yes | Partial Yes | Yes | Yes | Yes | Yes | Yes | Yes | Yes | Yes | Yes | High |
| Guo 2018 | Yes | Partial Yes | Yes | Yes | Yes | Yes | Partial Yes | Yes | Yes | No | Yes | Yes | Yes | Yes | Yes | Yes | High |
| Wu 2017 | Yes | Partial Yes | Yes | Yes | No | Yes | Partial Yes | Yes | Yes | Yes | Yes | Yes | Yes | Yes | Yes | Yes | High |
| Chen 2014 | Yes | Partial Yes | Yes | Yes | Yes | Yes | Partial Yes | Yes | Yes | No | Yes | Yes | Yes | Yes | Yes | Yes | High |
| Li 2020 | Yes | Partial Yes | Yes | Yes | Yes | Yes | Partial Yes | Yes | Yes | No | Yes | Yes | Yes | Yes | Yes | Yes | High |
| Sukeik 2019 | Yes | Partial Yes | Yes | Partial Yes | Yes | Yes | Partial Yes | Yes | Yes | No | Yes | Yes | Yes | Yes | Yes | Yes | High |
| Zhang H 2017 | Yes | Partial Yes | Yes | Yes | No | Yes | Partial Yes | Yes | Yes | Yes | Yes | Yes | Yes | Yes | Yes | Yes | High |
| Huang 2015 | Yes | Partial Yes | Yes | Yes | Yes | Yes | Yes | Yes | Yes | No | Yes | Yes | Yes | Yes | Yes | Yes | High |
| Ye 2020 | Yes | Partial Yes | Yes | Partial Yes | Yes | Yes | Partial Yes | Yes | Yes | No | Yes | Yes | Yes | Yes | Yes | Yes | High |
| Sun 2019 | Yes | Partial Yes | Yes | Yes | Yes | Yes | Yes | Yes | Yes | No | Yes | Yes | Yes | Yes | Yes | Yes | High |
| Chen 2016 | Yes | Partial Yes | Yes | Yes | Yes | Yes | Yes | Yes | Yes | No | Yes | Yes | Yes | Yes | Yes | Yes | High |
| Kirsch 2017 | Yes | Partial Yes | Yes | Yes | Yes | Yes | Partial Yes | Yes | Yes | No | Yes | Yes | Yes | Yes | Yes | Yes | High |
| Zhang XQ 2016 | Yes | Yes | Yes | Yes | Yes | Yes | Yes | Yes | Yes | No | Yes | Yes | Yes | Yes | Yes | Yes | High |
| Xiong 2020 | Yes | Partial Yes | Yes | Partial Yes | Yes | Yes | Yes | Yes | Yes | No | Yes | Yes | Yes | Yes | Yes | Yes | High |
| Li G 2017 | Yes | Partial Yes | Yes | Yes | Yes | Yes | Yes | Yes | Yes | No | Yes | Yes | Yes | Yes | Yes | Yes | High |
| Chen 2021 | Yes | Partial Yes | Yes | Partial Yes | Yes | Yes | Partial Yes | Yes | Yes | Yes | Yes | Yes | Yes | Yes | Yes | Yes | High |
| Du 2018 | Yes | Partial Yes | Yes | Partial Yes | Yes | Yes | Partial Yes | Yes | Yes | No | Yes | Yes | Yes | Yes | N/A | Yes | High |
| Luo 2019 | Yes | Yes | Yes | Yes | Yes | Yes | Yes | Yes | Yes | No | Yes | Yes | Yes | Yes | Yes | Yes | High |
| Zhou 2019 | Yes | Partial Yes | Yes | Partial Yes | Yes | Yes | Partial Yes | Yes | Yes | No | Yes | Yes | Yes | Yes | No | Yes | High |
| Zhang 2019 | Yes | Partial Yes | Yes | Yes | Yes | Yes | Partial Yes | Yes | Yes | No | Yes | Yes | Yes | Yes | N/A | Yes | High |
| Xiao 2019 | Yes | Partial Yes | Yes | Partial Yes | Yes | Yes | Partial Yes | Yes | Yes | No | Yes | Yes | Yes | Yes | Yes | Yes | High |
| Yao 2019 | Yes | Partial Yes | Yes | Yes | Yes | Yes | Partial Yes | Yes | Yes | No | Yes | Yes | Yes | Yes | Yes | Yes | High |
| Zhang 2019 | Yes | Partial Yes | Yes | Partial Yes | Yes | Yes | Partial Yes | Yes | Yes | Yes | Yes | Yes | Yes | Yes | Yes | Yes | High |
| Khaie 2019 | Yes | Yes | Yes | Yes | Yes | Yes | Yes | Yes | Yes | No | Yes | Yes | Yes | Yes | Yes | Yes | High |
| Guo 2019 | Yes | Partial Yes | Yes | Yes | Yes | Yes | Partial Yes | Yes | Yes | No | Yes | Yes | Yes | Yes | Yes | Yes | High |
| July 2020 | Yes | Partial Yes | Yes | Yes | Yes | Yes | Yes | Yes | Yes | No | Yes | Yes | Yes | Yes | Yes | Yes | High |
| Hu 2019 | Yes | Partial Yes | Yes | Partial Yes | Yes | Yes | Yes | Yes | Yes | No | Yes | Yes | Yes | Yes | Yes | Yes | High |
| Lu 2019 | Yes | Partial Yes | Yes | Partial Yes | Yes | No | Yes | Yes | Yes | No | Yes | Yes | Yes | Yes | Yes | Yes | Moderate |
| Bryant-Smith 2018 | Yes | Yes | Yes | Yes | No | Yes | Yes | Yes | Yes | Yes | Yes | Yes | Yes | Yes | Yes | Yes | High |
| Tsai 2020 | Yes | Partial Yes | Yes | Partial Yes | Yes | Yes | Partial Yes | Yes | Yes | No | Yes | Yes | Yes | Yes | N/A | Yes | High |
| Twum-Barimah 2020 | Yes | Yes | Yes | Yes | Yes | Yes | Partial Yes | Yes | Yes | No | Yes | Yes | Yes | Yes | Yes | Yes | High |
| Juliana 2016 | Yes | Yes | No | Yes | Yes | Yes | Yes | Yes | Yes | No | Yes | Yes | Yes | Yes | Yes | Yes | High |
| Chan 2012 | Yes | Partial Yes | Yes | Yes | Yes | Yes | Partial Yes | Yes | Yes | No | Yes | Yes | Yes | Yes | N/A | Yes | High |
| Longo 2018 | Yes | Yes | Yes | Yes | Yes | Yes | Yes | Yes | Yes | No | Yes | Yes | Yes | Yes | Yes | Yes | High |
| Ker 2015 | Yes | Partial Yes | Yes | Partial Yes | Yes | Yes | Yes | Yes | Yes | Yes | Yes | Yes | Yes | Yes | N/A | Yes | High |
| Montroy 2017 | Yes | Yes | Yes | Yes | Yes | Yes | Yes | Yes | Yes | No | Yes | Yes | Yes | Yes | Yes | Yes | High |
| Kang 2019 | Yes | Partial Yes | Yes | Partial Yes | Yes | No | Yes | Yes | Yes | No | Yes | Yes | Yes | Yes | N/A | Yes | Moderate |
| Kim 2019 | Yes | Partial Yes | Yes | Partial Yes | Yes | Yes | Yes | Yes | Yes | No | Yes | Yes | Yes | Yes | Yes | Yes | High |
| Juliana 2018 | Yes | Partial Yes | Yes | Partial Yes | Yes | Yes | Partial Yes | Yes | Yes | No | Yes | Yes | Yes | Yes | N/A | Yes | High |
| Joseph 2018 | Yes | Yes | Yes | Yes | Yes | Yes | Yes | Yes | Yes | Yes | Yes | Yes | Yes | Yes | Yes | Yes | High |
| Wang 2019 | Yes | Yes | Yes | Yes | Yes | Yes | Yes | Yes | Yes | No | Yes | Yes | Yes | Yes | Yes | Yes | High |
| Xia 2020 | Yes | Partial Yes | Yes | Partial Yes | Yes | Yes | Yes | Yes | Yes | No | Yes | Yes | Yes | Yes | N/A | Yes | High |
| Taeuber 2021 | Yes | Partial Yes | Yes | Yes | Yes | Yes | Yes | Yes | Yes | Yes | Yes | Yes | Yes | Yes | Yes | Yes | High |

## Appendix 11. Details of GRADE

A1. joint replacement surgery--TKA

| Surgery | Author Year | Outcome | No. of studies | Participants | Types of studies | Risk of  Bias | Inconsistency | Indirectness | Imprecision | Publication bias | Plausible  Confounding | Magnitude of effect | Dose-response gradient | Quality |
| --- | --- | --- | --- | --- | --- | --- | --- | --- | --- | --- | --- | --- | --- | --- |
| TKA | Guo 2018 | hemoglobin (Hb) drop | 3 | 515 | RCT | not Serious | no Serious Inconsistency | Not serious | no Serious Risk | Suspected | would not reduce effect | No | No | Moderate |
|  |  | hematocrit (Hct) | 2 | 99 | RCT | not Serious | no Serious Inconsistency | Not serious | no Serious Risk | Suspected | would not reduce effect | No | No | Moderate |
|  |  | drain output | 5 | 608 | RCT | not Serious | no Serious Inconsistency | Not serious | no Serious Risk | Suspected | would not reduce effect | No | No | Moderate |
|  |  | transfusion rate | 4 | 555 | RCT | not Serious | Very Serious Inconsistency | Not serious | no Serious Risk | Suspected | would not reduce effect | No | No | Very low |
|  |  | thromboembolic complications | 5 | 608 | RCT | not Serious | no Serious Inconsistency | Not serious | Serious Risk | Suspected | would not reduce effect | No | No | Low |
| TKA | Wu 2017 | transfusion requirements | 5 | 398 | RCT | not Serious | Serious Inconsistency | Not serious | Serious Risk | Suspected | would not reduce effect | Yes | No | Moderate |
|  |  | deep venous thrombosis (DVT) | 6 | 394 | RCT | not Serious | no Serious Inconsistency | Not serious | no Serious Risk | not Suspected | would not reduce effect | No | No | Moderate |
|  |  | total blood loss(ml) | 4 | 332 | RCT | not Serious | Very Serious Inconsistency | Not serious | no Serious Risk | Suspected | would not reduce effect | No | No | Very low |
|  |  | drainage volume(ml) | 4 | 328 | RCT | not Serious | Very Serious Inconsistency | Not serious | no Serious Risk | Suspected | would not reduce effect | No | No | Very low |
| TKA | Chen 2014 | total blood loss(ml) | 4 | 517 | RCT | not Serious | Very Serious Inconsistency | Not serious | no Serious Risk | Suspected | would not reduce effect | No | No | Very low |
|  |  | drainage blood loss(ml) | 4 | 298 | RCT | not Serious | Very Serious Inconsistency | Not serious | no Serious Risk | Suspected | would not reduce effect | No | No | Very low |
|  |  | Hb loss(g/dl) | 4 | 298 | RCT | not Serious | Serious Inconsistency | Not serious | no Serious Risk | Suspected | would not reduce effect | No | No | Low |
|  |  | blood transfusions rates | 6 | 647 | RCT | not Serious | no Serious Inconsistency | Not serious | Serious Risk | not Suspected | would not reduce effect | Yes | Yes | High |
|  |  | deep vein thrombosis (DVT) | 2 | 184 | RCT | not Serious | Inconsistency | Not serious | Serious Risk | Suspected | would not reduce effect | No | No | Very low |
| TKA | Li 2020 | Total Blood Loss (ml) | 13 | 1197 | RCT | not Serious | no Serious Inconsistency | Not serious | no Serious Risk | not Suspected | would not reduce effect | No | No | High |
|  |  | Drain Output (ml) | 17 | 1494 | RCT | not Serious | Very Serious Inconsistency | Not serious | no Serious Risk | not Suspected | would not reduce effect | No | No | Low |
|  |  | Hidden Blood Loss (ml) | 6 | 640 | RCT | not Serious | Serious Inconsistency | Not serious | Serious Risk | not Suspected | would not reduce effect | No | No | Low |
|  |  | Hemoglobin (Hb) Fall (mg/dl) | 19 | 1749 | RCT | not Serious | Very Serious Inconsistency | Not serious | Serious Risk | not Suspected | would not reduce effect | No | No | Very low |
|  |  | Transfusion rate | 25 | 2950 | RCT | not Serious | no Serious Inconsistency | Not serious | Serious Risk | not Suspected | would not reduce effect | No | No | Moderate |
|  |  | Complications (all) | 20 | 2594 | RCT | not Serious | no Serious Inconsistency | Not serious | Serious Risk | not Suspected | would not reduce effect | No | No | Moderate |
|  |  | DVT | 10 | 1641 | RCT | not Serious | no Serious Inconsistency | Not serious | Serious Risk | not Suspected | would not reduce effect | No | No | Moderate |
|  |  | PE | 3 | 342 | RCT | not Serious | no Serious Inconsistency | Not serious | Serious Risk | Suspected | would not reduce effect | No | No | Low |
|  |  | Wound complications | 14 | 1465 | RCT | not Serious | no Serious Inconsistency | Not serious | Serious Risk | not Suspected | would not reduce effect | No | No | Moderate |
|  |  | Other adverse events | 13 | 1899 | RCT | not Serious | no Serious Inconsistency | Not serious | Serious Risk | not Suspected | would not reduce effect | No | No | Moderate |
|  |  | Length of Stay (day) | 7 | 748 | RCT | not Serious | no Serious Inconsistency | Not serious | Serious Risk | not Suspected | would not reduce effect | No | No | Moderate |
|  |  | Tourniquet Time (min) | 9 | 816 | RCT | not Serious | no Serious Inconsistency | Not serious | Serious Risk | not Suspected | would not reduce effect | No | No | Moderate |

IV= intravenous

RCT=randomized controlled study

TKA=total knee arthroplasty

A2. joint replacement surgery--THA

| Surgery | Author Year | Outcome | No. of studies | Participants | | Types of studies | Risk of  Bias | Inconsistency | Indirectness | Imprecision | Publication bias | Plausible  Confounding | Magnitude of effect | Dose-response gradient | Quality |
| --- | --- | --- | --- | --- | --- | --- | --- | --- | --- | --- | --- | --- | --- | --- | --- |
| THA | Sukeik 2019 | wound complications | 25 | 1608 | RCT | | not Serious | no Serious Inconsistency | Not serious | no Serious Risk | not Suspected | would not reduce effect | No | No | High |
|  |  | intraoperative blood loss(ml) | 13 | 834 | RCT | | not Serious | Serious Inconsistency | Not serious | no Serious Risk | not Suspected | would not reduce effect | No | No | Moderate |
|  |  | postoperative blood loss(ml) | 15 | 1048 | RCT | | not Serious | Serious Inconsistency | Not serious | no Serious Risk | not Suspected | would not reduce effect | No | No | Moderate |
|  |  | total blood loss(ml) | 13 | / | RCT | | not Serious | Serious Inconsistency | Not serious | no Serious Risk | not Suspected | would not reduce effect | No | No | Moderate |
|  |  | blood transfusion | 19 | 1331 | RCT | | not Serious | Serious Inconsistency | Not serious | no Serious Risk | not Suspected | would not reduce effect | No | No | Moderate |
|  |  | deep venous thrombosis | 16 | 1432 | RCT | | not Serious | Serious Inconsistency | Not serious | no Serious Risk | not Suspected | would not reduce effect | No | No | Moderate |
|  |  | pulmonary embolisms | 7 | / | RCT | | not Serious | Serious Inconsistency | Not serious | no Serious Risk | not Suspected | would not reduce effect | No | No | Moderate |
|  |  | other complications | 25 | 1608 | RCT | | not Serious | Serious Inconsistency | Not serious | no Serious Risk | not Suspected | would not reduce effect | No | No | Moderate |
| THA | Chen S 2016 | total blood loss(ml) | 10 | 1277 | RCT, non-RCT | | Serious | Serious Inconsistency | Not serious | no Serious Risk | not Suspected | would not reduce effect | No | No | Low |
|  |  | total drain out(ml) | 6 | 791 | RCT, non-RCT | | Serious | Serious Inconsistency | Not serious | no Serious Risk | not Suspected | would not reduce effect | No | No | Low |
|  |  | drop in haemoglobin level | 11 | 1596 | RCT, non-RCT | | Serious | Serious Inconsistency | Not serious | no Serious Risk | not Suspected | would not reduce effect | No | No | Low |
|  |  | length of hospital stay | 6 | 1144 | RCT, non-RCT | | Serious | Serious Inconsistency | Not serious | no Serious Risk | not Suspected | would not reduce effect | No | No | Low |
|  |  | deep vein thrombosis | 6 | 1703 | RCT, non-RCT | | Serious | no Serious Inconsistency | Not serious | no Serious Risk | not Suspected | would not reduce effect | No | No | Low |
|  |  | pulmonary embolism | 2 | 1505 | RCT, non-RCT | | Serious | Serious Inconsistency | Not serious | no Serious Risk | Suspected | would not reduce effect | No | No | Very low |
|  |  | transfusion rate | 12 | 2764 | RCT, non-RCT | | Serious | no Serious Inconsistency | Not serious | no Serious Risk | not Suspected | would not reduce effect | No | No | Low |
| THA | Wang 2019 | total blood loss | 3 | 283 | RCT | | not Serious | Serious Inconsistency | Not serious | Serious Risk | Suspected | would not reduce effect | No | No | Very low |
|  |  | maximum hemoglobin drop | 3 | 283 | RCT | | not Serious | Serious Inconsistency | Not serious | Serious Risk | Suspected | would not reduce effect | No | No | Very low |
|  |  | Transfusion requirements | 4 | 391 | RCT | | not Serious | no Serious Inconsistency | Not serious | no Serious Risk | Suspected | would not reduce effect | No | No | Moderate |
|  |  | length of stay | 3 | 283 | RCT | | not Serious | Serious Inconsistency | Not serious | Serious Risk | Suspected | would not reduce effect | No | No | Very low |
|  |  | incidence of DVT | 4 | 391 | RCT | | not Serious | no Serious Inconsistency | Not serious | no Serious Risk | Suspected | would not reduce effect | Yes | No | High |
| THA | Zhang 2018 | need for transfusion | 5 | / | RCT | | not Serious | no Serious Inconsistency | Not serious | no Serious Risk | Suspected | would not reduce effect | No | No | Moderate |
|  |  | total blood loss(ml) | 5 | / | RCT | | not Serious | Serious Inconsistency | Not serious | no Serious Risk | Suspected | would not reduce effect | No | No | Low |
|  |  | hidden blood loss(ml) | 5 | / | RCT | | not Serious | Serious Inconsistency | Not serious | no Serious Risk | Suspected | would not reduce effect | No | No | Low |
|  |  | intraoperative blood loss(ml) | 4 | / | RCT | | not Serious | Serious Inconsistency | Not serious | no Serious Risk | Suspected | would not reduce effect | No | No | Very low |
|  |  | deep venous thrombosis | 5 | / | RCT | | not Serious | no Serious Inconsistency | Not serious | no Serious Risk | Suspected | would not reduce effect | No | No | Moderate |
|  |  | haematoma | 5 | / | RCT | | not Serious | no Serious Inconsistency | Not serious | no Serious Risk | Suspected | would not reduce effect | No | No | Moderate |
| THA | Zhang H 2017 | total blood loss(ml) | 7 | 802 | RCT | | not Serious | Serious Inconsistency | Not serious | no Serious Risk | not Suspected | would not reduce effect | No | No | Moderate |
|  |  | hemoglobin drop(g/dl) | 4 | 412 | RCT | | not Serious | Serious Inconsistency | Not serious | no Serious Risk | Suspected | would not reduce effect | No | No | Low |
|  |  | intraoperative blood loss(ml) | 5 | 654 | RCT | | not Serious | Serious Inconsistency | Not serious | no Serious Risk | Suspected | would not reduce effect | No | No | Low |
|  |  | the length of hospital stay(d) | 4 | 424 | RCT | | not Serious | no Serious Inconsistency | Not serious | no Serious Risk | Suspected | would not reduce effect | No | No | Low |
|  |  | need for transfusion | 7 | 668 | RCT | | not Serious | Serious Inconsistency | Not serious | no Serious Risk | not Suspected | would not reduce effect | Yes | No | High |
|  |  | the occurrence of deep venous thrombosis (DVT) | 8 | 950 | RCT | | not Serious | no Serious Inconsistency | Not serious | no Serious Risk | not Suspected | would not reduce effect | Yes | No | High |
|  |  | hidden blood loss(ml) | 3 | 248 | RCT | | not Serious | Serious Inconsistency | Not serious | no Serious Risk | Suspected | would not reduce effect | No | No | Low |
| THA&TKA | Ye 2020 | total blood loss(ml) | 7 | 692 | RCT | | not Serious | no Serious Inconsistency | Not serious | Serious Risk | not Suspected | would not reduce effect | No | No | Moderate |
|  |  | decline in hemoglobin(g/dl) | 7 | 854 | RCT | | not Serious | no Serious Inconsistency | Not serious | Serious Risk | not Suspected | would not reduce effect | No | No | Moderate |
|  |  | length of hospital stay(d) | 6 | 452 | RCT | | not Serious | no Serious Inconsistency | Not serious | Serious Risk | not Suspected | would not reduce effect | No | No | Moderate |
|  |  | incidence of DVT or IVT | 4 | 626 | RCT | | not Serious | no Serious Inconsistency | Not serious | no Serious Risk | Suspected | would not reduce effect | No | No | Moderate |
|  |  | transfusion rate | 9 | 934 | RCT | | not Serious | no Serious Inconsistency | Not serious | no Serious Risk | not Suspected | would not reduce effect | No | No | High |
| THA&TKA | Sun 2019 | IV between topical- total blood loss volume | 14 | unclear | RCT | | not Serious | Serious Inconsistency | Not serious | Serious Risk | not Suspected | would not reduce effect | No | No | Low |
|  |  | IV between topical-drain blood loss | 8 | unclear | RCT | | not Serious | Serious Inconsistency | Not serious | Serious Risk | not Suspected | would not reduce effect | No | No | Low |
|  |  | IV between topical-transfusion rate | 17 | unclear | RCT | | not Serious | no Serious Inconsistency | Not serious | no Serious Risk | not Suspected | would not reduce effect | No | No | High |
|  |  | IV between topical-postoperative haemoglobin level | 14 | unclear | RCT | | not Serious | Serious Inconsistency | Not serious | Serious Risk | not Suspected | would not reduce effect | No | No | Low |
|  |  | IV between topical-postoperative venous thromboembolism | 9 | unclear | RCT | | not Serious | no Serious Inconsistency | Not serious | no Serious Risk | not Suspected | would not reduce effect | No | No | High |
|  |  | IV between topical-length of hospital stay | 7 | unclear | RCT | | not Serious | Serious Inconsistency | Not serious | Serious Risk | not Suspected | would not reduce effect | No | No | Low |
|  |  | combined between single route-total blood loss volume | 7 | unclear | RCT | | not Serious | Serious Inconsistency | Not serious | no Serious Risk | not Suspected | would not reduce effect | No | No | Moderate |
|  |  | combined between single route-drain blood loss | 2 | unclear | RCT | | not Serious | Serious Inconsistency | Not serious | Serious Risk | Suspected | would not reduce effect | No | No | Very low |
|  |  | combined between single route-postoperative haemoglobin level | 2 | unclear | RCT | | not Serious | Serious Inconsistency | Not serious | Serious Risk | Suspected | would not reduce effect | No | No | Very low |
|  |  | combined between single route-haemoglobin decline | 6 | unclear | RCT | | not Serious | Serious Inconsistency | Not serious | no Serious Risk | not Suspected | would not reduce effect | No | No | Moderate |
|  |  | combined between single route-transfusion rate | 7 | unclear | RCT | | not Serious | no Serious Inconsistency | Not serious | no Serious Risk | not Suspected | would not reduce effect | Yes | No | High |
|  |  | combined between single route-postoperative venous thromboembolism | 6 | unclear | RCT | | not Serious | no Serious Inconsistency | Not serious | no Serious Risk | not Suspected | would not reduce effect | No | No | High |
|  |  | combined between single route-length of hospital stay | 4 | unclear | RCT | | not Serious | no Serious Inconsistency | Not serious | no Serious Risk | Suspected | would not reduce effect | No | No | Low |
|  |  | IV between topical-length of hospital stay (LOS) | 4 | unclear | RCT | | not Serious | no Serious Inconsistency | Not serious | Serious Risk | Suspected | would not reduce effect | No | No | Moderate |
|  |  | IV between topical-occurrence of venous thromboembolism (VTE) | 6 | unclear | RCT | | not Serious | no Serious Inconsistency | Not serious | no Serious Risk | not Suspected | would not reduce effect | No | No | High |
|  |  | IV between topical-decline in haemoglobin | 7 | unclear | RCT | | not Serious | Serious Inconsistency | Not serious | Serious Risk | not Suspected | would not reduce effect | No | No | Low |
| THA&TKA | Chen 2016 | transfusion rates | 16 | 1576 | RCT | | not Serious | no Serious Inconsistency | Not serious | no Serious Risk | not Suspected | would not reduce effect | No | No | High |
|  |  | total blood loss | 20 | unclear | RCT | | not Serious | no Serious Inconsistency | Not serious | Serious Risk | not Suspected | would not reduce effect | No | No | Moderate |
|  |  | total drain out | 20 | unclear | RCT | | not Serious | no Serious Inconsistency | Not serious | Serious Risk | not Suspected | would not reduce effect | No | No | Moderate |
|  |  | hidden blood loss | 20 | unclear | RCT | | not Serious | no Serious Inconsistency | Not serious | Serious Risk | not Suspected | would not reduce effect | No | No | Moderate |
|  |  | haemoglobin drop | 14 | 1468 | RCT | | not Serious | no Serious Inconsistency | Not serious | Serious Risk | not Suspected | would not reduce effect | No | No | Moderate |
|  |  | length of hospital stay | 5 | 591 | RCT | | not Serious | Serious Inconsistency | Not serious | Serious Risk | Suspected | would not reduce effect | No | No | Very low |
|  |  | complications | 20 | unclear | RCT | | not Serious | no Serious Inconsistency | Not serious | no Serious Risk | not Suspected | would not reduce effect | No | No | High |
| THA&TKA | Yu 2017 | total blood loss | 5 | 493 | RCT | | not Serious | no Serious Inconsistency | Not serious | no Serious Risk | Suspected | would not reduce effect | No | Yes | Moderate |
|  |  | need for transfusion | 4 | 393 | RCT | | not Serious | no Serious Inconsistency | Not serious | no Serious Risk | Suspected | would not reduce effect | Yes | No | High |
|  |  | hemoglobin drop | 4 | 393 | RCT | | not Serious | Serious Inconsistency | Not serious | no Serious Risk | Suspected | would not reduce effect | No | No | Moderate |
|  |  | incidence of deep venous thrombosis (DVT) | 4 | 393 | RCT | | not Serious | no Serious Inconsistency | Not serious | no Serious Risk | Suspected | would not reduce effect | No | No | Moderate |
|  |  | hematoma | 5 | 493 | RCT | | not Serious | no Serious Inconsistency | Not serious | no Serious Risk | Suspected | would not reduce effect | No | No | Moderate |
| THA&TKA | Alshryda 2014 | rate of blood transfusion | 9 | 1514 | RCT | | not Serious | no Serious Inconsistency | Not serious | no Serious Risk | not Suspected | would not reduce effect | No | Yes | High |
|  |  | deep venous thrombosis rate | 12 | 1947 | RCT | | not Serious | no Serious Inconsistency | Not serious | no Serious Risk | not Suspected | would not reduce effect | No | No | High |
| THA&TKA | Zhang XQ 2016 | total blood loss | 6 | 643 | RCT | | not Serious | Serious Inconsistency | Not serious | no Serious Risk | not Suspected | would not reduce effect | No | No | Moderate |
|  |  | transfusion rates | 6 | 643 | RCT | | not Serious | no Serious Inconsistency | Not serious | no Serious Risk | not Suspected | would not reduce effect | No | No | High |
|  |  | postoperative thromboembolic complications | 3 | 424 | RCT | | not Serious | no Serious Inconsistency | Not serious | no Serious Risk | Suspected | would not reduce effect | No | No | Moderate |

IV= intravenous

RCT=randomized controlled study

TKA=total knee arthroplasty

THA=total hip arthroplasty

B. Other orthopedic surgery

| Surgery | Author Year | Outcome | No. of studies | Participants | Types of studies | Risk of  Bias | Inconsistency | Indirectness | Imprecision | Publication bias | Plausible  Confounding | Magnitude of effect | Dose-response gradient | Quality |
| --- | --- | --- | --- | --- | --- | --- | --- | --- | --- | --- | --- | --- | --- | --- |
| hip fracture surgery | Xiao C 2019 | transfusion requirement | 11 | 892 | RCT | Not serious | Very Serious Inconsistency | Not serious | no Serious Risk | not Suspected | would not  reduce effect | No | No | Low |
|  |  | total blood loss | 6 | 416 | RCT | Not serious | Very Serious Inconsistency | Not serious | no Serious Risk | not Suspected | would not  reduce effect | No | No | Low |
|  |  | deep vein thrombosis | 10 | 854 | RCT | Not serious | no Serious Inconsistency | Not serious | Serious Risk | not Suspected | would not  reduce effect | No | No | Moderate |
|  |  | total thromboembolic events | 10 | 854 | RCT | Not serious | no Serious Inconsistency | Not serious | Serious Risk | not Suspected | would not  reduce effect | No | No | Moderate |
| spine surgery | Xiong 2020 | blood loss(ml) | 10 | 960 | RCT | Not serious | Very Serious Inconsistency | Not serious | no Serious Risk | not Suspected | would not  reduce effect | No | No | Low |
|  |  | hematocrit(%) | 4 | 412 | RCT | Not serious | Serious Inconsistency | Not serious | Serious Risk | Suspected | would not  reduce effect | No | No | Very low |
|  |  | hemoglobin level(g/L) | 5 | 328 | RCT | Not serious | no Serious Inconsistency | Not serious | Serious Risk | Suspected | would not  reduce effect | No | No | Low |
|  |  | fibrinogen(g/L) | 6 | 384 | RCT | Not serious | Serious Inconsistency | Not serious | Serious Risk | not Suspected | would not  reduce effect | No | No | Low |
|  |  | drainage volume(ml) | 4 | 234 | RCT | Not serious | no Serious Inconsistency | Not serious | Serious Risk | Suspected | would not  reduce effect | No | No | Low |
|  |  | prothrombin time(s) | 9 | 524 | RCT | Not serious | no Serious Inconsistency | Not serious | no Serious Risk | not Suspected | would not  reduce effect | No | No | High |
|  |  | activated partial thromboplastin time(s) | 6 | 316 | RCT | Not serious | Very Serious Inconsistency | Not serious | Serious Risk | not Suspected | would not  reduce effect | No | No | Very low |
|  |  | blood transfusion rate | 4 | 274 | RCT | Not serious | no Serious Inconsistency | Not serious | Serious Risk | Suspected | would not  reduce effect | No | No | Low |
| spine surgery | Chen J 2021 | intraoperative blood loss(ml) | 7 | 587 | RCT | Not serious | Serious Inconsistency | Not serious | no Serious Risk | not Suspected | would not  reduce effect | No | No | Moderate |
|  |  | postoperative drainage(ml) | 4 | 336 | RCT | Not serious | no Serious Inconsistency | Not serious | no Serious Risk | Suspected | would not  reduce effect | No | No | Moderate |
|  |  | total perioperative blood loss(ml) | 10 | 802 | RCT | Not serious | Serious Inconsistency | Not serious | no Serious Risk | not Suspected | would not  reduce effect | No | No | Moderate |
|  |  | postoperative hemoglobin | 6 | 492 | RCT | Not serious | no Serious Inconsistency | Not serious | no Serious Risk | not Suspected | would not  reduce effect | No | No | High |
|  |  | operation time(min) | 6 | 509 | RCT | Not serious | no Serious Inconsistency | Not serious | Serious Risk | not Suspected | would not  reduce effect | No | No | Moderate |
|  |  | length of hospital stay(d) | 5 | 426 | RCT | Not serious | Serious Inconsistency | Not serious | no Serious Risk | Suspected | would not  reduce effect | No | No | Low |
|  |  | blood transfusion rate | 5 | 471 | RCT | Not serious | no Serious Inconsistency | Not serious | no Serious Risk | Suspected | would not  reduce effect | No | No | Moderate |
|  |  | thrombotic complications | 4 | 351 | RCT | Not serious | no Serious Inconsistency | Not serious | Serious Risk | Suspected | would not  reduce effect | No | No | Low |
| TSA | Kirsch 2017 | change in hemoglobin(g/dL) | 5 | 632 | RCT, non-RCT | Not serious | no Serious Inconsistency | Not serious | no Serious Risk | Suspected | would not  reduce effect | No | No | Very low |
|  |  | drain output(ml) | 3 | 438 | RCT, non-RCT | Not serious | Very Serious Inconsistency | Not serious | no Serious Risk | Suspected | would not  reduce effect | No | No | Very low |
|  |  | transfusion requirements | 5 | 632 | RCT, non-RCT | Not serious | no Serious Inconsistency | Not serious | Serious Risk | Suspected | would not  reduce effect | Yes | No | Very low |
| spinal fusion surgery | Du 2018 | intraoperative blood loss | 6 | 394 | RCT | Not serious | no Serious Inconsistency | Not serious | no Serious Risk | not Suspected | would not  reduce effect | No | No | High |
|  |  | drain | 4 | 286 | RCT | Not serious | Serious Inconsistency | Not serious | no Serious Risk | Suspected | would not  reduce effect | No | No | Low |
|  |  | hemoglobin | 4 | 254 | RCT | Not serious | Serious Inconsistency | Not serious | no Serious Risk | Suspected | would not  reduce effect | No | No | Very low |
|  |  | transfusion | 3 | 231 | RCT | Not serious | no Serious Inconsistency | Not serious | no Serious Risk | Suspected | would not  reduce effect | Yes | No | High |
|  |  | hematocrit 1 day after surgery | 4 | 254 | RCT | Not serious | Very Serious Inconsistency | Not serious | no Serious Risk | Suspected | would not  reduce effect | No | No | Very low |
|  |  | Duration of hospitalization | 2 | 140 | RCT | Not serious | Serious Inconsistency | Not serious | no Serious Risk | Suspected | would not  reduce effect | No | No | Low |
| ICFS | Luo 2019 | mean total blood loss | 4 | 339 | RCT | Not serious | no Serious Inconsistency | Not serious | no Serious Risk | Suspected | would not  reduce effect | No | No | Moderate |
|  |  | intra-ostoperative visible blood loss | 4 | 339 | RCT | Not serious | no Serious Inconsistency | Not serious | no Serious Risk | Suspected | would not  reduce effect | No | No | Moderate |
|  |  | intra- and postoperative transfusion rate | 5 | 540 | RCT | Not serious | Serious Inconsistency | Not serious | no Serious Risk | Suspected | would not  reduce effect | No | No | Low |
|  |  | hidden blood loss | 3 | 267 | RCT | Not serious | no Serious Inconsistency | Not serious | no Serious Risk | Suspected | would not  reduce effect | No | No | Moderate |
|  |  | overall incidence of thrombotic event | 5 | 539 | RCT | Not serious | no Serious Inconsistency | Not serious | Serious Risk | Suspected | would not  reduce effect | No | No | Low |
|  |  | hemoglobin values on postoperative day 3 | 3 | 364 | RCT | Not serious | no Serious Inconsistency | Not serious | Serious Risk | Suspected | would not  reduce effect | No | No | Low |
|  |  | red blood cell infusion | 5 | 540 | RCT | Not serious | Serious Inconsistency | Not serious | no Serious Risk | Suspected | would not  reduce effect | No | No | Low |
|  |  | mortality on postoperative day 30 | 3 | 239 | RCT | Not serious | no Serious Inconsistency | Not serious | Serious Risk | Suspected | would not  reduce effect | No | No | Low |
| ICFS | Zhou X 2019 | postoperative blood loss | 3 | 314 | RCT | Not serious | no Serious Inconsistency | Not serious | no Serious Risk | Suspected | would not  reduce effect | No | No | Moderate |
|  |  | hidden blood loss | 2 | 177 | RCT | Not serious | no Serious Inconsistency | Not serious | no Serious Risk | Suspected | would not  reduce effect | No | No | Moderate |
|  |  | total blood loss | 4 | 309 | RCT | Not serious | Very Serious Inconsistency | Not serious | no Serious Risk | Suspected | would not  reduce effect | No | No | Very low |
|  |  | intraoperative blood loss | 4 | 309 | RCT | serious | Very Serious Inconsistency | Not serious | Serious Risk | Suspected | would not  reduce effect | No | No | Very low |
|  |  | transfusion | 8 | 836 | RCT | Not serious | no Serious Inconsistency | Not serious | no Serious Risk | not Suspected | would not  reduce effect | No | No | High |
|  |  | deep venous thrombosis | 5 | 539 | RCT | Not serious | no Serious Inconsistency | Not serious | Serious Risk | Suspected | would not  reduce effect | No | No | Low |
| PAO and HTB | Yao 2019 | total blood loss in PAO (ml) | 3 | 333 | RCT | Not serious | no Serious Inconsistency | Not serious | no Serious Risk | Suspected | would not  reduce effect | No | No | Moderate |
|  |  | total blood loss in HTO (ml) | 3 | 332 | RCT | Not serious | Very Serious Inconsistency | Not serious | no Serious Risk | Suspected | would not  reduce effect | No | No | Very low |
|  |  | hemoglobin decline | 5 | 565 | RCT | Not serious | Serious Inconsistency | Not serious | no Serious Risk | Suspected | would not  reduce effect | No | No | Low |
|  |  | transfusion rates | 6 | 665 | RCT | Not serious | Serious Inconsistency | Not serious | no Serious Risk | not Suspected | would not  reduce effect | Yes | No | High |
|  |  | wound complications | 5 | 565 | RCT | Not serious | no Serious Inconsistency | Not serious | Serious Risk | Suspected | would not  reduce effect | No | No | Low |
| major orthopedic surgery | Huang 2013 | intraoperative total blood loss(ml) | 21 | 1259 | RCT | Not serious | Very Serious Inconsistency | Not serious | no Serious Risk | not Suspected | would not  reduce effect | No | No | Low |
|  |  | total blood loss(ml) | 24 | 1696 | RCT | Not serious | Very Serious Inconsistency | Not serious | no Serious Risk | not Suspected | would not  reduce effect | No | No | Low |
|  |  | postoperative total blood loss(ml) | 19 | 1267 | RCT | Not serious | Very Serious Inconsistency | Not serious | no Serious Risk | not Suspected | would not  reduce effect | No | No | Low |
|  |  | blood units transfused per patient(U) | 11 | 917 | RCT | Not serious | Very Serious Inconsistency | Not serious | no Serious Risk | not Suspected | would not  reduce effect | No | No | Low |
|  |  | blood volumes of blood transfused per patient(ml) | 7 | 397 | RCT | Not serious | no Serious Inconsistency | Not serious | no Serious Risk | not Suspected | would not  reduce effect | No | No | High |
|  |  | transfusion and deep vein thrombosis | 44 | 2689 | RCT | Not serious | no Serious Inconsistency | Not serious | Serious Risk | not Suspected | would not  reduce effect | No | No | Moderate |
|  |  | transfusion requirements | 42 | 2649 | RCT | Not serious | no Serious Inconsistency | Not serious | no Serious Risk | not Suspected | would not  reduce effect | No | No | High |
| calcaneal fracture surgery | Zhang S 2019 | intraoperative blood loss(ml) | 4 | 283 | RCT | Not serious | Very Serious Inconsistency | Not serious | no Serious Risk | Suspected | would not  reduce effect | No | No | Very low |
|  |  | postoperative drainage volume(ml) | 6 | 409 | RCT | Not serious | Very Serious Inconsistency | Not serious | no Serious Risk | not Suspected | would not  reduce effect | No | No | Low |
|  |  | postoperative hemoglobin | 4 | 287 | RCT | Not serious | Very Serious Inconsistency | Not serious | no Serious Risk | Suspected | would not  reduce effect | No | No | Very low |
|  |  | postoperative prothrombin time(s) | 5 | 349 | RCT | Not serious | Serious Inconsistency | Not serious | Serious Risk | Suspected | would not  reduce effect | No | No | Very low |
|  |  | postoperative activated partial thromboplastin time(s) | 6 | 409 | RCT | Not serious | no Serious Inconsistency | Not serious | Serious Risk | not Suspected | would not  reduce effect | No | No | Moderate |
|  |  | incidence of complications | 6 | 389 | RCT | Not serious | no Serious Inconsistency | Not serious | no Serious Risk | not Suspected | would not  reduce effect | Yes | No | High |
|  |  | vascular adverse events | 2 | 163 | RCT | Not serious | no Serious Inconsistency | Not serious | Serious Risk | Suspected | would not  reduce effect | No | No | Low |

IV= intravenous

RCT=randomized controlled study

TSA=total shoulder arthroplasty

ICFS=intertrochanteric fracture surgery

PAO= periacetabular osteotomy

HTB= high tibial osteotomy

C. cerebral surgery.

| Surgery | Author Year | Outcome | No. of studies | Participants | Types of studies | Risk of  Bias | Inconsistency | Indirectness | Imprecision | Publication bias | Plausible  Confounding | Magnitude of effect | Dose-response gradient | Quality |
| --- | --- | --- | --- | --- | --- | --- | --- | --- | --- | --- | --- | --- | --- | --- |
| traumatic brain injury | July 2020 | mortality | 5 | 30262 | RCT | Not serious | no Serious Inconsistency | Not serious | no Serious Risk | Suspected | would not reduce effect | No | No | Moderate |
|  |  | hemorrhagic expansion | 4 | 938 | RCT | Not serious | no Serious Inconsistency | Not serious | no Serious Risk | Suspected | would not reduce effect | No | No | Moderate |
|  |  | neurosurgical intervention | 4 | 21135 | RCT | Not serious | no Serious Inconsistency | Not serious | Serious Risk | Suspected | would not reduce effect | No | No | Low |
|  |  | unfavourable Glasgow Outcome Scale | 3 | 918 | RCT | Not serious | no Serious Inconsistency | Not serious | Serious Risk | Suspected | would not reduce effect | No | No | Low |
|  |  | vascular occlusive events-total | 4 | 33625 | RCT | Not serious | no Serious Inconsistency | Not serious | Serious Risk | Suspected | would not reduce effect | No | No | Low |
|  |  | vascular occlusive events-DVT | 4 | 33625 | RCT | Not serious | no Serious Inconsistency | Not serious | Serious Risk | Suspected | would not reduce effect | No | No | Low |
|  |  | vascular occlusive events-PE | 4 | 33625 | RCT | Not serious | no Serious Inconsistency | Not serious | Serious Risk | Suspected | would not reduce effect | No | No | Low |
|  |  | vascular occlusive events-stroke | 4 | 33625 | RCT | Not serious | no Serious Inconsistency | Not serious | Serious Risk | Suspected | would not reduce effect | No | No | Low |
|  |  | vascular occlusive events-MI | 3 | 33387 | RCT | Not serious | no Serious Inconsistency | Not serious | Serious Risk | Suspected | would not reduce effect | No | No | Low |
| cerebral Hemorrhage | Hu 2020 | mortality | 20 | 10253 | RCT | Not serious | no Serious Inconsistency | Not serious | Serious Risk | not Suspected | would not reduce effect | No | No | Moderate |
|  |  | poor functional outcomes | 6 | 3874 | RCT | Not serious | no Serious Inconsistency | Not serious | Serious Risk | not Suspected | would not reduce effect | No | No | Moderate |
|  |  | hematoma expansion | 11 | 4336 | RCT | Not serious | Serious Inconsistency | Not serious | no Serious Risk | not Suspected | would not reduce effect | No | No | Moderate |
|  |  | hemorrhage volume | 4 | 2687 | RCT | Not serious | no Serious Inconsistency | Not serious | no Serious Risk | Suspected | would not reduce effect | No | No | Moderate |
|  |  | adverse events-ACS or MI | 1 | 2325 | RCT | serious | no Serious Inconsistency | Not serious | Serious Risk | Suspected | would not reduce effect | No | No | Very low |
|  |  | adverse events-DVT | 5 | 2978 | RCT | Not serious | no Serious Inconsistency | Not serious | Serious Risk | Suspected | would not reduce effect | No | No | Low |
|  |  | adverse events-PE | 4 | 3366 | RCT | Not serious | no Serious Inconsistency | Not serious | Serious Risk | Suspected | would not reduce effect | No | No | Low |
|  |  | adverse events-ischaemic stroke or TIA | 5 | 3601 | RCT | Not serious | Serious Inconsistency | Not serious | Serious Risk | Suspected | would not reduce effect | No | No | Very low |
|  |  | combimed thrombotic events | 3 | 2904 | RCT | Not serious | no Serious Inconsistency | Not serious | no Serious Risk | Suspected | would not reduce effect | No | No | Moderate |
|  |  | adverse events-hydrocephalus | 4 | 1091 | RCT | Not serious | no Serious Inconsistency | Not serious | Serious Risk | Suspected | would not reduce effect | No | No | Low |
|  |  | adverse events-seizure or convulsions | 1 | 2325 | RCT | serious | no Serious Inconsistency | Not serious | Serious Risk | Suspected | would not reduce effect | No | No | Very low |
| craniosynostosis open surgery | Lu 2019 | intraoperative RBC transfusion-ml/kg | 9 | 542 | RCT, CS | Not serious | Serious Inconsistency | Not serious | no Serious Risk | not Suspected | would not reduce effect | No | No | Very low |
|  |  | postoperative RBC transfusion | 5 | 408 | RCT, CS | Not serious | Serious Inconsistency | Not serious | no Serious Risk | Suspected | would not reduce effect | No | No | Very low |
|  |  | blood loss-ml/kg | 7 | 477 | RCT, CS | Not serious | Serious Inconsistency | Not serious | no Serious Risk | not Suspected | would not reduce effect | No | No | Very low |
|  |  | fresh frozen plasma transfusion | 3 | unclear | RCT, CS | Not serious | Serious Inconsistency | Not serious | Serious Risk | Suspected | would not reduce effect | No | No | Very low |
|  |  | crystalloid transfusion-ml/kg | 3 | unclear | RCT, CS | Not serious | Serious Inconsistency | Not serious | Serious Risk | Suspected | would not reduce effect | No | No | Very low |
|  |  | operation time | 7 | unclear | RCT, CS | Not serious | Serious Inconsistency | Not serious | Serious Risk | not Suspected | would not reduce effect | No | No | Very low |
|  |  | length of stay-ICU | 3 | unclear | RCT, CS | Not serious | no Serious Inconsistency | Not serious | Serious Risk | Suspected | would not reduce effect | No | No | Very low |
|  |  | length of stay-hospital | 6 | unclear | RCT, CS | Not serious | Serious Inconsistency | Not serious | Serious Risk | not Suspected | would not reduce effect | No | No | Very low |
|  |  | complications | 6 | unclear | RCT, CS | Not serious | no Serious Inconsistency | Not serious | Serious Risk | not Suspected | would not reduce effect | No | No | Very low |
|  |  | Urinary output | 3 | unclear | RCT, CS | Not serious | no Serious Inconsistency | Not serious | Serious Risk | Suspected | would not reduce effect | No | No | Very low |
|  |  | hermatocrit | 4 | unclear | RCT, CS | Not serious | Very Serious Inconsistency | Not serious | Serious Risk | Suspected | would not reduce effect | No | No | Very low |

IV=intravenous

CS=comparative study

RCT=randomized controlled study

D. cardiac surgery

| Surgery | Author Year | Outcome | No. of studies | Participants | Types of studies | Risk of  Bias | Inconsistency | Indirectness | Imprecision | Publication bias | Plausible  Confounding | Magnitude of effect | Dose-response gradient | Quality |
| --- | --- | --- | --- | --- | --- | --- | --- | --- | --- | --- | --- | --- | --- | --- |
| CABG | Zhang 2019 | postoperative cerebrovascular acident | 22 | 6775 | RCT | no Serious Risk | no Serious Inconsistency | no Serious  Indirectness | Serious  risk | not Suspected | would not  reduce effect | No | No | Moderate |
|  |  | seizures | 4 | 4911 | RCT | no Serious Risk | no Serious Inconsistency | no Serious  Indirectness | no Serious  Risk | Suspected | would not  reduce effect | Yes | No | High |
|  |  | operation for bleeding | 16 | 6259 | RCT | no Serious Risk | no Serious Inconsistency | no Serious  Indirectness | no Serious  Risk | not Suspected | would not  reduce effect | Yes | No | High |
|  |  | postoperative mortality | 17 | 6259 | RCT | no Serious Risk | no Serious Inconsistency | no Serious  Indirectness | Serious  risk | not Suspected | would not  reduce effect | No | No | Moderate |
|  |  | myocardial infarction | 23 | 6714 | RCT | no Serious Risk | no Serious Inconsistency | no Serious  Indirectness | Serious  risk | not Suspected | would not  reduce effect | No | No | Moderate |
|  |  | acute renal insufficiency | 14 | 5954 | RCT | no Serious Risk | no Serious Inconsistency | no Serious  Indirectness | Serious  risk | not Suspected | would not  reduce effect | No | No | Moderate |
|  |  | allogeneic transfusions | 11 | 5360 | RCT | no Serious Risk | Very Serious Inconsistency | no Serious  Indirectness | no Serious  Risk | not Suspected | would not  reduce effect | No | No | Low |
|  |  | 24-h postoperative chest tube drainage | 16 | 6247 | RCT | no Serious Risk | Serious Inconsistency | no Serious  Indirectness | no Serious  Risk | not Suspected | would not  reduce effect | No | No | Moderate |
| cardiac surgery | Khaie 2019 | adverse events-TXA vs placebo/no intervention | 44 | 9896 | RCT | no Serious Risk | no Serious Inconsistency | no Serious  Indirectness | Serious  risk | not Suspected | would not  reduce effect | No | No | Moderate |
|  |  | adverse events-TXA vs ACA | 4 | unclear | RCT | no Serious Risk | Serious Inconsistency | no Serious  Indirectness | Serious  risk | Suspected | would not  reduce effect | No | No | Very low |
|  |  | adverse events-TXA vs aprotinin | 21 | unclear | RCT | no Serious Risk | Serious Inconsistency | no Serious  Indirectness | Serious  risk | not Suspected | would not  reduce effect | No | No | Low |
| cardiac surgery | Guo 2019 | transfusion rate | 31 | 8925 | RCT | no Serious Risk | no Serious Inconsistency | no Serious  Risk | no Serious  Risk | not Suspected | would not  reduce effect | No | No | High |
|  |  | transfusion volume-all patients | 10 | 2105 | RCT | no Serious Risk | Very Serious Inconsistency | no Serious  Risk | no Serious  Risk | not Suspected | would not  reduce effect | No | No | Low |
|  |  | transfusion volume-transfused patients | 14 | 6610 | RCT | no Serious Risk | Very Serious Inconsistency | no Serious  Risk | no Serious  Risk | not Suspected | would not  reduce effect | No | No | Low |
|  |  | post-operative blood loss | 44 | 5560 | RCT | no Serious Risk | Very Serious Inconsistency | no Serious  Risk | no Serious  Risk | not Suspected | would not  reduce effect | No | No | Low |
|  |  | re-operation rate | 32 | 8937 | RCT | no Serious Risk | no Serious Inconsistency | no Serious  Risk | no Serious  Risk | not Suspected | would not  reduce effect | No | No | High |
|  |  | post-operative complications-seizure | 5 | 5807 | RCT | no Serious Risk | no Serious Inconsistency | no Serious  Risk | no Serious  Risk | Suspected | would not  reduce effect | Yes | Yes | High |
|  |  | post-operative complications-mortality | 29 | 8907 | RCT | no Serious Risk | no Serious Inconsistency | no Serious  Risk | Serious  risk | not Suspected | would not  reduce effect | No | No | High |
|  |  | post-operative complications-stroke | 32 | 9257 | RCT | no Serious Risk | no Serious Inconsistency | no Serious  Risk | Serious  risk | not Suspected | would not  reduce effect | No | No | High |
|  |  | post-operative complications-myocardial infraction | 32 | 8688 | RCT | no Serious Risk | no Serious Inconsistency | no Serious  Risk | Serious  risk | not Suspected | would not  reduce effect | No | No | High |
|  |  | post-operative complications-pulmonary embolism | 18 | 6587 | RCT | no Serious Risk | no Serious Inconsistency | no Serious  Risk | Serious  risk | not Suspected | would not  reduce effect | No | No | High |
|  |  | post-operative complications-renal dysfunction | 19 | 7210 | RCT | no Serious Risk | no Serious Inconsistency | no Serious  Risk | Serious  risk | not Suspected | would not  reduce effect | No | No | High |

IV=intravenous

CABG=coronary artery bypass grafting surgery

RCT=randomized controlled study

E. nasal surgery

| Surgery | Author Year | Outcome | No. of studies | Participants | Types of studies | Risk of  Bias | Inconsistency | Indirectness | Imprecision | Publication bias | Plausible  Confounding | Magnitude of effect | Dose-response gradient | Quality |
| --- | --- | --- | --- | --- | --- | --- | --- | --- | --- | --- | --- | --- | --- | --- |
| ESS | Kim 2019 | intraoperative blood loss | 7 | 562 | RCT | no Serious Risk | no Serious Inconsistency | "no Serious | no Serious  Risk | not Suspected | would not  reduce effect | No | No | High |
|  |  | surgical field score | 5 | 332 | RCT | no Serious Risk | no Serious Inconsistency | Indirectness" | no Serious  Risk | Suspected | would not  reduce effect | No | No | Moderate |
|  |  | operative time | 4 | 232 | RCT | no Serious Risk | no Serious Inconsistency | "no Serious | no Serious  Risk | Suspected | would not  reduce effect | No | No | Moderate |
|  |  | intraoperative blood pressure | 2 | 198 | RCT | no Serious Risk | Serious Inconsistency | Indirectness" | Serious  Risk | Suspected | would not  reduce effect | No | No | Low |
|  |  | postoperative nausea and vomiting | 5 | 434 | RCT | no Serious Risk | Serious Inconsistency | "no Serious | Serious  Risk | Suspected | would not  reduce effect | No | No | Low |
|  |  | postoperative thrombotic accident | 4 | 374 | RCT | no Serious Risk | Serious Inconsistency | Indirectness" | Serious  Risk | Suspected | would not  reduce effect | No | No | Low |
|  |  | Coagulation function | 3 | 254 | RCT | no Serious Risk | Serious Inconsistency | "no Serious | Serious  Risk | Suspected | would not  reduce effect | No | No | Low |
| sinus surgery | Kang 2019 | intraoperative blood loss | 4 | 226 | RCT | no Serious Risk | no Serious Inconsistency | no Serious  Indirectness | no Serious  Risk | Suspected | would not  reduce effect | No | No | Moderate |
|  |  | surgical field score | 4 | 226 | RCT | no Serious Risk | Serious Inconsistency | no Serious  Indirectness | no Serious  Risk | Suspected | would not  reduce effect | No | No | Low |
|  |  | operative time | 2 | 110 | RCT | no Serious Risk | no Serious Inconsistency | no Serious  Indirectness | Serious  Risk | Suspected | would not  reduce effect | No | No | Low |
|  |  | intraoperative blood pressure | NP | NP | RCT | no Serious Risk | Very Serious Inconsistency | no Serious  Indirectness | Serious  Risk | Suspected | would not  reduce effect | No | No | Very low |
|  |  | postoperative nausea and vomiting | 2 | 110 | RCT | no Serious Risk | Serious Inconsistency | no Serious  Indirectness | Serious  Risk | Suspected | would not  reduce effect | No | No | Low |
|  |  | postoperative thrombotic accident | 2 | 110 | RCT | no Serious Risk | Serious Inconsistency | no Serious  Indirectness | Serious  Risk | Suspected | would not  reduce effect | No | No | Low |
|  |  | Coagulation function | 2 | 110 | RCT | no Serious Risk | Serious Inconsistency | no Serious  Indirectness | Serious  Risk | Suspected | would not  reduce effect | No | No | Low |
| rhinoplasty | Vasconcellos 2018 | intraoperative blood loss | 4 | 246 | RCT | no Serious Risk | Very Serious Inconsistency | no Serious  Indirectness | no Serious  Risk | Suspected | would not  reduce effect | No | No | Very low |
|  |  | Eyelid edema scores | unclear | unclear | RCT | no Serious Risk | no Serious Inconsistency | no Serious  Indirectness | Serious  Risk | Suspected | would not  reduce effect | No | No | Moderate |
|  |  | Postrhinoplasty ecchymosis scores | unclear | unclear | RCT | no Serious Risk | no Serious Inconsistency | no Serious  Indirectness | Serious  Risk | Suspected | would not  reduce effect | No | No | Moderate |
| epistaxis | Joseph 2018 | re-bleeding proportion | 3 | 225 | RCT | no Serious Risk | no Serious Inconsistency | no Serious  Indirectness | no Serious  Risk | Suspected | would not  reduce effect | No | No | Moderate |
|  |  | stop initial bleeding within 30 mins | 1 | 68 | RCT | no Serious Risk | Serious Inconsistency | no Serious  Indirectness | Serious  Risk | Suspected | would not  reduce effect | No | No | Very low |
|  |  | transfusion requirement | 1 | 89 | RCT | no Serious Risk | Serious Inconsistency | no Serious  Indirectness | Serious  Risk | Suspected | would not  reduce effect | No | No | Very low |
|  |  | compare to other haemostatic agents | 3 | 460 | RCT | no Serious Risk | no Serious Inconsistency | no Serious  Indirectness | no Serious  Risk | Suspected | would not  reduce effect | No | No | Moderate |

ESS= endoscopic sinus surgery

IV= intravenous

RCT=randomized controlled study

F. obstetrics and gynecological surgery

| Surgery | Author Year | Outcome | No. of studies | Participants | Types of studies | Risk of  Bias (sensitivity) | Inconsistency (heterogeneity) | Indirectness | | Imprecision (95% CI) | Publication bias | Plausible  Confounding | Magnitude of effect (RR<0.5 >2) | Dose-response gradient | Quality |
| --- | --- | --- | --- | --- | --- | --- | --- | --- | --- | --- | --- | --- | --- | --- | --- |
| caesarean section | Wang 2019 | intraoperative blood loss | 14 | 1665 | RCT | no Serious Risk | Serious Inconsistency | no Serious  Indirectness | no Serious  Risk | | suspected | would not  reduce effect | No | No | Very low |
|  |  | postoperative blood loss | 12 | 1398 | RCT | no Serious Risk | Serious Inconsistency | no Serious  Indirectness | no Serious  Risk | | not suspected | would not  reduce effect | No | No | Low |
|  |  | total blood loss | 11 | 2777 | RCT | no Serious Risk | Serious Inconsistency | no Serious  Indirectness | no Serious  Risk | | suspected | would not  reduce effect | No | No | Very low |
|  |  | transfusion requirement | 9 | 1472 | RCT | no Serious Risk | Serious Inconsistency | no Serious  Indirectness | no Serious  Risk | | not suspected | would not  reduce effect | Yes | No | High |
|  |  | hemoglobin drop | 9 | 1807 | RCT | no Serious Risk | Serious Inconsistency | no Serious  Indirectness | no Serious  Risk | | suspected | would not  reduce effect | No | No | Very low |
|  |  | hematocrit drop | 3 | 1053 | RCT | no Serious Risk | Serious Inconsistency | no Serious  Indirectness | no Serious  Risk | | suspected | would not  reduce effect | No | No | Very low |
|  |  | additional uterotonic agents use | 7 | 1900 | RCT | no Serious Risk | no Serious Inconsistency | no Serious  Indirectness | no Serious  Risk | | not suspected | would not  reduce effect | Yes | No | High |
|  |  | massive hemorrhage | 8 | 2234 | RCT | no Serious Risk | Serious Inconsistency | no Serious  Indirectness | no Serious  Risk | | not suspected | would not  reduce effect | Yes | No | High |
| vaginal delivery | Xia 2020 | total blood loss | 4 | 4579 | RCT | no Serious Risk | Serious Inconsistency | no Serious  Indirectness | no Serious  Risk | | suspected | would not  reduce effect | No | No | Very low |
|  |  | intraoperative blood loss | 3 | 4140 | RCT | no Serious Risk | Serious Inconsistency | no Serious  Indirectness | no Serious  Risk | | suspected | would not  reduce effect | No | No | Moderate |
|  |  | postoperative blood loss | 2 | 301 | RCT | no Serious Risk | Serious Inconsistency | no Serious  Indirectness | no Serious  Risk | | suspected | would not  reduce effect | No | No | Moderate |
|  |  | postpartum hemorrhage | 4 | 4579 | RCT | no Serious Risk | Serious Inconsistency | no Serious  Indirectness | no Serious  Risk | | suspected | would not  reduce effect | Yes | No | Moderate |
|  |  | severe postpartum hemorrhage | 3 | 4398 | RCT | no Serious Risk | Serious Inconsistency | no Serious  Indirectness | Serious  Risk | | suspected | would not  reduce effect | No | No | Low |
|  |  | transfusion requirement | 2 | 4278 | RCT | no Serious Risk | Serious Inconsistency | no Serious  Indirectness | Serious  Risk | | suspected | would not  reduce effect | No | No | Low |
|  |  | nausea or vomiting | 2 | 4020 | RCT | no Serious Risk | Serious Inconsistency | no Serious  Indirectness | no Serious  Risk | | suspected | would not  reduce effect | Yes | No | High |
|  |  | nausea | 2 | 4450 | RCT | no Serious Risk | Serious Inconsistency | no Serious  Indirectness | no Serious  Risk | | suspected | would not  reduce effect | Yes | No | High |
|  |  | vomiting | 2 | 4430 | RCT | no Serious Risk | Serious Inconsistency | no Serious  Indirectness | no Serious  Risk | | suspected | would not  reduce effect | Yes | No | High |
|  |  | dizziness | 2 | 4631 | RCT | no Serious Risk | Serious Inconsistency | no Serious  Indirectness | Serious  Risk | | suspected | would not  reduce effect | No | No | Low |
|  |  | photopsia | 1 | 4072 | RCT | no Serious Risk | Serious Inconsistency | no Serious  Indirectness | Serious  Risk | | suspected | would not  reduce effect | No | No | Low |

IV= intravenous

RCT=randomized controlled study

G. other types of surgery

| Surgery | Author Year | Outcome | No. of studies | Participants | Types of studies | Risk of  Bias | Inconsistency | Indirectness | Imprecision | Publication bias | Plausible  Confounding | Magnitude of effect | Dose-response gradient | Quality |
| --- | --- | --- | --- | --- | --- | --- | --- | --- | --- | --- | --- | --- | --- | --- |
| hemoptysis | Tsai 2020 | bleeding duration | 2 | 70 | RCT | serious | no Serious Inconsistency | Not serious | no Serious  Risk | Suspected | would not  reduce effect | No | No | Moderate |
|  |  | hemoptysis resolution | 3 | 117 | RCT | Not serious | Serious Inconsistency | Not serious | no Serious  Risk | Suspected | would not  reduce effect | No | No | Low |
|  |  | bleeding volume | 2 | 90 | RCT | Not serious | Serious Inconsistency | Not serious | no Serious  Risk | Suspected | would not  reduce effect | No | No | Low |
|  |  | further intervention risk | 2 | 113 | RCT | Not serious | no Serious Inconsistency | Not serious | no Serious  Risk | Suspected | would not  reduce effect | Yes | No | High |
|  |  | length of hospital stay | 2 | 113 | RCT | serious | no Serious Inconsistency | Not serious | no Serious  Risk | Suspected | would not  reduce effect | No | No | Moderate |
|  |  | adverse events | 2 | 70 | RCT | Not serious | no Serious Inconsistency | Not serious | no Serious  Risk | Suspected | would not  reduce effect | Yes | No | Moderate |
| upper gastrointestinal bleeding | Twum-Barimah 2020 | mortality | 10 | 2013 | RCT | Not serious | no Serious Inconsistency | Not serious | no Serious  Risk | not Suspected | would not  reduce effect | No | No | High |
|  |  | re-bleeding | 8 | 1750 | RCT | Not serious | no Serious Inconsistency | Not serious | Serious  Risk | not Suspected | would not  reduce effect | No | No | Moderate |
|  |  | surgical interventions | 9 | 1863 | RCT | Not serious | Serious Inconsistency | Not serious | Serious  Risk | not Suspected | would not  reduce effect | No | No | Low |
|  |  | blood transfusions | 8 | 1763 | RCT | Not serious | no Serious Inconsistency | Not serious | Serious  Risk | not Suspected | would not  reduce effect | No | No | Moderate |
|  |  | tromboembolic events | 6 | 1041 | RCT | Not serious | Serious Inconsistency | Not serious | Serious  Risk | not Suspected | would not  reduce effect | No | No | Moderate |
|  |  | thrombophlebtis | 2 | 354 | RCT | Not serious | no Serious Inconsistency | Not serious | Serious  Risk | Suspected | would not  reduce effect | Yes | No | Moderate |
| prostate surgery | Longo 2018 | intraoperative blood loss | 6 | 738 | RCT | Not serious | Very Serious Inconsistency | Not serious | no Serious  Risk | not Suspected | would not  reduce effect | No | No | Low |
|  |  | transfusion rate | 7 | 818 | RCT | Not serious | no Serious Inconsistency | Not serious | no Serious  Risk | not Suspected | would not  reduce effect | No | No | High |
|  |  | hemoglobin levels after 24h of surgery | 4 | 526 | RCT | Not serious | Very Serious Inconsistency | Not serious | Serious  Risk | Suspected | would not  reduce effect | No | No | Very low |
|  |  | thromboembolic events | 4 | 462 | RCT | Not serious | no Serious Inconsistency | Not serious | Serious  Risk | Suspected | would not  reduce effect | Yes | No | Low |
| minor oral surgery | Vasconcellos 2017 | bleeding after minor oral surgeries | 5 | 252 | RCT | Not serious | no Serious Inconsistency | Not serious | no Serious  Risk | Suspected | would not  reduce effect | Extremely Yes | No | High |
| tonsillectomy | Chan 2012 | blood loss | 2 | 180 | RCT | Not serious | no Serious Inconsistency | Not serious | no Serious Risk | Suspected | would not reduce effect | No | No | Moderate |
|  |  | post-operative haemorrhage | 5 | 1670 | RCT | Not serious | no Serious Inconsistency | Not serious | Serious Risk | Suspected | would not reduce effect | No | No | Low |
|  |  | duration of haemorrhage | 1 | 38 | RCT | serious | no Serious Inconsistency | Not serious | no Serious Risk | Suspected | would not reduce effect | No | No | Very low |
|  |  | further interventions | 2 | 27 | RCT | Not serious | no Serious Inconsistency | Not serious | Serious Risk | Suspected | would not reduce effect | No | No | Very low |
| any medical disciplines | Taeuber 2021 | total thromboembolic events | 176 | 65900 | RCT | Not serious | no Serious Inconsistency | Not serious | no Serious Risk | not Suspected | would not reduce effect | No | No | High |
|  |  | mortality | 63 | 55305 | RCT | Not serious | Serious Inconsistency | Not serious | no Serious Risk | not Suspected | would not reduce effect | No | No | Moderate |
|  |  | nonbleeding mortality | 48 | 46619 | RCT | Not serious | Serious Inconsistency | Not serious | no Serious Risk | not Suspected | would not reduce effect | No | No | Low |
|  |  | bleeding mortality | 49 | 46702 | RCT | Not serious | Serious Inconsistency | Not serious | no Serious Risk | not Suspected | would not reduce effect | No | No | Moderate |
| acute traumatic injury | Ker 2015 | antifibrinolytics versus control-mortality | 3 | 20437 | RCT | Not serious | no Serious Inconsistency | Not serious | no Serious Risk | Suspected | would not  reduce effect | No | No | Moderate |
|  |  | myocardial infarction | 2 | 20437 | RCT | Not serious | no Serious Inconsistency | Not serious | no Serious Risk | Suspected | would not  reduce effect | No | No | Moderate |
|  |  | stroke | 2 | 20437 | RCT | Not serious | no Serious Inconsistency | Not serious | no Serious Risk | Suspected | would not  reduce effect | No | No | Low |
|  |  | deep vein thrombosis | 2 | 20437 | RCT | Not serious | no Serious Inconsistency | Not serious | no Serious Risk | Suspected | would not  reduce effect | No | No | Low |
|  |  | pulmonary embolism | 2 | 20437 | RCT | Not serious | no Serious Inconsistency | Not serious | no Serious Risk | Suspected | would not  reduce effect | No | No | Low |
|  |  | surgical intervention | 3 | 20437 | RCT | Not serious | no Serious Inconsistency | Not serious | Serious Risk | Suspected | would not  reduce effect | No | No | Low |
|  |  | blood transfusion | 2 | 20367 | RCT | Not serious | no Serious Inconsistency | Not serious | Serious Risk | Suspected | would not  reduce effect | No | No | Low |
|  |  | volume of blood transfused | 2 | 20197 | RCT | Not serious | no Serious Inconsistency | Not serious | no Serious Risk | Suspected | would not  reduce effect | No | No | Moderate |
| orthognathic surgery | Zhao 2019 | intraoperative blood loss | 10 | 655 | RCT | Not serious | Serious Inconsistency | Not serious | no Serious Risk | not Suspected | would not reduce effect | No | Yes | High |
|  |  | hematocrit | 4 | 421 | RCT | Not serious | Serious Inconsistency | Not serious | no Serious Risk | Suspected | would not reduce effect | No | No | Low |
|  |  | operation time | 8 | 655 | RCT | Not serious | no Serious Inconsistency | Not serious | no Serious Risk | not Suspected | would not reduce effect | No | No | Moderate |
|  |  | quality of surgical field | 2 | 250 | RCT | Not serious | Serious Inconsistency | Not serious | no Serious Risk | Suspected | would not reduce effect | No | No | Low |
|  |  | transfusion rates | 7 | 463 | RCT | Not serious | no Serious Inconsistency | Not serious | no Serious Risk | not Suspected | would not reduce effect | Yes | No | High |
|  |  | intraoperative blood loss | 10 | 655 | RCT | Not serious | Serious Inconsistency | Not serious | no Serious Risk | not Suspected | would not reduce effect | No | Yes | High |
| types of surgeries | Heyns 2021 | blood transfussion(10mg/kg) | \ | \ | RCT | Not serious | no Serious Inconsistency | Not serious | no Serious  Risk | Suspected | would not  reduce effect | No | Yes | High |
|  |  | blood transfussion(15mg/kg) | \ | \ | RCT | Not serious | no Serious Inconsistency | Not serious | no Serious  Risk | Suspected | would not  reduce effect | Yes | Yes | High |
|  |  | blood transfussion(20mg/kg) | \ | \ | RCT | Not serious | no Serious Inconsistency | Not serious | no Serious  Risk | Suspected | would not  reduce effect | Yes | Yes | High |

CS= cohort study

CCS=case-control study

IV=intravenous

RCT=randomized controlled study

H. complex antifibrinolytic agents

| Surgery | Author Year | Outcome | No. of studies | Participants | Types of studies | Risk of  Bias | Inconsistency | Indirectness | Imprecision | Publication bias | Plausible  Confounding | Magnitude of effect | Dose-response gradient | Quality |
| --- | --- | --- | --- | --- | --- | --- | --- | --- | --- | --- | --- | --- | --- | --- |
| spine surgery | Li G 2017 | total blood loss | 13 | 943 | RCT | Not serious | Very Serious Inconsistency | Not serious | no Serious Risk | not Suspected | would not  reduce effect | No | No | Low |
|  |  | intra-operative blood loss | 13 | 894 | RCT | Not serious | Serious Inconsistency | Not serious | no Serious Risk | not Suspected | would not  reduce effect | No | No | Moderate |
|  |  | post-operative blood loss | 8 | 612 | RCT | Not serious | Very Serious Inconsistency | Not serious | no Serious Risk | not Suspected | would not  reduce effect | No | No | Low |
|  |  | blood transfusion requirements | 10 | 722 | RCT | Not serious | Very Serious Inconsistency | Not serious | no Serious Risk | not Suspected | would not  reduce effect | No | No | Low |
|  |  | blood transfusion rate | 12 | 815 | RCT | Not serious | no Serious Inconsistency | Not serious | no Serious Risk | not Suspected | would not  reduce effect | No | No | Moderate |
|  |  | incidence of deep vein thrombosis | 17 | 1191 | RCT | Not serious | no Serious Inconsistency | Not serious | no Serious Risk | not Suspected | would not  reduce effect | Yes | No | Moderate |
| HMB | Bryant-Smith 2018 | menstrual blood loss:mean loss-antifibrinolytics compared to no treatment or placebo(ml) | 4 | 565 | RCT | Not serious | no Serious Inconsistency | Not serious | no Serious Risk | Suspected | would not  reduce effect | No | No | Moderate |
|  |  | menstrual blood loss:improvement rates-antifibrinolytics compared to no treatment or placebo(ml) | 3 | 271 | RCT | Not serious | no Serious Inconsistency | Not serious | no Serious Risk | Suspected | would not  reduce effect | Yes | No | High |
|  |  | quality of life scores-antifibrinolytics compared to no treatment or placebo | 2 | 365 | RCT | Not serious | no Serious Inconsistency | Not serious | no Serious Risk | Suspected | would not  reduce effect | No | No | Low |
|  |  | adverse events-antifibrinolytics compared to no treatment or placebo | 1 | 297 | RCT | Serious | no Serious Inconsistency | Not serious | Serious Risk | Suspected | would not  reduce effect | No | No | Very low |
|  |  | antifibrinolytic agent versus  progestogens-menstrual blood loss: mean PBAC score | 3 | 312 | RCT | Not serious | no Serious Inconsistency | Not serious | Serious Risk | Suspected | would not  reduce effect | No | No | Low |
|  |  | antifibrinolytic agent versus progestogens-menstrual blood loss:improvement rates | 5 | 422 | RCT | Not serious | no Serious Inconsistency | Not serious | no Serious Risk | Suspected | would not  reduce effect | No | No | Moderate |
|  |  | antifibrinolytic agent versus  progestogens-menstrual blood loss:improvement rates | 6 | unclear | RCT | Not serious | no Serious Inconsistency | Not serious | no Serious Risk | no Suspected | would not  reduce effect | No | No | High |
|  |  | antifibrinolytic agent versus progestogens- adverse events | 1 | 49 | RCT | serious | no Serious Inconsistency | Not serious | no Serious Risk | suspected | would not  reduce effect | No | No | Low |
|  |  | antifibrinolytic agent versus NSAIDs-menstrual blood loss: mean loss | 1 | 161 | RCT | serious | no Serious Inconsistency | Not serious | no Serious Risk | Suspected | would not  reduce effect | No | No | Low |
|  |  | antifibrinolytic agent versus NSAIDs-menstrual blood loss: improvement rates | 1 | 53 | RCT | serious | no Serious Inconsistency | Not serious | no Serious Risk | Suspected | would not  reduce effect | No | No | Low |
|  |  | antifibrinolytic agent versus ethamsylate-menstrual blood loss: mean loss | 1 | 53 | RCT | serious | no Serious Inconsistency | Not serious | no Serious Risk | Suspected | would not  reduce effect | No | No | Low |
|  |  | antifibrinolytic agent versus ethamsylate-menstrual blood loss:improvement rates | 2 | 121 | RCT | Not serious | no Serious Inconsistency | Not serious | no Serious Risk | Suspected | would not  reduce effect | No | No | Moderate |
| cancer | Montroy 2017 | venous thromboembolic events | 9 | 1075 | RCT | Not serious | no Serious Inconsistency | Not serious | Serious Risk | not Suspected | would not  reduce effect | No | No | Moderate |
|  |  | blood transfusion | 7 | 955 | RCT | Not serious | Serious Inconsistency | Not serious | no Serious Risk | not Suspected | would not  reduce effect | No | No | Moderate |
|  |  | blood loss | 9 | 1109 | RCT | Not serious | Very Serious Inconsistency | Not serious | no Serious Risk | not Suspected | would not  reduce effect | No | No | Low |

IV= intravenous, THA=total hip arthroplasty, MOS=major orthopedic surgery

HMB=heavy menstrual bleeding, RCT=randomized controlled study

## Appendix 12. Outcomes of blood loss

Appendix 12a. Outcomes of total blood loss (ml) with weighted mean difference (WMD)

| **Author Year** | **Surgery** | **Agent** | **Characteristic** | **subgroup** | **RCTs** | **Patients** | 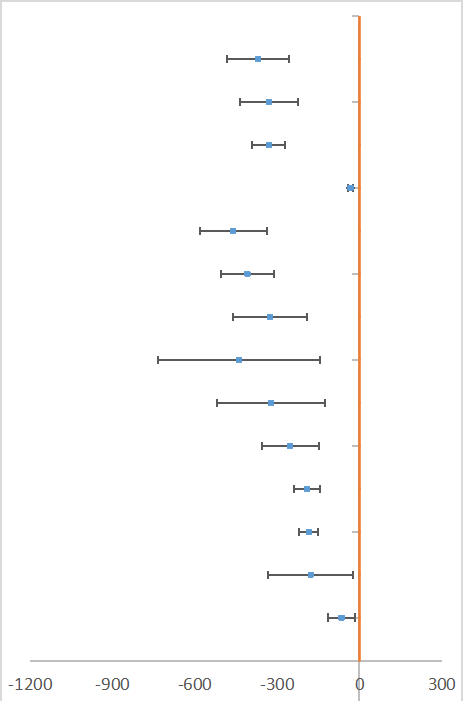 | **95% CI Estimate (WMD)** | **GRADE** |
| --- | --- | --- | --- | --- | --- | --- | --- | --- | --- |
| Huang 2015 | THA | Inconsistent | IV/topical | / | 11 | 687 |  | -369.17 [-481.73, -256.61] | Moderate |
| Huang 2013 | THA | Inconsistent | IV/topical/oral | / | 9 | 488 |  | -331.00 [-436.35, -225.65] | Moderate |
| Yao 2019 | PAO | Inconsistent | IV | / | 3 | 333 |  | -330.49 [-390.16, -270.83] | Moderate |
| Chan 2012 | tonsillectomy | 10mg/kg | IV/topical | / | 2 | 180 |  | -32.72 [-42.66, -22.78] | Moderate |
| Huang 2013 | TKA | Inconsistent | IV/topical/oral | / | 14 | 873 |  | -459.82 [-582.08, -337.56] | Low |
| Huang 2013 | MOS | Inconsistent | IV/topical/oral | / | 24 | 1696 |  | -408.33 [-505.69, -310.97] | Low |
| Xiao 2019 | hip fracture | 10-15mg/kg | IV | / | 6 | 416 |  | -326.64 [-462.23, -191.06] | Low |
| Huang 2013 | spine surgery | Inconsistent | IV/topical/oral | / | 5 | 335 |  | -438.57 [-734.51, -142.62] | Very low |
| Wu 2017 | TKA | 10 mg/kg | IV | / | 4 | 332 |  | -322.96 [-519.52, -126.40] | Very low |
| Yao 2019 | PAO/HTO | Inconsistent | IV/topical | / | 3 | 332 |  | -252.50 [-356.81, -148.18] | Very low |
| Wang 2019 | caesarean section | 10mg/kg | IV | elective surgery | 6 | 1994 |  | -191.34 [-237.72, -144.96] | Very low |
| Wang 2019 | caesarean section | 10mg/kg | IV | any surgery | 11 | 2777 |  | -184.88 [-218.83, -150.94] | Very low |
| Zhou X 2019 | IFS | Inconsistent | IV | / | 4 | 309 |  | -177.83 [-332.49, -23.18] | Very low |
| Xia 2020 | vaginal delivery | 1g | IV | / | 4 | 4579 |  | -65.61 [-115.01, -16.21] | Very low |
|  | | | | | | |  |  | |

PAO=periacetabular osteotomy; MOS=major orthopedic surgery (TKA, THA and spine surgery); IFS=intertrochanteric fracture surgery.

Appendix 12b. Outcomes of intraoperative blood loss (ml) with weighted mean difference (WMD)

| **Author Year** | **Surgery** | **Agent** | **Characteristic** | **subgroup** | **RCTs** | **Patients** | **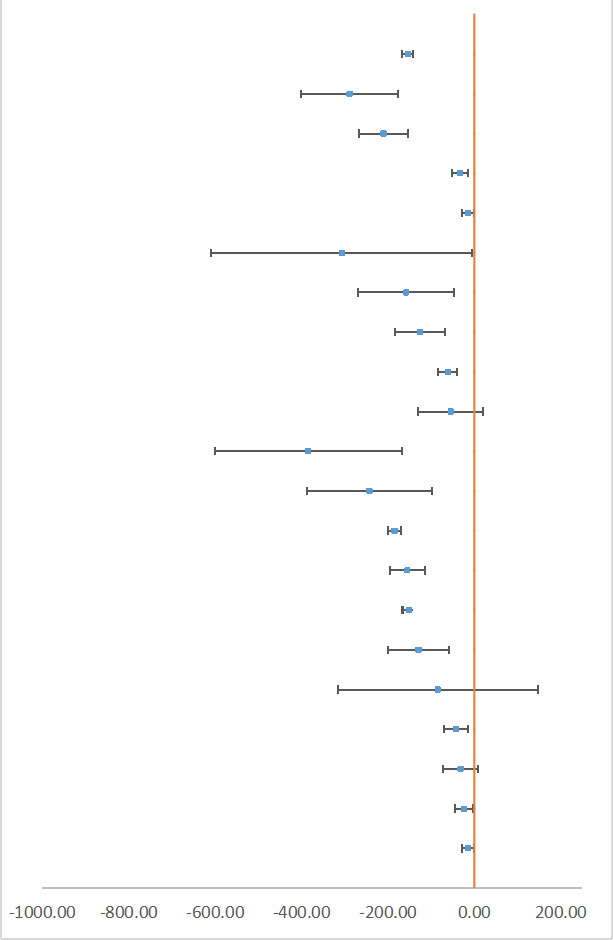** | **95% CI Estimate (WMD)** | **GRADE** |
| --- | --- | --- | --- | --- | --- | --- | --- | --- | --- |
| Zhao 2019 | OS | 10-20mg/kg | IV | total | 10 | 655 |  | -153.97 [-166.52, -141.41] | **High** |
| Zhao 2019 | OS | 10-20mg/kg | IV | 20mg/kg | 3 | 133 |  | -288.90 [-400.86, -176.94] | Moderate |
| Chen 2021 | spine surgery | 5-30mg/kg | IV | / | 7 | 587 |  | -210.38 [-267.31, -153.45] | Moderate |
| Luo 2019 | IFS | 1-3g | topical | / | 4 | 339 |  | -33.46 [-52.40, -14.52] | Moderate |
| Xia 2020 | vaginal delivery | 10-15mg/kg | IV | / | 3 | 4140 |  | -14.30 [-28.39, -0.22] | Moderate |
| Huang 2013 | spine surgery | Inconsistent | IV/topical/oral | / | 6 | 407 |  | -306.87 [-182.58, -68.72] | Low |
| Huang 2013 | THA | Inconsistent | IV/topical/oral | / | 8 | 463 |  | -157.74 [-268.46, -47.03] | Low |
| Huang 2013 | MOS | Inconsistent | IV/topical/oral | total | 21 | 1259 |  | -125.65 [-182.58, -68.72] | Low |
| Vasconcellos 2018 | rhinoplasty | Inconsistent | Oral | / | 2 | 100 |  | -61.70 [-83.02, -40.39] | Low |
| Huang 2013 | TKA | Inconsistent | IV/topical/oral | / | 7 | 389 |  | -54.62 [-129.52, 20.29] | Low |
| Zhao 2019 | OS | 10-20mg/kg | IV | 15mg/kg | 1 | 40 |  | -385.00 [-601.42, -168.58] | Very low |
| Zhao 2019 | OS | 10-20mg/kg | IV | fixed-1000mg | 1 | 56 |  | -242.85 [-386.72, -98.98] | Very low |
| Wang 2019 | caesarean section | 10mg/kg | IV | Elective surgery | 5 | 634 |  | -184.96 [-200.43, -169.50] | Very low |
| Wang 2019 | caesarean section | 10mg/kg | IV | / | 14 | 1665 |  | -155.23 [-195.64, -114.81] | Very low |
| Zhao 2019 | OS | 10-20mg/kg | IV | 10mg/kg | 3 | 290 |  | -151.67 [-164.40, -168.58] | Very low |
| Zhao 2019 | OS | 10-20mg/kg | IV | fixed-1000mg | 1 | 96 |  | -129.21 [-199.89, -58.53] | Very low |
| Zhao 2019 | OS | 10-20mg/kg | IV | fixed-500mg | 1 | 40 |  | -85.01 [-316.64, 146.62] | Very low |
| Vasconcellos 2018 | rhinoplasty | Inconsistent | IV/oral | / | 4 | 246 |  | -42.28 [-70.36, -14.21] | Very low |
| Zhou 2019 | IFS | Inconsistent | IV | / | 4 | 309 |  | -31.89 [-73.32, 9.53] | Very low |
| Vasconcellos 2018 | rhinoplasty | Inconsistent | IV | / | 2 | 146 |  | -23.88 [-45.19, -2.58] | Very low |
| Zhang 2019 | calcaneal fracture | 10-15mg/kg | IV | / | 4 | 283 |  | -14.54 [-28.08, -1.00] | Very low |
|  | | | | | | |  |  | |

OS=orthognathic surgery; MOS=major orthopedic surgery (TKA, THA and spine surgery); IFS=intertrochanteric fracture surgery.

Appendix 12c. Outcomes of postoperative blood loss (ml) with weighted mean difference (WMD)

| **Author Year** | **Surgery** | **Agent** | **Characteristic** | **subgroup** | **RCTs** | **Patients** | **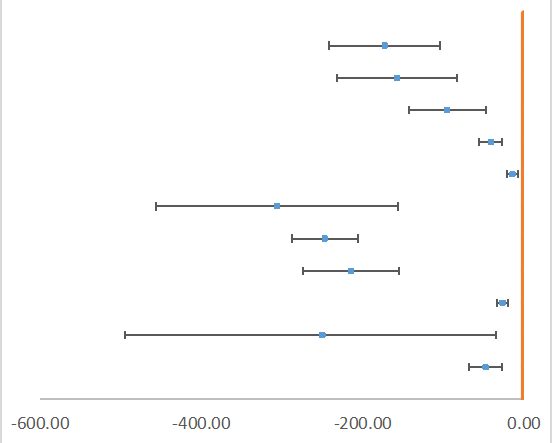** | **95% CI Estimate (WMD)** | **GRADE** |
| --- | --- | --- | --- | --- | --- | --- | --- | --- | --- |
| Luo 2019 | IFS | Inconsistent | IV | / | 4 | 339 |  | -172.83 [-241.43, -104.23] | Moderate |
| Huang 2013 | THA | Inconsistent | IV/topical/oral | / | 8 | 416 |  | -157.85 [-232.36, -83.34] | Moderate |
| Huang 2013 | spine surgery | Inconsistent | IV/topical/oral | / | 3 | 251 |  | -95.12 [-142.65, -47.60] | Moderate |
| Xia 2020 | vaginal delivery | 1g | IV | / | 2 | 301 |  | -41.24 [-55.50, -26.98] | Moderate |
| Zhou 2019 | IFS | Inconsistent | IV | / | 3 | 314 |  | -14.38 [-20.83, -7.93] | Moderate |
| Huang 2013 | TKA | Inconsistent | IV/topical/oral | / | 7 | 600 |  | -306.60 [-456.54, -156.67] | Low |
| Guo 2019 | cardiac surgery | Inconsistent | IV/topical | / | 44 | 5560 |  | -246.98 [-287.89, -206.06] | Low |
| Huang 2013 | MOS | Inconsistent | IV/topical/oral | / | 19 | 1267 |  | -214.58 [-274.63, -154.52] | Low |
| Wang 2019 | caesarean section | 10mg/kg | IV | / | 12 | 1398 |  | -26.67 [-32.98, -20.36] | Low |
| Abrishami 2009 | cardiac surgery | 1-2.5g | topical | / | 4 | 269 |  | -249.98 [-495.15, -34.81] | Very low |
| Wang 2019 | caesarean section | 10mg/kg | IV | elective surgery | 4 | 514 |  | -47.66 [-67.66, -27.67] | Very low |
|  | | | | | | |  |  | |

IFS=intertrochanteric fracture surgery; MOS=major orthopedic surgery (TKA, THA and spine surgery).

Appendix 12d. Outcomes of blood loss with standard mean difference (SMD)

| **Author Year** | **Surgery** | **Agent** | **Characteristic** | **Outcome** | **RCTs** | **Patients** | **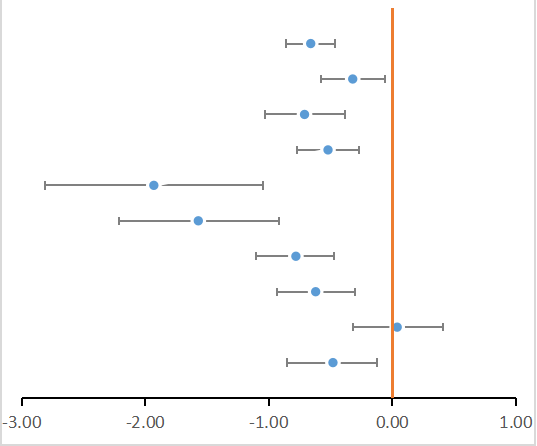** | **95% CI Estimate (SMD)** | **GRADE** |
| --- | --- | --- | --- | --- | --- | --- | --- | --- | --- |
| Kim 2019 | endoscopic sinus | 10-15mg/kg | IV | IBL | 7 | 562 |  | -0.66 [-0.86, -0.46] | **High** |
| Du 2018 | spinal fusion | 10-30mg/kg | IV/topical | IBL | 6 | 394 |  | -0.32 [-0.58, -0.06] | **High** |
| Kang 2019 | endoscopic sinus | 1-2g | topical | IBL | 4 | 226 |  | -0.71 [-1.03, -0.38] | Moderate |
| Du 2019 | spinal fusion | 10-30mg/kg | IV | IBL | 4 | 254 |  | -0.52 [-0.77, -0.27] | Moderate |
| Longo 2018 | prostate surgery | 10-15mg/kg | IV/oral/local spray | IBL | 6 | 738 |  | -1.93 [-2.81, -1.05] | Low |
| Montroy 2017 | cancer | Inconsistent | IV/topical | TBL | 9 | 1109 |  | -1.57 [-2.21, -0.92] | Low |
| Li 2017 | spine surgery | 10-100mg/kg | IV | TBL | 8 | 560 |  | -0.78 [-1.10, -0.47] | Low |
| Li 2017 | spine surgery | 10-100mg/kg | IV | IBL | 8 | 511 |  | -0.62 [-0.93, -0.30] | Low |
| Du 2020 | spinal fusion | 1-2g | topical | IBL | 2 | 140 |  | 0.04 [-0.32, 0.41] | Low |
| Li 2017 | spine surgery | 10-100mg/kg | IV | PBL | 5 | 330 |  | -0.48 [-0.85, -0.12] | Very low |
|  | | | | | | |  |  | |

IBL=intraoperative blood loss; PBL=postoperative blood loss; TBL=total blood loss.

## Appendix 13. Operative time and length of stay

Appendix 13a. Outcomes of re-operation rate

| Agent | Surgery | Author Year | Characteristic | subgroup | RCTs | Patients | Evidence synthesis | P value | GRADE |
| --- | --- | --- | --- | --- | --- | --- | --- | --- | --- |
| TXA | coronary artery bypass grafting surgery | Zhang 2019 | IV | total pump | 16 | 6259 | RR 0.46[0.31,0.68] | **<0.0001*** | **High** |
| TXA | cardiac surgery | Guo 2019 | IV/topical | / | 32 | 8937 | RR 0.62[0.49,0.79] | **<0.0001*** | **High** |
| TXA | coronary artery bypass grafting surgery | Zhang 2019 | IV | on-pump | 11 | 1194 | RR 0.60[0.34,1.07] | 0.08 | Moderate |
| TXA | coronary artery bypass grafting surgery | Zhang 2019* | IV | off-pump | 5 | 435 | RR 0.33[0.01,7.90] | 0.50 | Low |
| TXA | coronary artery bypass grafting surgery | Zhang 2019 | IV | mixed-pump | 1 | 4630 | RR 0.38[0.22,0.65] | **0.0004^*^** | Low |
| TXA | Upper gastrointestinal bleeding | Twum-Barimah 2020 | IV/oral/topical | / | 9 | 1863 | RR 0.70[0.43,1.13] | 0.14 | Low |
| TXA | traumatic brain injury | July 2020 | IV | / | 4 | 10566 | RR 0.99[0.89,1.12] | 0.93 | Low |
| TXA | acute traumatic injury | Ker 2015 | IV | / | 2 | 20367 | RR 1.00[0.97,1.03] | 0.83 | Low |

*P value≤0.05

Appendix 13b. Outcomes of operation time

| Agent | Surgery | Author Year | Characteristic | Outcome | subgroup | RCTs | Patients | Evidence synthesis | P value | GRADE |
| --- | --- | --- | --- | --- | --- | --- | --- | --- | --- | --- |
| TXA | orthognathic surgery | Zhao 2019 | intravenous | operation time(min) | bimaxillary | 7 | 455 | WMD -8.88[-16.69,-1.07] | **0.03*** | **High** |
| TXA | orthognathic surgery | Zhao 2019 | intravenous | operation time(min) | total | 8 | 655 | WMD -16.18[-19.60, -12.75] | **<0.00001*** | **High** |
| TXA | endoscopic sinus surgery | Kim 2019 | intravenous | operative time | / | 4 | 232 | SMD -0.60 [-0.93, -0.27] | **0.0003*** | Moderate |
| TXA | spine surgery | Chen 2021 | intravenous | operation time(min) |  | 6 | 509 | WMD -7.75[-16.65,1.15] | 0.09 | Moderate |
| TXA | endoscopic sinus surgery | Kang 2019 | topical | operative time | / | 2 | 110 | SMD -0.25 [-0.71, 0.21] | 0.2954 | Low |
| TXA | craniosynostosis open surgery | Lu 2019 | intravenous | operation time(min) | / | 7 | unclear | WMD -0.90[-6.61,4.81] | 0.757 | Very low |

*P value≤0.05

Appendix 13c. Outcomes of length of stay

| Agent | Surgery | Author Year | Characteristic | Outcome | subgroup | RCTs | Patients | Evidence synthesis | P value | GRADE |
| --- | --- | --- | --- | --- | --- | --- | --- | --- | --- | --- |
| TXA | hemoptysis | Tsai 2020 | intravenous | length of stay(d) | / | 2 | 113 | WMD -1.62[-2.93,-0.31] | **0.02*** | Moderate |
| TXA | spinal fusion surgery | Du 2018 | intravenous or topical | length of stay | / | 2 | 140 | SMD -1.00[-1.68,-0.32] | **0.004*** | Low |
| TXA | spine surgery | Chen 2021 | intravenous | length of stay(d) | / | 5 | 426 | WMD -1.09[-1.86,-0.32] | **0.006*** | Low |
| TXA | craniosynostosis open surgery | Lu 2019 | intravenous | length of stay(d) | ICU | 3 | unclear | WMD -0.17[-0.53,0.19] | 0.349 | Very low |
| TXA | craniosynostosis open surgery | Lu 2019 | intravenous | length of stay(d) | hospital | 6 | unclear | WMD -0.34[-0.76,0.07] | 0.102 | Very low |

*P value≤0.05

## Appendix 14. Hb and coagulation function

Appendix 14a. Outcomes of postoperative hemoglobin (Hb)

| Author Year | Surgery | Agent | Characteristic | Outcome | subgroup | RCTs | Patients | Evidence synthesis | P value | GRADE |
| --- | --- | --- | --- | --- | --- | --- | --- | --- | --- | --- |
| Chen 2021 | spine surgery | 5-30mg/kg | IV | postoperative Hb | / | 6 | 492 | SMD 0.20[0.02,0.38] | **0.03*** | **High** |
| Longo 2018 | prostate surgery | 10-15mg/kg | IV/oral/local spray | postoperative Hb within 24h | TURP | 2 | 140 | SMD 0.32[-0.04,0.68] | 0.08 | Low |
| Luo 2019 | intertrochanteric fracture surgery | Inconsistent | IV | postoperative Hb within 3d (g/dL) | / | 3 | 364 | WMD 0.32[-0.09,0.74] | 0.13 | Low |
| Zhang 2019 | calcaneal fracture surgery | 10-15mg/kg | IV | postoperative Hb | / | 4 | 287 | SMD 1.21[0.38,2.04] | **0.004*** | Very low |
| Longo 2018 | prostate surgery | 10-15mg/kg | IV/oral/local spray | postoperative Hb within 24h | total | 4 | 526 | SMD 0.22[-0.05,0.48] | 0.11 | Very low |
| Longo 2018 | prostate surgery | 10-15mg/kg | IV/oral/local spray | postoperative Hb within 24h | prostatectomy | 2 | 386 | SMD 0.14[-0.28,0.56] | **0.50*** | Very low |
| Du 2018 | spinal fusion surgery | 10-30mg/kg | IV/topical | postoperative Hb | / | 4 | 254 | SMD -0.10[-0.56,0.37] | 0.68 | Very low |

*P value≤0.05

Appendix 14b. Outcomes of postoperative hemoglobin (Hb) drop

| Author Year | Surgery | Agent | Characteristic | Outcome | subgroup | RCTs | Patients | Evidence synthesis | P value | GRADE |
| --- | --- | --- | --- | --- | --- | --- | --- | --- | --- | --- |
| Yao 2019 | periacetabular osteotomy | Inconsistent | IV/topical | Hb drop (g/dL) | / | 2 | 233 | WMD -0.56[-0.94,-0.17] | **0.004*** | Moderate |
| Guo 2018 | total knee arthroplasty | 1g | oral | Hb drop | / | 3 | 515 | SMD -0.936[-1.118,-0.754] | **<0.001*** | Moderate |
| Chen 2014 | total knee arthroplasty | 0.5-3g | topical | Hb drop (g/dL) | / | 4 | 298 | WMD 0.63[-0.96,0.31] | **0.024*** | Low |
| Longo 2018 | prostate surgery | 10-15mg/kg | IV/oral/ local spray | Hb drop within 24h | total | 4 | 462 | SMD -0.06[-0.24,0.13] | 0.54 | Low |
| Longo 2018 | prostate surgery | 10-15mg/kg | IV/oral/local spray | Hb drop within 24h | TURP | 3 | 276 | SMD -0.08[-0.32,0.16] | 0.52 | Low |
| Wang 2019 | caesarean section | 10mg/kg | IV | Hb drop (g/dL) | elective surgery | 6 | 1374 | WMD -0.73 [-0.86, -0.61] | **<0.0001*** | Low |
| Yao 2019 | periacetabular osteotomy and high tibial osteotomy | Inconsistent | IV/topical | Hb drop (g/dL) | / | 5 | 565 | WMD -0.74[-1.09,-0.38] | **<0.0001*** | Low |
| Longo 2018 | prostate surgery | 10-15mg/kg | IV/oral/local spray | Hb drop within 24h | prostatectomy | 1 | 186 | SMD -0.03[-0.31,0.26] | 0.86 | Very low |
| Kirsch 2017 | total shoulder arthroplasty | 10-20mg/kg | IV/topical | Hb drop (g/dL)) | / | 5 | 632 | WMD -0.64[-0.84,-0.44] | **<0.00001*** | Very low |
| Wang 2019 | caesarean section | 10mg/kg | IV | Hb drop (g/dL) | / | 9 | 1807 | WMD -0.80 [-1.07, -0.53] | **<0.0001*** | Very low |
| Yao 2019 | high tibial osteotomy | Inconsistent | IV/topical | Hb drop (g/dL) | / | 3 | 232 | WMD -0.82[-1.34,-0.30] | **0.005*** | Very low |

*P value≤0.05

Appendix 14c. Outcomes of coagulation function

| Author Year | Surgery | Agent | Characteristic | Outcome | subgroup | RCTs | Patients | Evidence synthesis | P value | GRADE |
| --- | --- | --- | --- | --- | --- | --- | --- | --- | --- | --- |
| Zhang S 2019 | calcaneal fracture surgery | 10-15mg/kg | intravenous | partial thromboplastin time(s) | / | 6 | 409 | WMD 1.08[-0.17,2.33] | 0.09 | Moderate |
| Kim 2019 | endoscopic sinus surgery | 10-15mg/kg | Intravenous | prothrombin time | / | 3 | 254 | SMD 0.16 [-0.19, 0.50] | 0.3739 | low |
| Kim 2019 | endoscopic sinus surgery | 10-15mg/kg | Topical | prothrombin time | / | 2 | 110 | SMD -0.01 [-0.38, 0.36] | 0.9555 | low |
| Kang 2019 | endoscopic sinus surgery | 1-2g | Topical | partial thromboplastin time | / | 2 | 110 | SMD -0.32 [-0.69, 0.06] | 0.0973 | Low |
| Zhang S 2019 | calcaneal fracture surgery | 10-15mg/kg | intravenous | prothrombin time(s) | / | 5 | 349 | WMD 0.06[-0.45,0.57] | 0.82 | Very low |
| Kim 2019 | endoscopic sinus surgery | 10-15mg/kg | Intravenous | partial thromboplastin time | / | 3 | 254 | SMD -0.17 [-0.62, 0.27] | 0.4484 | Very low |

*P value≤0.05

## Appendix 15. Outcomes of comparison between different routes of TXA in major orthopedic surgery

Appendix 15a. Outcomes of comparison between single route TXA in total joint arthroplasty

| Author Year | Comparison | Outcome | Surgery | RCTs | Patients | 95% CI Estimate | I^2^ | P value | GRADE |
| --- | --- | --- | --- | --- | --- | --- | --- | --- | --- |
| Li 2020 | IV vs topical | Total blood loss | TKA | 13 | 1197 | WMD 33.38 [19.24, 47.51] | 11.0% | <0.001* | High |
|  |  | Transfusion rate | TKA | 25 | 2950 | OR 0.93 [0.69, 1.24] | 0.0% | 0.62 | Moderate |
|  |  | All adverse event | TKA | 20 | 2594 | OR 1.00 [0.72, 1.39] | 0.0% | 0.98 | Moderate |
|  |  | DVT | TKA | 10 | 1641 | OR 0.92 [0.44, 1.92] | 0.0% | 0.83 | Moderate |
|  |  | PE | TKA | 3 | 342 | OR 1.02 [0.25, 4.20] | 0.0% | 0.98 | Low |
| Sun 2019 | IV vs topical | Total blood loss | TKA | 10 | / | WMD 52.69 [-18.58,123.97] | 89.9% | 0.15 | Very low |
|  |  |  | THA | 4 | / | WMD -31.03 [-156.16,94.10] | 70.3% | 0.63 | Very low |
|  |  | Transfusion rate | TKA | 12 | / | RR 1.25 [0.80,1.96] | 8.3% | 0.32 | Moderate |
|  |  |  | THA | 5 | / | RR 0.80 [0.46,1.37] | 0.0% | 0.41 | Low |
|  |  | DVT | TKA | 5 | / | RR 2.14 [0.74,6.18] | 0.0% | 0.16 | Moderate |
|  |  |  | THA | 4 | / | RR 1.45 [0.30,6.93] | 0.0% | 0.64 | Low |
| Chen 2016 | IV vs topical | Total blood loss | TKA | 8 | 688 | WMD -28.72 [-195.97,138.54] | 97.0% | 0.74 | Very low |
|  |  |  | THA | 4 | 550 | WMD 38.66 [-38.97,116.30] | 60.0% | 0.33 | Very low |
|  |  | Transfusion rate | TKA | 12 | 1026 | OR 0.90 [0.58,1.40] | 30.0% | 0.64 | Moderate |
|  |  |  | THA | 4 | 550 | OR 1.19 [0.67,2.09] | 0.0% | 0.63 | Low |
|  |  | Infection | TKA | 2 | 160 | OR 1.00 [0.14,7.24] | 0.0% | 1.00 | Low |
|  |  |  | THA | 1 | 140 | OR 1.52 [0.25,9.49] | / | 0.65 | Very low |
|  |  | DVT | TKA | 9 | 640 | OR 1.10 [0.45,2.68] | 0.0% | 0.83 | Moderate |
|  |  |  | THA | 3 | 383 | OR 0.23 [0.05,1.10] | 0.0% | 0.07 | Moderate |
|  |  | PE | THA | 3 | 482 | OR 0.33 [0.01,8.32] | 0.0% | 0.50 | Moderate |
| Ye 2020 | Oral vs IV | Total blood loss | TKA or THA | 7 | 692 | WMD -3.67[-45.12,37.78] | 0.0% | 0.86 | Moderate |
|  |  | DVT | TKA or THA | 4 | 626 | OR 0.37[0.10,1.40] | 0.0% | 0.14 | Moderate |
|  |  | Transfusion rate | TKA or THA | 9 | 934 | OR 1.03[0.65,1.61] | 0.0% | 0.91 | Moderate |

DVT=deep venous thrombosis; PE=pulmonary embolism; TKA=total knee arthroplasty; THA=total hip arthroplasty

*P value ≤ 0.05

Appendix 15b. Outcomes of comparison between combined and single route TXA in total joint arthroplasty

| Author Year | Comparison | Outcome | Surgery | RCTs | Patients | 95% CI Estimate | I^2^ | P value | GRADE |
| --- | --- | --- | --- | --- | --- | --- | --- | --- | --- |
| Sun 2019 | Combined vs Single | Total blood loss | TKA or THA | 7 | / | WMD -198.07 [-307.67,-88.46] | 92.3% | <0.05* | Low |
|  |  |  | TKA | 4 | / | WMD -168.34 [-251.25,-85.44] | 59.4% | <0.05* | Low |
|  |  |  | THA | 3 | / | WMD -210.36 [-407.39,-13.34] | 96.3% | <0.05* | Very low |
|  |  | Transfusion rate | TKA or THA | 7 | / | RR 0.40 [0.24,0.68] | 0.0% | <0.05* | High |
|  |  |  | TKA | 4 | / | RR 0.48 [0.16,1.39] | 0.0% | <0.05* | High |
|  |  |  | THA | 3 | / | RR 0.38 [0.20,0.69] | 0.0% | <0.05* | High |
|  |  | DVT | TKA or THA | 6 | / | RR 1.25 [0.43,3.70] | 0.0% | 0.68 | Moderate |
|  |  |  | TKA | 3 | / | RR 0.34 [0.04,3.23] | 0.0% | 0.34 | Moderate |
|  |  |  | THA | 3 | / | RR 1.85 [0.54,6.25] | 0.0% | 0.32 | Low |
| Zhang 2017 | Combined vs Single | Total blood loss | THA | 7 | 802 | WMD -146.29 [-206.66,-85.92] | 83.0% | <0.001* | Low |
|  |  | Intraoperative blood loss | THA | 5 | 654 | WMD -64.65 [-74.75,-54.55] | 82.0% | <0.001* | Very low |
|  |  | Transfusion rate | THA | 7 | 668 | RR 0.41 [0.27,0.63] | 84.0% | <0.001* | Moderate |
|  |  | DVT | THA | 8 | 950 | RR 1.22 [0.52,2.89] | 0.0% | 0.65 | Moderate |
| Zhang 2016 | Combined vs Single | Total blood loss | TKA | 3 | 363 | WMD -189.75 [-306.94,-72.56] | 70.0% | 0.002* | Low |
|  |  |  | THA | 3 | 280 | WMD -95.62 [-129.63,-61.60] | 0.0% | <0.001* | Moderate |
|  |  | Transfusion rate | TKA&THA | 6 | 643 | RR 0.42 [0.20,0.85] | 9.0% | 0.02* | High |
|  |  | DVT | TKA&THA | 3 | 424 | RR 1.00 [0.28,3.63] | 0.0% | 0.99 | Low |

DVT=deep venous thrombosis; PE=pulmonary embolism; TKA=total knee arthroplasty; THA=total hip arthroplasty

*P value ≤ 0.05

Appendix 15c. Outcomes of comparison between IV and topical TXA in spine surgery

| Author Year | Comparison | Outcome | Surgery | RCTs | Patients | 95% CI Estimate | I^2^ | P value | GRADE |
| --- | --- | --- | --- | --- | --- | --- | --- | --- | --- |
| Xiong 2020 | IV vs topical | intraoperative blood loss | Spine surgery | 4 | 336 | WMD -32.72 [-129.17,63.72] | 97.0% | 0.51 | Very low |
|  |  | Hidden blood loss | Spine surgery | 3 | 266 | WMD -76.73 [-178.30,24.84] | 94.0% | 0.14 | Very low |
|  |  | Total blood loss | Spine surgery | 3 | 358 | WMD -69.65 [-149.93,10.64] | 73.0% | 0.09 | Very low |
|  |  | Transfusion rate | Spine surgery | 4 | 274 | RR 0.91[0.60,1.40] | 0.0% | 0.68 | Low |
